# Greener Solvents for the Photoisomerization and Catalytic Heat Release of a Norbornadiene‐Based Molecular Solar Thermal Energy Storage System

**DOI:** 10.1002/advs.77637

**Published:** 2026-09-11

**Authors:** Lucien Magson, Helen Hölzel, Rebecca J. Salthouse, Liang Fei, Pedro Ferreira, Vitor A. S. Almodovar, Kasper Moth‐Poulsen, Ignacio Funes‐Ardoiz, Diego Sampedro

**Affiliations:** ^1^ Instituto de Investigación en Química (IQUR) Universidad de La Rioja Logroño, La Rioja Spain; ^2^ Institute of Organic Chemistry Justus‐Liebig‐University Giessen Giessen Germany; ^3^ Department of Chemical Engineering Universitat Politècnica de Catalunya, EEBE Barcelona Spain; ^4^ Department of Chemistry and Chemical Engineering Chalmers University of Technology Gothenburg Sweden; ^5^ Catalan Institution for Research & Advanced Studies ICREA Barcelona Spain; ^6^ Institute of Materials Science of Barcelona ICMAB‐CSIC Barcelona Spain

**Keywords:** catalysis, energy storage, heat release, norbornadiene, sustainable

## Abstract

Molecular Solar Thermal (MOST) energy storage employs photoswitches to offer a closed‐cycle means of capturing, storing, and utilizing solar energy in an emission‐free system. In this work, we conduct a systematic solvent screening to elucidate how different media affect the photoisomerization of a norbornadiene–quadricyclane (NBD–QC) photoswitch and the subsequent catalytic activation of the heat‐release step for efficient, on‐demand energy delivery. For future applications, all system components, including the photoswitch, catalyst, and solvent, should be non‐toxic and environmentally benign. Among the greener solvents assessed here, the photoisomerization quantum yield was observed to be 25% points higher and the storage lifetime extended by 15 days in the biodegradable solvent propylene carbonate (Pc) compared with toluene, the most commonly used solvent in MOST systems. Using a custom‐built setup, a heat‐release temperature increase of 51°C was achieved in Pc, approaching the highest temperature increase reported for MOST systems operating in organic solvents. Pc also displays broad compatibility with a range of catalysts, including those based on first‐row transition and noble metals. We thus present a fully integrated MOST/catalyst/solvent system comprising a scalable NBD‐QC photoswitch, an active catalyst (including naturally abundant metals), and a low‐toxicity, sustainable solvent. Together, these features represent a significant advancement in the feasibility of MOST systems for practical applications.

## Graphical abstract

A Molecular Solar Thermal (MOST) system combining a scalable norbornadiene–quadricyclane photoswitch, a biodegradable solvent, propylene carbonate, and noble and first‐row metal catalysts, is presented. Propylene carbonate boosts photoisomerization quantum yield by 25% compared to toluene, prolongs energy storage by an additional 15 days, and enables heat release up to 51°C, advancing the practical feasibility of MOST systems in safe and sustainable solar thermal storage.

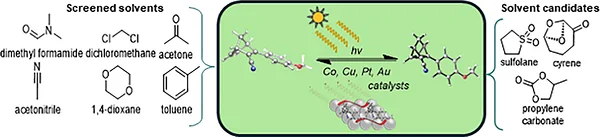

## 1 Introduction

According to the World Energy Outlook 2024, buildings account for approximately 30% of global energy consumption, with space and water heating representing 77.6% of the final energy consumed by households in 2023 [1, 2]. Molecular solar thermal (MOST) energy storage systems offer a low‐carbon approach to solar energy storage, addressing this demand by storing solar energy in chemical bonds and releasing it as heat on demand within a closed cycle [3]. Recent advances in solid‐state phase change MOST architectures have been promising, including azobenzenes for molecular solar thermal fabrics [4] and pyrimidones for water heating [5]. The ability to operate MOST materials in the liquid state is particularly attractive for district heating or industrial process heating applications, as it enables pumping and direct integration with existing heat‐transfer infrastructure. One of the most widely studied MOST systems is the norbornadiene (NBD)‐quadricyclane (QC) photoswitch couple. Upon absorption of light, NBD undergoes a [2+2] cycloaddition to form the metastable QC isomer, thereby storing solar energy as chemical energy. This stored energy can be released as heat through the exothermic back‐conversion to NBD, which can be triggered, thermally, catalytically [6, 7], electrochemically [8, 9], or photochemically [10, 11]. The NBD‐QC system is particularly attractive due to its high energy density and tunable photochemical properties.

Among the numerous requirements for an optimal MOST photoswitch [12], the choice of solvent is particularly important for applications in which flow operation is required, as MOST systems typically operate in solution. An ideal solvent must dissolve the photoswitch at high concentrations (ideally >1.5 M) to achieve the target energy density (>0.3 MJ kg^−1^) while remaining photochemically inert and optically transparent in the absorption region of the photoswitch. For potential applications in space heating, such as underfloor heating systems, conventional radiators, and pre‐heating of hot water, solvents should exhibit high flash points above 100°C and low specific heat capacities. The release of stored energy at night or in colder climates also requires solvents with low melting points to avoid solidification and reduce insulation demands. For the integration of solution‐state MOST systems into photochemical and packed‐bed flow reactors [13, 14], solvent properties like dynamic viscosity and thermal conductivity are expected to influence pressure drop and heat transfer. The solvent should also be chemically compatible with materials used in larger‐scale device construction. Finally, in accordance with EU regulations, the solvent should ideally be environmentally benign and non‐toxic. Balancing these requirements presents a significant challenge in the development of practical MOST systems.

To date, MOST systems have been predominantly studied in organic solvents, most commonly toluene, which provides excellent solubility for NBD derivatives (up to 1.52 M) and a low specific heat capacity (1.71 J g^−1^ K^−1^) [15], enabling efficient heat release and significant temperature increases during energy discharge. MOST systems in solution are inherently limited in volumetric energy density due to dilution effects; however, toluene offers a suitable medium for both photoisomerization and back‐conversion. As a practical example, packed‐bed reactions have been investigated in a jacketed glass reactor under low‐vacuum conditions using toluene as the flow medium, demonstrating favorable flow characteristics, including enhanced mixing at higher flow rates, good permeability throughout the packed bed, and heat losses of approximately 30% under optimized reaction conditions [16]. However, its toxicity, volatility, and low flash point limit the suitability of toluene for sustainable and large‐scale applications, motivating the search for safer and greener alternatives.

**FIGURE 1:**
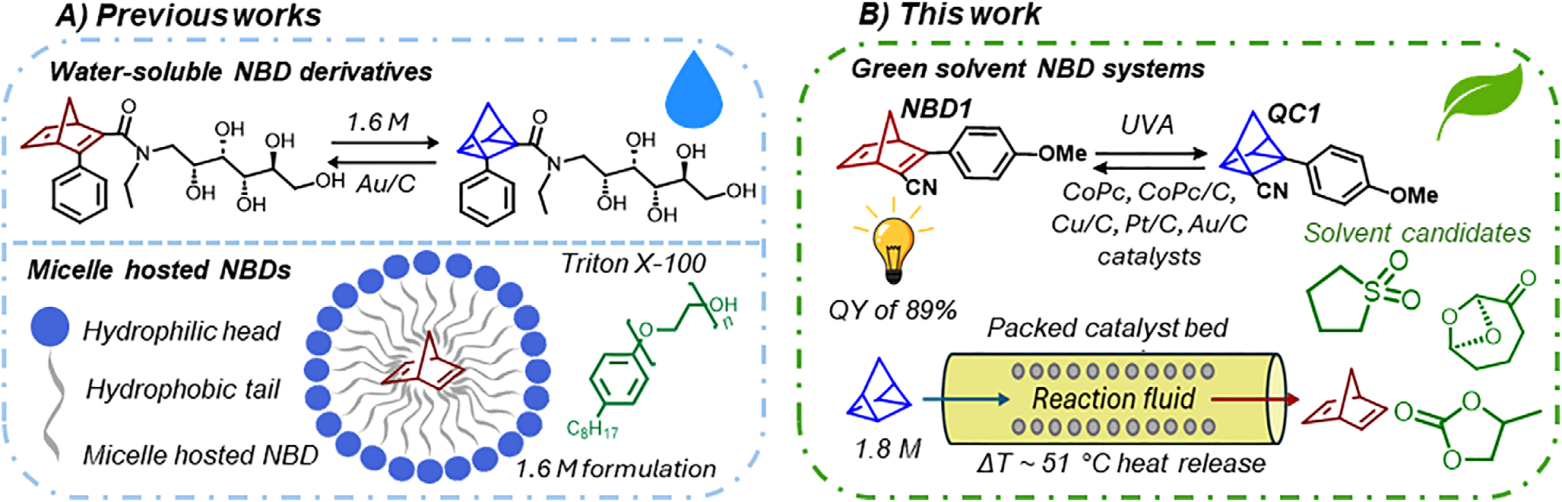
(A) Previous works on sustainable solvent‐based MOST systems, including a water‐soluble NBD derivative reaching up to 1.6 M concentration and a Triton‐X100 surfactant NBD derived phase mixture with concentrations up to 1.6 M. (B) Current work involving greener solvent‐based NBD systems.

Water has emerged as a potential green solvent and has been explored in several studies. Early work by Maruyama et al. demonstrated water‐soluble NBD systems and the catalytic back‐reaction using cobalt porphyrin complexes [17]. Subsequent work by Castro et al. investigated NBD‐QC derivatives in aqueous media, reporting long half‐lives and moderate energy storage densities [18, 19]. A push‐pull NBD design enabled red‐shifted absorption and improved aqueous solubility (up to 0.47 M in its anionic form) though this enhanced solubility is strongly pH‐dependent and accompanied by reduced stability, which limits practical applicability [20]. Sampedro, Funes‐Ardoiz et al. synthesized amide‐substituted NBD derivatives with absorption extending into the visible region (Figure 1A). These compounds exhibited high water solubilities up to 1.6 M, although their moderate energy densities generated temperature increases of up to 7.5°C in solution compared to 40°C in the solid state [21, 22]. Despite these promising results, thermal back‐conversion in aqueous systems required elevated starting temperatures above 100°C or the addition of colloidal gold nanoparticles to trigger the reversion process. In an alternative approach, surfactant‐assisted emulsions have been used to dissolve unmodified, already high‐performing hydrophobic NBD photoswitches in water, achieving concentrations of up to 1.6 M (Figure 1A) [23]. The metrics of these MOST systems have been summarized (see Table S7). A key challenge for water‐based MOST systems is the high specific heat capacity of water (4.18 J g^−^^1^ K^−^^1^) and water/surfactant mixtures. While advantageous for heat storage, this property reduces temperature increase during rapid energy release and makes large temperature increases more difficult to achieve compared with organic solvents. In addition, the boiling point of 100°C restricts operation to relatively low‐temperature regimes.

Consequently, the present work focuses on the exploration of more sustainable solvent alternatives that combine low specific heat capacities, low viscosities, good thermal conductivities, high flash points, biodegradability, and low toxicities (Figure 1B). The photoswitch selected for this study is 2‐cyano‐3‐(4‐methoxyphenyl)‐norbornadiene (**NBD1**), a high‐performing system that has been extensively studied and can be synthesized under flow conditions [24, 25]. **NBD1** was chosen for its near‐visible absorption (λ_onset_ ≈ 380 nm) and high energy density (0.4 MJ kg^−^^1^), and demonstrated cycling stability, with a decay of only 0.14% per cycle in toluene, corresponding to approximately 60% retention after 1 year of daily operation [11]. At present, solvent effects in NBD–QC systems have been investigated in a limited number of studies Moth‐Poulsen et al. reported extended thermal half‐lives in certain polar solvents, while Mikkelsen et al. demonstrated that increased solvent viscosity can enhance thermal stability [26]. Building on these observations, we systematically evaluate a broad range of organic solvents for the **NBD1** system, investigating both the photochemical NBD‐to‐QC conversion and the catalytic QC‐to‐NBD back‐reaction. From this screening, three greener solvent candidates were identified (cyrene, propylene carbonate, sulfolane) and studied in greater detail. The most promising solvent, propylene carbonate (Pc), was subsequently evaluated with a series of catalysts previously shown to promote the **QC1** to **NBD1** back‐reaction in toluene, including Pt/C, and earth‐abundant alternatives such as cobalt complexes and Cu/C [5, 11]. Finally, to assess the thermal output from a highly concentrated system, **QC1** was dissolved to a concentration of 1.79 M in Pc and subjected to catalytic back‐conversion in a custom‐built fixed‐bed catalytic reactor.

## 2 Results and Discussion

**FIGURE 2:**
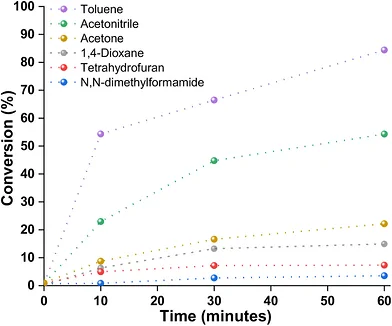
Screening of six commonly available solvents for the **QC1** to **NBD1** catalytic back‐reaction using a Pt/C catalyst. Conversion refers to the percentage of NBD recovered after the catalytic back‐conversion, determined from the relative absorbance at 340 nm.

Before moving to greener solvents, an initial screening was conducted to systematically probe the influence of solvent properties on both the NBD to QC photoisomerization and the catalytic back‐conversion. A representative set of standard organic solvents spanning multiple classes was selected, including polar aprotic, dipolar aprotic, non‐polar, and halogenated media. The solvents evaluated were dichloromethane (DCM), toluene, acetone, acetonitrile, 1,4‐dioxane, tetrahydrofuran (THF), and *N*,*N*‐dimethylformamide (DMF). Although several of these solvents possess properties that render them unsuitable for practical MOST deployment (e.g., low flash points or toxicity), their inclusion provides valuable mechanistic insight into solvent effects. For this initial screening, dilute solutions of **NBD1** (4.48×10^−3^ M) were used. Upon irradiation, complete photoisomerization from **NBD1** to **QC1** was observed in all solvents except DCM, and the absorption onset and maxima remained essentially unchanged across the solvent set (see Figures S1 and S2). The incomplete conversion observed in DCM is attributed to its known photo‐instability under UVA irradiation, which can lead to the production of hydrochloric acid and other reactive species [27, 28]. In the presence of oxygen, photooxidative degradation may occur, likely interfering with the **NBD1** to **QC1** conversion in aerated DCM [29]. For the remaining solvents, the catalytic back‐reaction from **QC1** to **NBD1** was initiated using a previously reported Pt/C catalyst known for its high activity in toluene‐based systems [4]. Reaction progress was monitored over time by UV‐Vis spectroscopy following established protocols [5], and the conversion percentage to **NBD1** was determined from the relative absorbance at 340 nm, where only **NBD1** absorbs, enabling comparative assessment of catalytic efficiency across solvent environments (Figure 2).

The catalytic back‐reaction from **QC1** to **NBD1** proceeded most efficiently in non‐polar and aprotic solvents, with toluene and acetonitrile, respectively, exhibiting the highest activities and rate constants on the order of 10^−2^ s^−1^. In contrast, more polar solvents such as acetone, 1,4‐dioxane, THF, and DMF exhibited markedly reduced catalytic activities, with rates several orders of magnitude lower (see Table S4). To gain further insight into the solvent‐catalyst interactions, the catalyst was characterized by X‐ray photoelectron spectroscopy (XPS), revealing a major PtO doublet peak and a minor PtO_2_ doublet peak consistent with Pt^2+^ and Pt^4+^, respectively. Both Pt species may contribute to the catalytic activity (see Figures S6–S8). While XPS does not directly probe solvent interactions, the presence of partially oxidized Pt sites and surface oxygen species suggests that solvents with strong hydrogen bonding ability interact with the catalyst surface, thereby impeding access of **QC1** species to active sites. This behavior is consistent with competitive coordination at Pt^2+^ active sites, which may reduce **QC1** binding and catalytic turnover. Toluene, being largely non‐coordinating, is unlikely to compete with **QC1** for access to active sites. Although acetonitrile is known to coordinate to Pt(II) [30], this interaction is relatively labile and can be displaced by **QC1,** thereby preventing significant catalyst poisoning. Additional correlations based on Hansen's solubility parameters [31, 32] and Gutmann's “donor‐acceptor” concept [33, 34] were also explored (see SI for additional details). The combined influence of solvent parameters, including low hydrogen‐bond donating ability, a high acceptor‐to‐donor number ratio, and weak coordination strength, governs the overall catalytic performance in the respective solvent. No further correlations were observed between catalytic activity and other physical solvent properties such as dielectric constant, viscosity, or boiling point.

These results provide a foundation for exploring more sustainable solvent candidates that are preferably non‐polar and aprotic. Based on these observations, three greener solvents were selected for further evaluation: cyrene (Cy) [35], sulfolane (Sul) [36], and propylene carbonate (Pc) [37]. Cy is a biodegradable dipolar aprotic solvent derived from biomass and has been proposed as a replacement for DMF in organic synthesis. Sul is widely used in extractive distillation; despite its low degradability, it is highly water miscible and has very low volatility. Pc, derived from carbon dioxide, is employed in lithium‐ion batteries to facilitate lithium‐ion intercalation into graphitized anodes and ranks among the highest across multiple criteria in GSK's solvent sustainability guide [38]. These solvents meet several of the physical criteria discussed above for MOST applications, including relatively low specific heat capacities (ranging between 1.5‐2 J g^−1^ K^−1^) and flash points exceeding 100°C (see Table S5). Both Pc and Sul exhibit higher Gutmann acceptor numbers (AN) than donor numbers (DN), with differences of 3.2 and 4.4, respectively, indicating a Lewis acidic character. The higher AN values reflect an increased ability to stabilize electron density, either from the catalyst surface or from polar transition states. Although AN/DN values are not reported for Cy, its similar chemical structure to Pc suggests it exhibits comparable Lewis acid character. The three solvents exhibit varying hydrogen bond acceptor strengths, with Sul being significantly stronger than Cy, and Pc showing the weakest ability (see Table S6). For comparative analysis, the photochemical and catalytic performances were evaluated by ultra‐high performance liquid chromatography (UPLC), using the same reaction conditions (concentration, volume, temperature, stirring) as in the preliminary solvent screening (see Figures S9–S24). For each reaction, three measurements were performed: prior to photoisomerization, after photoisomerization, and following the catalytic reaction. For each measurement, 20 µL of the reaction mixture was diluted in 1 mL of acetonitrile and filtered through a 0.45 µm PTFE filter prior to analysis. NBD solutions of 4.48 × 10^−3^ M were photoconverted to the QC state (2%–4% NBD species remaining in solution as confirmed by UPLC) and subsequently exposed to a catalyst (1 mg) for 1 h. The catalytic back‐conversion of **QC1** to **NBD1** varied significantly between solvents: 65% and 19% conversion were observed in Pc and Cy, respectively, whereas Sul exhibited minimal activity with less than 6% conversion. Sul was therefore excluded from further investigation.

The potential of Cy and Pc as solvents for MOST applications was further investigated by UV‐Vis spectroscopy. Cy exhibited an absorption onset at 406 nm, resulting in significant spectral overlap with the photoactive absorption region of **NBD1**. In contrast, Pc absorbs primarily in the UVB and UVC regions (UV cutoff ≈ 240 nm) and has a higher absorption onset near 324 nm, thereby avoiding overlap with the absorption of **NBD1** (see Figure S25). However, despite the overlap of Cy, a higher photoisomerization efficiency for 0.18 M solutions was exhibited in Cy, attaining full conversion after 2 h, compared to 70% in Pc. These results are shown in quantitative NMR experiments (see Figures S46–S49). This indicates that competitive light absorption by the solvent is not the defining factor governing the photoisomerization efficiency of **NBD1**. The quantum yield of **NBD1** photoisomerization in Pc was determined to be 89% compared to 64% in toluene (see Figures S42–S45) [9]. However, a reliable quantum‐yield measurement could not be obtained for Cy under the experimental conditions, as the strong absorption of the solvent resulted in detector saturation. These observations suggest that other solvent properties, such as viscosity, solvent reorganization dynamics, and specific solvent–photoswitch interactions, play a more significant role in determining the efficiency of the photoisomerization process than solvent spectral overlap alone. Next, the solubility of **NBD1** in both green solvents was assessed, wherein an average solubility of 0.93 M was determined for Cyrene, while a significantly higher solubility of 3.0 M was observed in Pc (see Tables S13 and S14). Given the substantially higher solubility of **NBD1** in Pc, this solvent was selected for subsequent larger‐scale experiments to investigate the heat release process of the MOST system. Prior to these experiments, the long‐term photostability of Pc under irradiation and thermal stability upon heating was evaluated. Pc was subjected to UVA irradiation and heating to 80°C for 24 h (see Figures S26–S36), and quantitative NMR analysis indicated negligible changes in the solvent under the experimental conditions investigated, demonstrating good photo‐ and thermal stability. The thermal back‐conversion kinetics of **QC1** in Pc were monitored by measuring the absorbance at 340 nm over time at five temperatures, ranging from 40°C to 80°C in intervals of 10°C (see Figures S50–S55), and the thermal half‐lives were extracted from Eyring analysis. Under ambient conditions (25°C), the **QC1** half‐life increased from 30 days in toluene to 45 days in Pc, highlighting enhanced suitability for monthly energy storage cycles.

**FIGURE 3:**
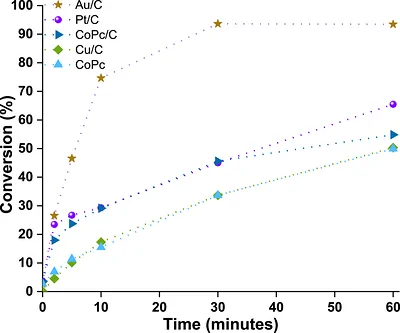
Catalytic back‐conversion of **QC1** to **NBD1** in propylene carbonate using five different catalysts. Conversion refers to the percentage of NBD recovered after the catalytic back‐conversion, determined from the relative absorbance at 340 nm.

To further investigate the catalytic **QC1** to **NBD1** reaction in Pc, batch experiments were conducted with the catalyst particles suspended in solution. A series of catalytic experiments using various catalysts was performed to assess their activity and selectivity (Figure 3). The metal loadings and relative molar percentages of each catalyst used are summarized in Table S10. Notably, the heterogeneous catalysts exhibited low molar loadings (1%–4%), significantly lower than those of the homogeneous cobalt phthalocyanine (CoPc) system. A commercially available Au catalyst supported on Vulcan XC‐72 exhibited the highest activity, likely due to its high metal loading (35 weight%) and narrow nanoscale particle size distribution (2‐3 nm). Two previously validated catalysts, an easily synthesisable Pt/C and active Cu/C [4], and a homogenous CoPc complex, demonstrated robust performance in Pc. Following a procedure similar to that reported by Wang et al. [11], a heterogeneous version of CoPc was prepared using Norit SX Plus activated carbon, the same support employed for Pt/C and Cu/C catalysts. A pronounced initial rate enhancement was observed for CoPc/C relative to homogeneous CoPc, resulting from the greater surface area and improved accessibility of the immobilized macrocycles [39]. This behavior reflects more effective utilization of active sites upon immobilization.

**FIGURE 4:**
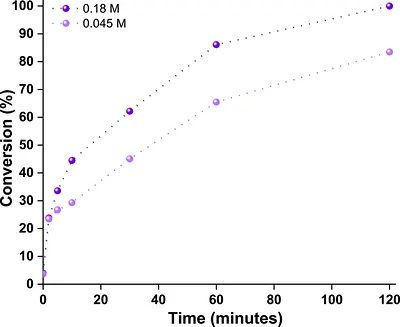
**QC1** to **NBD1** catalytic back‐reactions in 0.045 M and 0.18 M solutions of Pc.

The catalytically promoted **QC1** to **NBD1** back‐reaction proceeds rapidly in Pc. Using the same Pt/C catalyst, the reaction in Pc outperformed acetonitrile and showed reactivity comparable to that in toluene. Importantly, similar rates were observed across the range of catalysts, confirming the broad compatibility of Pc as a reaction medium. To benchmark solvent effects, catalytic reactions in Pc and toluene were directly compared (see Figures S56–S62). Catalytic rates for all five catalysts in Pc and toluene are summarized in Table S11. After 1 h, with the exception of the Au/C catalyst, which showed similar rates in both solvents, the catalysts exhibited rate constants of the same order of magnitude, being approximately three times slower than in toluene. Notably, the relative reduction in reaction rates among the catalysts was consistent between Pc and toluene, suggesting that solvent‐catalyst interactions are significant and preserved across media. Since the intrinsic reaction rates are similar in both solvents, the reduced conversion observed in Pc likely reflects its higher viscosity, which hinders mass transport of reactants to the active sites. In addition, toluene is non‐coordinating, whereas Pc can weakly coordinate with the catalytic sites, reducing effective turnover frequency. The catalytic activity at higher concentrations of **NBD1**/**QC1** was investigated using the Pt/C catalyst at a loading of 10 weight% relative to the amount of **QC1** reacted (Figure 4).

**FIGURE 5:**
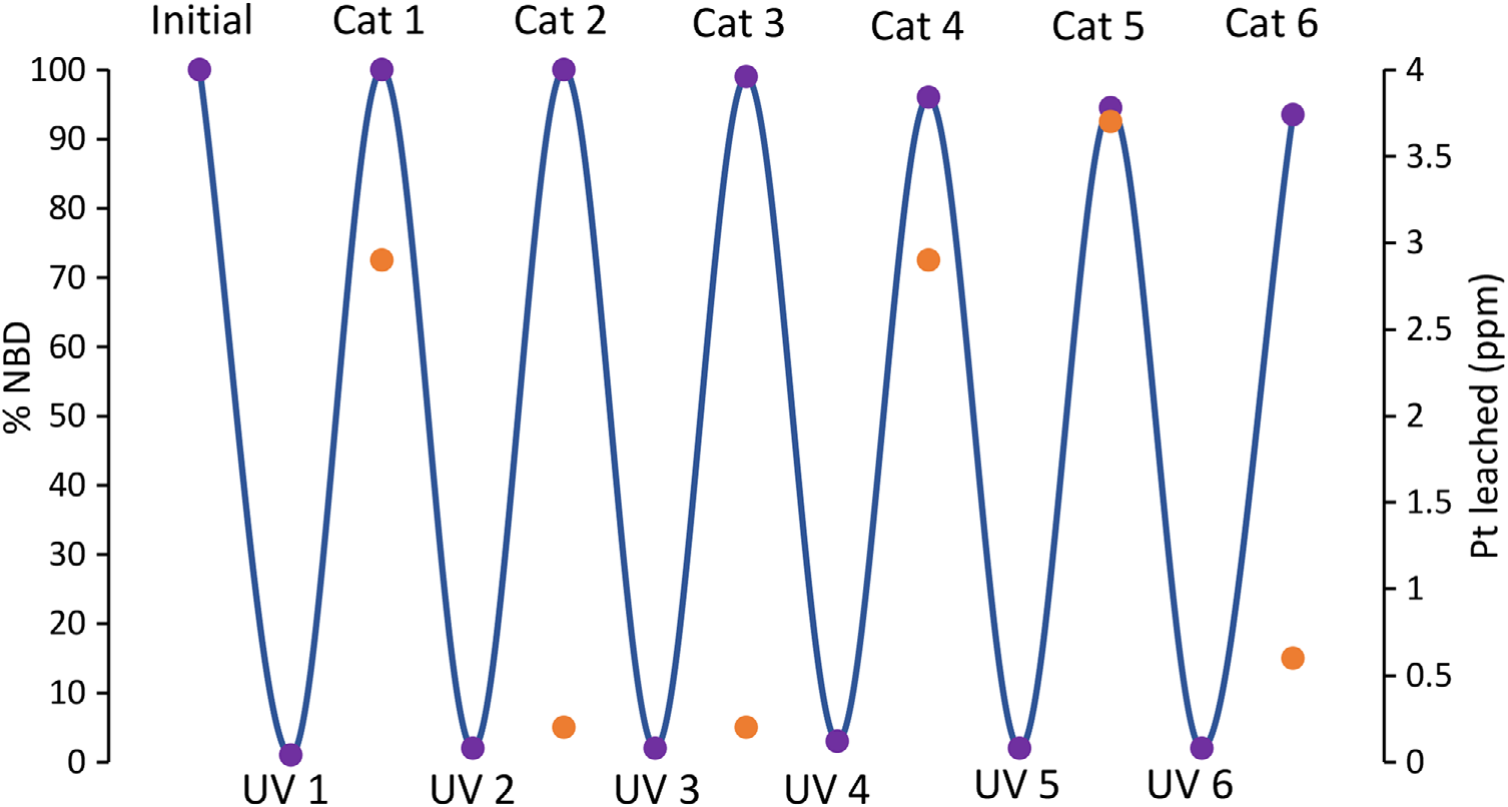
Cycling performance of the NBD/QC molecular photoswitch over six consecutive photoisomerization and catalytic back‐conversion cycles, monitored by ^1^H NMR spectroscopy (blue line) and platinum leaching from the Pt/C catalyst into Pc solution after repeated catalytic back‐conversion cycles, determined by X‐ray fluorescence analysis of the filtered reaction solution (orange circles).

The reaction rate increased from 1.51 × 10^−^^2^ s^−^^1^ to 2.15 × 10^−^^2^ s^−^^1^, corresponding to a factor increase of around 1.4, when the concentration was increased fourfold. This sub‐linear dependence suggests an intermediate regime between first and zero‐order kinetics, indicating that the catalyst surface is becoming increasingly saturated, limited by the availability of active sites [40]. Next, the cycling stability of the photoisomerization and catalytic reactions was investigated over six consecutive cycles at a substrate concentration of 0.18 M. The reaction efficiencies are shown by the blue line in Figure 5. The photoisomerization reaction remained highly effective over the six cycles, demonstrating good stability and reproducibility upon repeated cycling. The catalytic reactions after 6 h reached near‐completion during the initial cycles, with the conversion decreasing to 86% by the final cycle. The evolution of metal leaching from the catalyst support is also shown by the orange data points. A minor degree of platinum leaching, totaling approximately 10 ppm over the course of the cycling experiments, was observed. This suggests that the minor decrease in catalytic performance is primarily associated with the minor loss of active platinum during repeated cycling more than deactivation of active sites, indicating a reasonable degree of catalyst stability. This was further confirmed by measuring the metal concentration of the remaining solid catalyst used in the reaction before the first catalytic reaction and after the final cycle. The concentration of metal changed from 2.42% to 1.99%, see Supporting Information, equivalent to approximately 17.6% of original platinum (0.067 mg from 0.244 mg Pt) leached from the support into the solution, reflecting a small absolute quantity of metal despite the higher relative percentage change.

**FIGURE 6:**
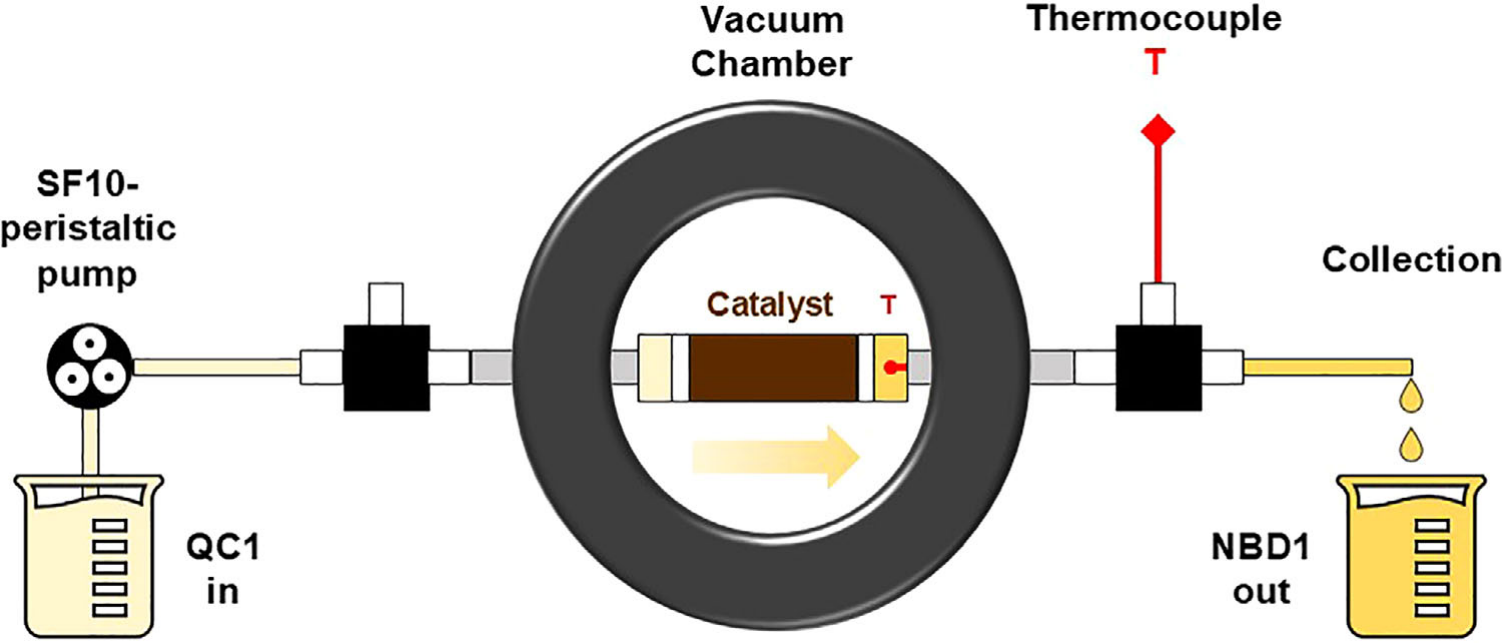
Schematic depiction of a built vacuum chamber with a catalyst bed to monitor the outlet temperature increase resulting from the catalytic **QC1** to **NBD1** back‐conversion.

After confirming photoconversion of **NBD1** and catalytic back conversion of **QC1** in Pc, we examined the solubility of **NBD1**/**QC1** in Pc. Solubility measurements of **NBD1** in Pc performed five times independently (see Table S13) yielded an average solubility of 3.0 M, exceeding the 1.52 M previously reported solubility of **NBD1** in toluene [11]. Using this concentration, the theoretical maximum thermal output of the MOST system was calculated at 87 °C, assuming no thermal losses (see Equation S5). The specific heat capacity of Pc (1.64 J g^−1^ K^−1^) is comparable to that of toluene (1.71 J g^−1^ K^−1^) [41, 42]. However, like many greener solvents, Pc has a higher volumetric mass density than toluene, reducing the predicted temperature increase to 61 °C at 1.79 M **NBD1**. The thermal response associated with the catalytic back‐conversion and heat‐release process was investigated experimentally using a custom‐built heat release setup chamber with inlet and outlet channels for controlled flow of the reaction mixture and a window for visual monitoring, similar to a previous setup [13]. The **QC1** solution was delivered to the catalytic reactor via a peristaltic pump with controlled flow rates. A turbopump maintained pressures around the 10^−4^ ‐ 10^−5^ mbar range, ensuring thermal isolation. A thermocouple (T) was positioned immediately downstream after the catalyst bed to monitor the temperature rise (ΔT) during the **QC1** to **NBD1** reaction (Figure 6).

The **NBD1** sample was first photoisomerized to **QC1** under irradiation in flow, and the isomeric ratio was determined by ^1^H NMR spectroscopy. While photoisomerization in Pc at a concentration of 1.8 M only resulted in 70% conversion to **QC1** under these reaction conditions, photoisomerization in toluene at this concentration resulted in quantitative conversion (see Figures S66 and S67). Although Pc exhibited a higher photoisomerization quantum yield under dilute conditions, complete conversion was not achieved under the concentrated flow irradiation conditions used for the heat‐release experiments. Under these conditions (1.8 M), the overall photoconversion is influenced not only by the intrinsic quantum yield but also by reactor design, residence time, optical path length, and light penetration through the highly absorbing solution. Furthermore, at higher concentrations, absorbance of the photoisomer, in this case **QC1**, becomes significant, thus possible back‐conversion under irradiation and/or the reaching of a photostationary state need to be considered. Although higher concentrations would be possible due to the solubility, we kept the concentration at 1.8 M for practical reasons to ensure the fluidity inside the system, due to viscosity playing a crucial role and to try to avoid pressure differences.

**FIGURE 7:**
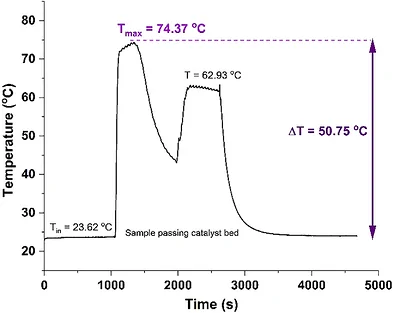
The heat release for the back‐conversion of **QC1** to **NBD1** with Pt/C catalyst, measured using the setup shown in Figure 6.

The **QC1** solution was then passed through the Pt/C catalytic reactor bed at a flow rate of 5 mL h^−1^. The heat release experiment resulted in a temperature increase of 51°C, with the outlet temperature increasing from 23°C to 74°C (Figure 7). Transient fluctuations occurred due to pressure build‐up and air bubbles in the tubing, causing a temporary temperature drop. After pressure stabilization, the temperature rose again to approximately 63°C.

Back‐conversion of up to 71.4% of the 1.8 M **QC1** solution was confirmed by ^1^H NMR spectroscopy (Figures S70 and S71), corresponding to an effective conversion of approximately 1.29 M **QC1** to **NBD1**. The predicted temperature increase of 47°C represents an ideal first‐order estimate assuming adiabatic conditions, homogeneous conversion, and constant heat capacity. Heat losses to the surroundings are therefore neglected in this estimation. This first‐order estimation neglects heat losses to the surroundings and is intended to provide an idealized comparison with the experimentally observed outlet temperature increase. Similar adiabatic assumptions have recently been employed in thermal modeling studies of related MOST systems [43, 44]. The higher experimentally observed temperature increase may arise from localized reaction zones within the catalytic bed, transient heat release, and the temperature dependence of the **NBD1**/**QC1** mixture's heat capacity. Previous studies have shown that concentration variance at the fluid‐catalyst interface results from the interplay between the reaction constant and the degree of mixing [13]. When the reaction rate is fast relative to mixing, concentration gradients persist longer as variance decays more as the fluid flows through the reactor. Larger concentration gradients are expected at higher concentrations, although this is mitigated by the fact that the catalytic reaction constant increased when quadrupling the concentration. Nevertheless, the measured temperature increase remains below the theoretical maximum of 61°C for complete conversion of a 1.8 M solution, indicating that longer residence times would be needed to achieve full conversion. The analysis revealed only minor changes in the surface oxidation‐state distribution after the reaction, with a slight increase in the contribution assigned to Pt(IV) and a corresponding decrease in Pt(II), while the overall surface Pt content remained comparable before and after reaction (see Figures S77–S82). These results demonstrate that the NBD–QC MOST system can demonstrate the capability for low‐grade heat generation under thermally isolated conditions, supporting its feasibility for integration into future heating technologies.

## 3 Conclusions

A systematic solvent screening identified propylene carbonate (Pc) as a particularly promising medium for NBD–QC MOST systems. Among the greener aprotic solvents evaluated, Pc enables high solubility of **NBD1**, efficient **NBD1** to **QC1** photoisomerization, and thermal storage lifetimes of **QC1** compatible with monthly cycling, while also maintaining compatibility with a broad range of catalysts for the **QC1** to **NBD1** back‐conversion and heat release. Although catalytic rates are somewhat reduced in Pc relative to toluene, Pc still facilitates effective **QC1** to **NBD1** conversion and associated heat release, yielding operating temperatures up to 74°C, suitable for space and water heating purposes, even when accounting for realistic thermal losses. Future studies should prioritize integrating MOST operation into jacketed reactors and heat‐exchange systems for in‐loop water heating, alongside further optimization of photochemical flow conditions at higher concentrations. Overall, these results establish Pc as a viable and more sustainable alternative to conventional organic solvents, demonstrating that both efficient photoisomerization and catalytic back‐conversion of this NBD/QC MOST system can be achieved within a single, practically relevant solvent environment. This work represents a significant step toward implementing sustainable MOST systems for practical solar thermal energy storage applications.

## Author Contributions

**Lucien Magson**: conceptualization, investigation, writing – original draft, methodology, writing – review and editing, formal analysis, data curation. **Helen Hölzel**: investigation, data curation, methodology, writing – review and editing. **Rebecca J. Salthouse**: investigation, methodology, data curation, writing – review and editing. **Liang Fei**: investigation. **Pedro Ferreira**: investigation. **Vitor A. S. Almodovar**: investigation. **Kasper Moth‐Poulsen**: funding acquisition, writing – review and editing, supervision, resources, project administration. **Ignacio Funes‐Ardoiz**: writing – review and editing, funding acquisition, supervision. **Diego Sampedro**: conceptualization, funding acquisition, writing – review and editing, project administration, supervision, resources.

## Conflicts of Interest

The authors declare no conflicts of interest.

## Supporting information




**Supporting File**: advs77637‐sup‐0001‐SuppMat.docx.

## Data Availability

The data that support the findings of this study are available from the corresponding author upon reasonable request.
