# Supplementary material for: Greener Solvents for the Photoisomerization and Catalytic Heat Release of a Norbornadiene‐Based Molecular Solar Thermal Energy Storage System
Source: Adv Sci (Weinh). 2026 Sep 11:e77637. Online ahead of print. doi: 10.1002/advs.77637 (PMC13564281; doi:10.1002/advs.77637)
Supplement: Supplementary file 1 [file ADVS-9999-e77637-s001.docx]

Supporting Information

Greener Solvents for the Photoisomerisation and Catalytic Heat Release of a Norbornadiene-based Molecular Solar Thermal Energy Storage System

L. Magson, H. Hölzel, R. J. Salthouse, L. Fei, P. Ferreira, V. A. S. Almodovar, K. Moth-Poulsen, I. Funes-Ardoiz* and D. Sampedro*

L. Magson, Dr. I. Funes-Ardoiz and Prof. D. Sampedro
Instituto de Investigación Química de la Universidad de La Rioja (IQUR), Universidad de La Rioja, C/Madre de Dios 53, 26004 Logroño, La Rioja

[ignacio.funesa@unirioja.es](mailto:ignacio.funesa@unirioja.es); [diego.sampedro@unirioja.es](mailto:diego.sampedro@unirioja.es)

Dr. H. Hölzel

Institute of Organic Chemistry, Justus-Liebig-University Giessen, Heinrich-Buff-Ring 17, 35392 Giessen, Germany

Dr. H. Hölzel, Dr. R. J. Salthouse, Dr. L. Fei, Dr. P. Ferreira, Dr. V. A. S. Almodovar, and Prof. K. Moth-Poulsen

Department of Chemical Engineering, Universitat Politècnica de Catalunya, EEBE, Eduard Maristany 10-14, 08019 Barcelona, Spain.

Prof. K. Moth-Poulsen

Department of Chemistry and Chemical Engineering, Chalmers University of Technology, Kemivagen 4, Gothenburg 412 96, Sweden.

Department of Chemistry and Chemical Engineering, Chalmers University of Technology, Catalan Institution for Research & Advanced Studies, ICREA, Pg. Llu-íıs Companys 23, Barcelona, Spain.

Institute of Materials Science of Barcelona, ICMAB-CSIC, Bellaterra, Barcelona, 08193, Spain.

**Table of Contents**

[1	Preliminary screening	3](#_Toc236407664)

[Photoisomerisation from NBD1 to QC1	3](#_Toc236407665)

[Hansen Solubility Parameters (HSP)	5](#_Toc236407666)

[Gutmann-Beckett method	6](#_Toc236407667)

[Catalytic rates in different solvents	8](#_Toc236407668)

[X-ray photoelectron spectroscopy (XPS)	9](#_Toc236407669)

[2	Green solvent screening	11](#_Toc236407670)

[Physical and chemical properties of green solvents	11](#_Toc236407671)

[Isomerisation reactions monitored by UPLC	12](#_Toc236407672)

[UV-Vis spectra	21](#_Toc236407673)

[3	Propylene Carbonate	22](#_Toc236407674)

[Photostability	22](#_Toc236407675)

[Thermal stability	24](#_Toc236407676)

[Calibration plots of NBD1 in propylene carbonate and toluene	27](#_Toc236407677)

[Potassium ferrioxalate actinometry	28](#_Toc236407678)

[Preparation of the actinometry solution	29](#_Toc236407679)

[Photon flux measurement	29](#_Toc236407680)

[Photoisomerisation Quantum Yield	30](#_Toc236407681)

[Kinetics of the thermal QC1 to NBD1 back-reaction	37](#_Toc236407682)

[Solubility of NBD in Propylene Carbonate	46](#_Toc236407683)

[Photoconversion of NBDs under flow conditions	47](#_Toc236407684)

[Heat release experiment using Pt/C catalytic bed in Propylene carbonate	49](#_Toc236407685)

[Macroscopic Heat Release	51](#_Toc236407686)

[Cycling experiments	52](#_Toc236407687)

[NMR analysis	52](#_Toc236407688)

[X-ray fluorescence (XRF)	53](#_Toc236407689)

[ICP-OES	54](#_Toc236407690)

[X-ray photoelectron spectroscopy (XPS)	56](#_Toc236407691)

[4	References	62](#_Toc236407692)

# 1 Preliminary screening

## Photoisomerisation from NBD1 to QC1

A LuzChem photoreactor LZC4 containing 15 UVA lamps of 8 Watts was used to irradiate the NBD compound. All solutions were measured using a UV-3600 Shimadzu UV-Vis NIR spectrometer.

Absorbance (a.u)

| Wavelength (nm) | THF | 1,4-dioxane | DMF | Acetonitrile |
| --- | --- | --- | --- | --- |
| 290 | 0.28019 | 0.31368 | 0.34578 | 0.35552 |
| 290.5 | 0.28162 | 0.3197 | 0.35268 | 0.35422 |
| 291 | 0.29156 | 0.31571 | 0.35828 | 0.35272 |
| 291.5 | 0.2864 | 0.32232 | 0.36546 | 0.35052 |
| 292 | 0.29689 | 0.31798 | 0.37525 | 0.3531 |
| 292.5 | 0.30224 | 0.3256 | 0.36883 | 0.35719 |
| 293 | 0.29763 | 0.33268 | 0.37482 | 0.35663 |
| 293.5 | 0.30767 | 0.33772 | 0.37526 | 0.35668 |
| 294 | 0.31535 | 0.34051 | 0.37867 | 0.36272 |
| 294.5 | 0.31201 | 0.3441 | 0.38833 | 0.36403 |
| 295 | 0.32536 | 0.35034 | 0.38412 | 0.36381 |
| 295.5 | 0.33012 | 0.35577 | 0.38673 | 0.36069 |
| 296 | 0.32927 | 0.36167 | 0.3933 | 0.36541 |
| 296.5 | 0.34107 | 0.36712 | 0.39606 | 0.37019 |
| 297 | 0.34143 | 0.37191 | 0.39917 | 0.36821 |
| 297.5 | 0.35447 | 0.37876 | 0.4004 | 0.37385 |
| 298 | 0.35441 | 0.38769 | 0.4088 | 0.38375 |
| 298.5 | 0.36604 | 0.38946 | 0.40787 | 0.37653 |
| 299 | 0.36649 | 0.39705 | 0.41309 | 0.386 |
| 299.5 | 0.37477 | 0.40129 | 0.41709 | 0.38859 |
| 300 | 0.38343 | 0.4093 | 0.41818 | 0.38644 |
| 300.5 | 0.38549 | 0.41373 | 0.42555 | 0.39388 |
| 301 | 0.39929 | 0.4233 | 0.43209 | 0.39615 |
| 301.5 | 0.39947 | 0.42854 | 0.43146 | 0.40398 |
| 302 | 0.41658 | 0.43315 | 0.43924 | 0.4038 |
| 302.5 | 0.41919 | 0.44004 | 0.44659 | 0.4043 |
| 303 | 0.42318 | 0.45056 | 0.44929 | 0.40547 |
| 303.5 | 0.42647 | 0.456 | 0.46043 | 0.41325 |
| 304 | 0.43502 | 0.46538 | 0.46146 | 0.41804 |
| 304.5 | 0.43914 | 0.47102 | 0.47135 | 0.41839 |
| 305 | 0.45235 | 0.47751 | 0.47847 | 0.42455 |
| 305.5 | 0.45088 | 0.48671 | 0.47928 | 0.43081 |
| 306 | 0.46171 | 0.49434 | 0.47995 | 0.42934 |
| 306.5 | 0.46683 | 0.50242 | 0.48937 | 0.43336 |
| 307 | 0.47775 | 0.5063 | 0.49186 | 0.44094 |
| 307.5 | 0.4807 | 0.51495 | 0.50239 | 0.4393 |
| 308 | 0.48611 | 0.52218 | 0.50978 | 0.44688 |
| 308.5 | 0.49379 | 0.52685 | 0.5101 | 0.44716 |
| 309 | 0.49589 | 0.53449 | 0.51546 | 0.45118 |
| 309.5 | 0.50497 | 0.53664 | 0.52217 | 0.45485 |
| 310 | 0.50993 | 0.54843 | 0.52291 | 0.4581 |
| 310.5 | 0.51129 | 0.54993 | 0.53189 | 0.46056 |
| 311 | 0.51873 | 0.55208 | 0.53404 | 0.4615 |
| 311.5 | 0.51708 | 0.55899 | 0.53995 | 0.47044 |
| 312 | 0.52536 | 0.56059 | 0.54791 | 0.46781 |
| 312.5 | 0.52957 | 0.57607 | 0.5533 | 0.47346 |
| 313 | 0.53504 | 0.57466 | 0.55276 | 0.47011 |
| 313.5 | 0.54357 | 0.58073 | 0.564 | 0.47392 |
| 314 | 0.54693 | 0.59085 | 0.56463 | 0.48066 |
| 314.5 | 0.54751 | 0.59564 | 0.57239 | 0.48673 |
| 315 | 0.55494 | 0.59635 | 0.5792 | 0.49165 |
| 315.5 | 0.56282 | 0.59947 | 0.58443 | 0.4914 |
| 316 | 0.56878 | 0.61058 | 0.59264 | 0.49365 |
| 316.5 | 0.57528 | 0.62254 | 0.59425 | 0.50364 |
| 317 | 0.57535 | 0.61747 | 0.60202 | 0.50519 |
| 317.5 | 0.58815 | 0.63072 | 0.60801 | 0.50797 |
| 318 | 0.58391 | 0.63524 | 0.61392 | 0.5072 |
| 318.5 | 0.58719 | 0.63883 | 0.61797 | 0.51067 |
| 319 | 0.59367 | 0.63799 | 0.62312 | 0.51489 |
| 319.5 | 0.5997 | 0.64565 | 0.63184 | 0.51922 |
| 320 | 0.59995 | 0.65018 | 0.63391 | 0.51837 |
| 320.5 | 0.6072 | 0.64928 | 0.63729 | 0.51803 |
| 321 | 0.60814 | 0.65735 | 0.6367 | 0.52134 |
| 321.5 | 0.6103 | 0.6617 | 0.64728 | 0.52042 |
| 322 | 0.61553 | 0.66426 | 0.64587 | 0.52483 |
| 322.5 | 0.61 | 0.66347 | 0.64993 | 0.51916 |
| 323 | 0.61836 | 0.66578 | 0.65451 | 0.52779 |
| 323.5 | 0.61406 | 0.66474 | 0.65742 | 0.52427 |
| 324 | 0.61906 | 0.66352 | 0.6616 | 0.52442 |
| 324.5 | 0.61596 | 0.66552 | 0.66063 | 0.52409 |
| 325 | 0.62006 | 0.6697 | 0.66167 | 0.52368 |
| 325.5 | 0.61909 | 0.66865 | 0.66587 | 0.52184 |
| 326 | 0.61714 | 0.66552 | 0.66533 | 0.51955 |
| 326.5 | 0.61905 | 0.66367 | 0.66848 | 0.51899 |
| 327 | 0.6126 | 0.66483 | 0.66554 | 0.51305 |
| 327.5 | 0.61164 | 0.66457 | 0.66403 | 0.51464 |
| 328 | 0.6083 | 0.65977 | 0.66582 | 0.51144 |
| 328.5 | 0.60483 | 0.65682 | 0.66354 | 0.50813 |
| 329 | 0.60462 | 0.65428 | 0.6614 | 0.5057 |
| 329.5 | 0.60284 | 0.65372 | 0.65941 | 0.50146 |
| 330 | 0.59789 | 0.64838 | 0.65942 | 0.49901 |
| 330.5 | 0.58939 | 0.64017 | 0.65458 | 0.49435 |
| 331 | 0.58992 | 0.63704 | 0.65148 | 0.4897 |
| 331.5 | 0.5825 | 0.63173 | 0.64618 | 0.48369 |
| 332 | 0.57752 | 0.63228 | 0.64644 | 0.48113 |
| 332.5 | 0.5727 | 0.6216 | 0.64474 | 0.47337 |
| 333 | 0.56892 | 0.6167 | 0.63649 | 0.46756 |
| 333.5 | 0.56334 | 0.60964 | 0.63222 | 0.46656 |
| 334 | 0.556 | 0.60221 | 0.62827 | 0.46024 |
| 334.5 | 0.54829 | 0.59087 | 0.62166 | 0.45573 |
| 335 | 0.54124 | 0.58909 | 0.61599 | 0.44685 |
| 335.5 | 0.53352 | 0.58231 | 0.61239 | 0.44234 |
| 336 | 0.52953 | 0.57318 | 0.60272 | 0.43128 |
| 336.5 | 0.51922 | 0.56566 | 0.59984 | 0.42415 |
| 337 | 0.50854 | 0.55533 | 0.58756 | 0.41768 |
| 337.5 | 0.49888 | 0.54342 | 0.58093 | 0.40987 |
| 338 | 0.48999 | 0.53615 | 0.57391 | 0.40264 |
| 338.5 | 0.48136 | 0.52669 | 0.56644 | 0.39356 |
| 339 | 0.47073 | 0.51428 | 0.5606 | 0.38923 |
| 339.5 | 0.46305 | 0.50934 | 0.55093 | 0.38066 |
| 340 | 0.45357 | 0.49695 | 0.54311 | 0.37441 |
| 340.5 | 0.44198 | 0.48635 | 0.5303 | 0.364 |
| 341 | 0.43398 | 0.47688 | 0.52516 | 0.35758 |
| 341.5 | 0.42494 | 0.46624 | 0.51432 | 0.34837 |
| 342 | 0.41542 | 0.45717 | 0.50447 | 0.34117 |
| 342.5 | 0.40607 | 0.44692 | 0.49565 | 0.33208 |
| 343 | 0.39602 | 0.43505 | 0.48657 | 0.32548 |
| 343.5 | 0.3857 | 0.42484 | 0.47627 | 0.31358 |
| 344 | 0.37522 | 0.41557 | 0.46418 | 0.30662 |
| 344.5 | 0.36067 | 0.40043 | 0.45731 | 0.29763 |
| 345 | 0.35454 | 0.39034 | 0.44334 | 0.2851 |
| 345.5 | 0.33992 | 0.37876 | 0.43378 | 0.27772 |
| 346 | 0.33411 | 0.3661 | 0.42504 | 0.2702 |
| 346.5 | 0.31954 | 0.3529 | 0.41433 | 0.25987 |
| 347 | 0.30974 | 0.34573 | 0.39913 | 0.25413 |
| 347.5 | 0.30024 | 0.33101 | 0.38727 | 0.24304 |
| 348 | 0.29217 | 0.32623 | 0.38155 | 0.23724 |
| 348.5 | 0.2789 | 0.30868 | 0.36522 | 0.22724 |
| 349 | 0.26983 | 0.30314 | 0.35934 | 0.22122 |
| 349.5 | 0.25497 | 0.28831 | 0.35002 | 0.21075 |
| 350 | 0.2501 | 0.27745 | 0.33587 | 0.20213 |
| 350.5 | 0.24022 | 0.26685 | 0.32655 | 0.19567 |
| 351 | 0.23025 | 0.26073 | 0.31648 | 0.18819 |
| 351.5 | 0.21948 | 0.24618 | 0.30457 | 0.17918 |
| 352 | 0.2107 | 0.23593 | 0.29269 | 0.17504 |
| 352.5 | 0.20023 | 0.22824 | 0.28375 | 0.16616 |
| 353 | 0.18975 | 0.21708 | 0.27248 | 0.1577 |
| 353.5 | 0.18142 | 0.20751 | 0.26291 | 0.14939 |
| 354 | 0.17338 | 0.19783 | 0.2528 | 0.14226 |
| 354.5 | 0.16582 | 0.18907 | 0.24468 | 0.13806 |
| 355 | 0.15634 | 0.17929 | 0.23147 | 0.1306 |
| 355.5 | 0.15034 | 0.16932 | 0.22565 | 0.12563 |
| 356 | 0.14082 | 0.16149 | 0.21316 | 0.11759 |
| 356.5 | 0.13512 | 0.15327 | 0.20501 | 0.11204 |
| 357 | 0.12433 | 0.14348 | 0.19286 | 0.10562 |
| 357.5 | 0.11746 | 0.13779 | 0.18405 | 0.09942 |
| 358 | 0.11083 | 0.12876 | 0.17711 | 0.0934 |
| 358.5 | 0.10268 | 0.12056 | 0.16704 | 0.08952 |
| 359 | 0.09837 | 0.11468 | 0.15788 | 0.08222 |
| 359.5 | 0.09106 | 0.10668 | 0.15129 | 0.07875 |
| 360 | 0.08645 | 0.10106 | 0.14219 | 0.07404 |
| 360.5 | 0.08024 | 0.09358 | 0.13331 | 0.0688 |
| 361 | 0.07313 | 0.08672 | 0.12861 | 0.06397 |
| 361.5 | 0.06776 | 0.08071 | 0.12227 | 0.05976 |
| 362 | 0.0619 | 0.07431 | 0.11443 | 0.05859 |
| 362.5 | 0.05936 | 0.07057 | 0.1065 | 0.05173 |
| 363 | 0.05396 | 0.06626 | 0.0986 | 0.04666 |
| 363.5 | 0.05148 | 0.06044 | 0.09294 | 0.04581 |
| 364 | 0.04714 | 0.05406 | 0.08717 | 0.04287 |
| 364.5 | 0.04473 | 0.05055 | 0.08233 | 0.0387 |
| 365 | 0.03971 | 0.04882 | 0.0772 | 0.03793 |
| 365.5 | 0.03455 | 0.04471 | 0.07057 | 0.03272 |
| 366 | 0.03365 | 0.03968 | 0.06559 | 0.02906 |
| 366.5 | 0.03102 | 0.03733 | 0.06187 | 0.02884 |
| 367 | 0.02777 | 0.03398 | 0.05565 | 0.02763 |
| 367.5 | 0.02432 | 0.03292 | 0.05056 | 0.02238 |
| 368 | 0.02318 | 0.02878 | 0.04986 | 0.02178 |
| 368.5 | 0.02047 | 0.0253 | 0.04426 | 0.01918 |
| 369 | 0.02013 | 0.02546 | 0.04016 | 0.01828 |
| 369.5 | 0.01751 | 0.02009 | 0.03526 | 0.0177 |
| 370 | 0.0166 | 0.02138 | 0.03373 | 0.01639 |
| 370.5 | 0.01458 | 0.0182 | 0.02987 | 0.01413 |
| 371 | 0.01336 | 0.01584 | 0.02645 | 0.01221 |
| 371.5 | 0.0114 | 0.01542 | 0.02504 | 0.01045 |
| 372 | 0.01141 | 0.01394 | 0.02429 | 0.00974 |
| 372.5 | 0.00935 | 0.01326 | 0.02038 | 0.00837 |
| 373 | 0.00761 | 0.00976 | 0.0174 | 0.00825 |
| 373.5 | 0.00626 | 0.00782 | 0.01633 | 0.00762 |
| 374 | 0.00624 | 0.00931 | 0.01419 | 0.0069 |
| 374.5 | 0.00454 | 0.00641 | 0.01225 | 0.00424 |
| 375 | 0.0063 | 0.00763 | 0.00928 | 0.00502 |
| 375.5 | 0.00429 | 0.00472 | 0.00799 | 0.00232 |
| 376 | 0.00397 | 0.00202 | 0.00899 | 0.00374 |
| 376.5 | 0.00346 | 0.00335 | 0.00726 | 0.0015 |
| 377 | 0.00354 | 0.00239 | 0.00517 | 0.00236 |
| 377.5 | 0.00425 | 0.00225 | 0.00386 | 0.0034 |
| 378 | 0.00202 | 0.00193 | 0.00295 | -0.0006 |
| 378.5 | 0.00187 | 0.00273 | 0.00221 | 0.00053 |
| 379 | 0.00098 | 0.00045 | 0.00012 | 0.00155 |
| 379.5 | 0.00093 | 0.00062 | -0.00119 | 0.00076 |
| 380 | 0 | 0 | 0 | 0 |
| 380.5 | 0.00156 | -6e-05 | -0.00064 | -0.00043 |
| 381 | 0.00033 | -0.00059 | -0.00133 | 1e-05 |
| 381.5 | 1e-05 | 0.00059 | -0.00375 | -0.00017 |
| 382 | -0.00018 | -0.00207 | -0.00255 | -0.00067 |
| 382.5 | 0.00146 | -0.00123 | -0.00389 | -0.00273 |
| 383 | -0.00019 | -0.00161 | -0.00437 | -0.00308 |
| 383.5 | 0.00106 | -0.00124 | -0.00453 | -0.00302 |
| 384 | 0.00126 | -0.0003 | -0.0025 | -0.0022 |
| 384.5 | 0.00059 | -0.00168 | -0.00482 | -0.00205 |
| 385 | 0.00112 | -0.00026 | -0.00566 | -0.0033 |
| 385.5 | 0.00064 | -0.00114 | -0.00666 | -0.00281 |
| 386 | 0.00044 | -0.00318 | -0.00453 | -0.00362 |
| 386.5 | 6e-05 | -0.00295 | -0.00691 | -0.00144 |
| 387 | 0.00086 | -0.00277 | -0.00743 | -0.0018 |
| 387.5 | 0.00105 | -0.00454 | -0.00644 | -0.0021 |
| 388 | 0.00063 | -0.00431 | -0.00694 | -0.00292 |
| 388.5 | 0.00053 | -0.00282 | -0.00629 | -0.00345 |
| 389 | 5e-05 | -0.00345 | -0.0085 | -0.00316 |
| 389.5 | 0.00065 | -0.00268 | -0.0062 | -0.00269 |

| Wavelength (nm) | Acetone |
| --- | --- |
| 323 | 0.32921 |
| 323.5 | 0.42961 |
| 324 | 0.45282 |
| 324.5 | 0.47362 |
| 325 | 0.44337 |
| 325.5 | 0.45336 |
| 326 | 0.43935 |
| 326.5 | 0.47322 |
| 327 | 0.45473 |
| 327.5 | 0.47176 |
| 328 | 0.46243 |
| 328.5 | 0.45526 |
| 329 | 0.46197 |
| 329.5 | 0.45952 |
| 330 | 0.45223 |
| 330.5 | 0.44768 |
| 331 | 0.44688 |
| 331.5 | 0.44198 |
| 332 | 0.44011 |
| 332.5 | 0.43062 |
| 333 | 0.42701 |
| 333.5 | 0.42737 |
| 334 | 0.41735 |
| 334.5 | 0.41573 |
| 335 | 0.40644 |
| 335.5 | 0.4024 |
| 336 | 0.39622 |
| 336.5 | 0.38887 |
| 337 | 0.38361 |
| 337.5 | 0.37698 |
| 338 | 0.36924 |
| 338.5 | 0.36146 |
| 339 | 0.35453 |
| 339.5 | 0.34806 |
| 340 | 0.34183 |
| 340.5 | 0.33247 |
| 341 | 0.32773 |
| 341.5 | 0.31864 |
| 342 | 0.31258 |
| 342.5 | 0.30304 |
| 343 | 0.29687 |
| 343.5 | 0.2884 |
| 344 | 0.28201 |
| 344.5 | 0.27055 |
| 345 | 0.2653 |
| 345.5 | 0.25549 |
| 346 | 0.24772 |
| 346.5 | 0.23873 |
| 347 | 0.23347 |
| 347.5 | 0.22281 |
| 348 | 0.21687 |
| 348.5 | 0.2082 |
| 349 | 0.20113 |
| 349.5 | 0.19283 |
| 350 | 0.1856 |
| 350.5 | 0.17985 |
| 351 | 0.17278 |
| 351.5 | 0.16591 |
| 352 | 0.15916 |
| 352.5 | 0.14989 |
| 353 | 0.14358 |
| 353.5 | 0.13612 |
| 354 | 0.12951 |
| 354.5 | 0.12519 |
| 355 | 0.11674 |
| 355.5 | 0.11243 |
| 356 | 0.10674 |
| 356.5 | 0.10025 |
| 357 | 0.09498 |
| 357.5 | 0.08929 |
| 358 | 0.08195 |
| 358.5 | 0.08094 |
| 359 | 0.07212 |
| 359.5 | 0.07003 |
| 360 | 0.0656 |
| 360.5 | 0.06118 |
| 361 | 0.05753 |
| 361.5 | 0.05668 |
| 362 | 0.04922 |
| 362.5 | 0.04641 |
| 363 | 0.04378 |
| 363.5 | 0.04073 |
| 364 | 0.03578 |
| 364.5 | 0.03432 |
| 365 | 0.03055 |
| 365.5 | 0.03116 |
| 366 | 0.02482 |
| 366.5 | 0.02563 |
| 367 | 0.02449 |
| 367.5 | 0.02093 |
| 368 | 0.01944 |
| 368.5 | 0.01704 |
| 369 | 0.01454 |
| 369.5 | 0.01485 |
| 370 | 0.01139 |
| 370.5 | 0.0117 |
| 371 | 0.01051 |
| 371.5 | 0.00857 |
| 372 | 0.0094 |
| 372.5 | 0.00769 |
| 373 | 0.00842 |
| 373.5 | 0.00549 |
| 374 | 0.00327 |
| 374.5 | 0.00262 |
| 375 | 0.00493 |
| 375.5 | 0.00257 |
| 376 | 0.00353 |
| 376.5 | 0.00151 |
| 377 | 0.00138 |
| 377.5 | 0.00197 |
| 378 | 0.00178 |
| 378.5 | 0.00141 |
| 379 | 0.00218 |
| 379.5 | 0.00078 |
| 380 | 0 |
| 380.5 | 5e-05 |
| 381 | 0.00068 |
| 381.5 | 0.00082 |
| 382 | -0.00177 |
| 382.5 | -0.0009 |
| 383 | -0.00462 |
| 383.5 | -0.00149 |
| 384 | -0.00207 |
| 384.5 | -0.00376 |
| 385 | -0.00102 |
| 385.5 | -0.00172 |
| 386 | -0.00071 |
| 386.5 | -0.00194 |
| 387 | -0.00289 |
| 387.5 | -0.00247 |
| 388 | -0.00188 |
| 388.5 | -0.00141 |
| 389 | -0.00293 |
| 389.5 | -0.00101 |
| 390 | -0.00173 |
| 390.5 | -0.00185 |
| 391 | -0.00375 |
| 391.5 | -0.00244 |
| 392 | -0.00224 |
| 392.5 | -0.00361 |
| 393 | -0.00366 |
| 393.5 | -0.00294 |
| 394 | -0.00308 |
| 394.5 | -0.003 |
| 395 | -0.0029 |
| 395.5 | -0.0034 |
| 396 | -0.00429 |
| 396.5 | -0.00354 |
| 397 | -0.00267 |
| 397.5 | -0.00398 |
| 398 | -0.0041 |
| 398.5 | -0.00182 |
| 399 | -0.00421 |
| 399.5 | -0.00358 |
| 400 | -0.00463 |
| 400.5 | -0.00481 |
| 401 | -0.00277 |
| 401.5 | -0.00395 |
| 402 | -0.00394 |
| 402.5 | -0.00427 |
| 403 | -0.00349 |
| 403.5 | -0.00372 |
| 404 | -0.00462 |
| 404.5 | -0.00322 |
| 405 | -0.00312 |
| 405.5 | -0.0033 |
| 406 | -0.00358 |
| 406.5 | -0.00324 |
| 407 | -0.00434 |
| 407.5 | -0.00405 |
| 408 | -0.0044 |
| 408.5 | -0.00288 |
| 409 | -0.00376 |
| 409.5 | -0.00413 |
| 410 | -0.00391 |
| 410.5 | -0.00388 |
| 411 | -0.00367 |
| 411.5 | -0.0041 |
| 412 | -0.00289 |
| 412.5 | -0.00526 |
| 413 | -0.00302 |
| 413.5 | -0.00361 |
| 414 | -0.00431 |
| 414.5 | -0.00382 |
| 415 | -0.004 |
| 415.5 | -0.00365 |
| 416 | -0.00361 |
| 416.5 | -0.00503 |
| 417 | -0.00303 |
| 417.5 | -0.00336 |
| 418 | -0.00415 |
| 418.5 | -0.00367 |
| 419 | -0.00327 |
| 419.5 | -0.00291 |
| 420 | -0.00489 |
| 420.5 | -0.0029 |
| 421 | -0.00336 |
| 421.5 | -0.00406 |
| 422 | -0.00306 |
| 422.5 | -0.00494 |

| Wavelength (nm) | Toluene |
| --- | --- |
| 250 | 0.3995 |
| 251 | 0.38947 |
| 252 | 0.37348 |
| 253 | 0.38133 |
| 254 | 0.36694 |
| 255 | 0.37648 |
| 256 | 0.37114 |
| 257 | 0.36977 |
| 258 | 0.36646 |
| 259 | 0.35569 |
| 260 | 0.34353 |
| 261 | 0.35787 |
| 262 | 0.36178 |
| 263 | 0.3492 |
| 264 | 0.34457 |
| 265 | 0.33367 |
| 266 | 0.32245 |
| 267 | 0.31312 |
| 268 | 0.30392 |
| 269 | 0.30136 |
| 270 | 0.29982 |
| 271 | 0.29803 |
| 272 | 0.2991 |
| 273 | 0.2991 |
| 274 | 0.29544 |
| 275 | 0.28641 |
| 276 | 0.28195 |
| 277 | 0.27661 |
| 278 | 0.2744 |
| 279 | 0.27354 |
| 280 | 0.27619 |
| 281 | 0.27927 |
| 282 | 0.28204 |
| 283 | 0.2851 |
| 284 | 0.2881 |
| 285 | 0.29039 |
| 286 | 0.2936 |
| 287 | 0.29681 |
| 288 | 0.30093 |
| 289 | 0.30556 |
| 290 | 0.31087 |
| 291 | 0.31723 |
| 292 | 0.32429 |
| 293 | 0.33194 |
| 294 | 0.34038 |
| 295 | 0.34966 |
| 296 | 0.35937 |
| 297 | 0.36947 |
| 298 | 0.38018 |
| 299 | 0.39143 |
| 300 | 0.40277 |
| 301 | 0.41428 |
| 302 | 0.42568 |
| 303 | 0.43726 |
| 304 | 0.44855 |
| 305 | 0.45983 |
| 306 | 0.47087 |
| 307 | 0.48184 |
| 308 | 0.4924 |
| 309 | 0.50261 |
| 310 | 0.51262 |
| 311 | 0.52229 |
| 312 | 0.53176 |
| 313 | 0.54074 |
| 314 | 0.54964 |
| 315 | 0.55836 |
| 316 | 0.56692 |
| 317 | 0.57487 |
| 318 | 0.58237 |
| 319 | 0.58904 |
| 320 | 0.59486 |
| 321 | 0.5997 |
| 322 | 0.60334 |
| 323 | 0.60561 |
| 324 | 0.60647 |
| 325 | 0.60614 |
| 326 | 0.60441 |
| 327 | 0.60124 |
| 328 | 0.5966 |
| 329 | 0.59033 |
| 330 | 0.58288 |
| 331 | 0.57406 |
| 332 | 0.5641 |
| 333 | 0.55272 |
| 334 | 0.54015 |
| 335 | 0.52627 |
| 336 | 0.51127 |
| 337 | 0.49538 |
| 338 | 0.4786 |
| 339 | 0.46124 |
| 340 | 0.44329 |
| 341 | 0.42516 |
| 342 | 0.40627 |
| 343 | 0.38716 |
| 344 | 0.36773 |
| 345 | 0.34755 |
| 346 | 0.32722 |
| 347 | 0.30704 |
| 348 | 0.28707 |
| 349 | 0.26722 |
| 350 | 0.24836 |
| 351 | 0.23015 |
| 352 | 0.21223 |
| 353 | 0.19491 |
| 354 | 0.17829 |
| 355 | 0.16213 |
| 356 | 0.14689 |
| 357 | 0.13233 |
| 358 | 0.11857 |
| 359 | 0.1049 |
| 360 | 0.09213 |
| 361 | 0.0804 |
| 362 | 0.06965 |
| 363 | 0.06018 |
| 364 | 0.05204 |
| 365 | 0.0446 |
| 366 | 0.03795 |
| 367 | 0.03211 |
| 368 | 0.02692 |
| 369 | 0.02234 |
| 370 | 0.01859 |
| 371 | 0.01518 |
| 372 | 0.01222 |
| 373 | 0.00977 |
| 374 | 0.00755 |
| 375 | 0.00567 |
| 376 | 0.00419 |
| 377 | 0.00288 |
| 378 | 0.00173 |
| 379 | 0.00076 |
| 380 | 0 |
| 381 | -0.00067 |
| 382 | -0.00113 |
| 383 | -0.00159 |
| 384 | -0.00199 |
| 385 | -0.00229 |
| 386 | -0.00265 |
| 387 | -0.00283 |
| 388 | -0.00304 |
| 389 | -0.00326 |
| 390 | -0.00349 |
| 391 | -0.00354 |
| 392 | -0.00364 |
| 393 | -0.00374 |
| 394 | -0.00378 |
| 395 | -0.00379 |
| 396 | -0.00388 |
| 397 | -0.00391 |
| 398 | -0.00392 |
| 399 | -0.0039 |
| 400 | -0.0039 |
| 401 | -0.00398 |
| 402 | -0.00405 |
| 403 | -0.00418 |
| 404 | -0.00421 |
| 405 | -0.00435 |
| 406 | -0.00422 |
| 407 | -0.0043 |
| 408 | -0.00424 |
| 409 | -0.0043 |
| 410 | -0.00425 |
| 411 | -0.0043 |
| 412 | -0.00428 |
| 413 | -0.00428 |
| 414 | -0.00434 |
| 415 | -0.00426 |
| 416 | -0.00425 |
| 417 | -0.00417 |
| 418 | -0.00427 |
| 419 | -0.00426 |
| 420 | -0.00436 |
| 421 | -0.00439 |
| 422 | -0.00435 |
| 423 | -0.00436 |
| 424 | -0.00431 |
| 425 | -0.00429 |
| 426 | -0.00429 |
| 427 | -0.00435 |
| 428 | -0.00436 |
| 429 | -0.00445 |
| 430 | -0.00459 |
| 431 | -0.00457 |
| 432 | -0.00453 |
| 433 | -0.00456 |
| 434 | -0.00444 |
| 435 | -0.00443 |
| 436 | -0.00446 |
| 437 | -0.00452 |
| 438 | -0.00449 |
| 439 | -0.0045 |
| 440 | -0.00453 |
| 441 | -0.00439 |
| 442 | -0.00446 |
| 443 | -0.00445 |
| 444 | -0.00444 |
| 445 | -0.00461 |
| 446 | -0.00469 |
| 447 | -0.00479 |
| 448 | -0.00481 |
| 449 | -0.00475 |

| Wavelength (nm) | THF | 1,4-dioxane | DMF | Acetonitrile |
| --- | --- | --- | --- | --- |
| 290 | 0.08614 | 0.0881 | -2.28888 | -0.11333 |
| 290.5 | 0.06937 | 0.08705 | -2.32011 | -0.11032 |
| 291 | 0.0755 | 0.08266 | -2.28053 | -0.11363 |
| 291.5 | 0.07305 | 0.0839 | -2.31638 | -0.11103 |
| 292 | 0.06823 | 0.08484 | -2.12242 | -0.11041 |
| 292.5 | 0.06343 | 0.07842 | -2.1014 | -0.10808 |
| 293 | 0.06174 | 0.0753 | -2.02181 | -0.11011 |
| 293.5 | 0.06268 | 0.07291 | -2.00763 | -0.10305 |
| 294 | 0.05815 | 0.06827 | -2.00372 | -0.10465 |
| 294.5 | 0.05974 | 0.06278 | -2.02985 | -0.10263 |
| 295 | 0.0568 | 0.06257 | -1.90957 | -0.09544 |
| 295.5 | 0.05351 | 0.06082 | -1.85024 | -0.09704 |
| 296 | 0.05447 | 0.0572 | -1.8405 | -0.09229 |
| 296.5 | 0.04792 | 0.05094 | -1.79789 | -0.0863 |
| 297 | 0.04641 | 0.05312 | -1.69452 | -0.08857 |
| 297.5 | 0.04204 | 0.05361 | -1.66818 | -0.08951 |
| 298 | 0.0467 | 0.04747 | -1.58796 | -0.08364 |
| 298.5 | 0.04106 | 0.04344 | -1.53038 | -0.08307 |
| 299 | 0.037 | 0.03976 | -1.50527 | -0.07683 |
| 299.5 | 0.03918 | 0.04104 | -1.44224 | -0.07928 |
| 300 | 0.0387 | 0.03945 | -1.40016 | -0.07327 |
| 300.5 | 0.0343 | 0.03056 | -1.35113 | -0.07341 |
| 301 | 0.03159 | 0.03909 | -1.28923 | -0.07147 |
| 301.5 | 0.03343 | 0.03066 | -1.23306 | -0.06599 |
| 302 | 0.02989 | 0.02968 | -1.17678 | -0.06469 |
| 302.5 | 0.02626 | 0.03288 | -1.14874 | -0.06419 |
| 303 | 0.02753 | 0.02833 | -1.10576 | -0.05828 |
| 303.5 | 0.02964 | 0.02227 | -1.05045 | -0.05757 |
| 304 | 0.02742 | 0.02848 | -1.01107 | -0.05339 |
| 304.5 | 0.02353 | 0.02052 | -0.97732 | -0.05439 |
| 305 | 0.02228 | 0.02029 | -0.91968 | -0.04574 |
| 305.5 | 0.01307 | 0.01634 | -0.8929 | -0.04567 |
| 306 | 0.02357 | 0.01549 | -0.8512 | -0.04208 |
| 306.5 | 0.02109 | 0.01618 | -0.82016 | -0.0441 |
| 307 | 0.01817 | 0.01486 | -0.771 | -0.03996 |
| 307.5 | 0.01674 | 0.0156 | -0.72857 | -0.03773 |
| 308 | 0.01825 | 0.01613 | -0.66829 | -0.03719 |
| 308.5 | 0.01554 | 0.01253 | -0.6386 | -0.03128 |
| 309 | 0.0202 | 0.01063 | -0.60161 | -0.02814 |
| 309.5 | 0.01458 | 0.01021 | -0.57838 | -0.02821 |
| 310 | 0.01483 | 0.00994 | -0.5437 | -0.02666 |
| 310.5 | 0.01801 | 0.00893 | -0.50774 | -0.02372 |
| 311 | 0.01592 | 0.00852 | -0.47978 | -0.02192 |
| 311.5 | 0.01491 | 0.01075 | -0.44717 | -0.01734 |
| 312 | 0.0134 | 0.00993 | -0.42443 | -0.01709 |
| 312.5 | 0.01453 | 0.00757 | -0.38529 | -0.01425 |
| 313 | 0.01059 | 0.00708 | -0.36866 | -0.01855 |
| 313.5 | 0.01493 | 0.00395 | -0.33569 | -0.01121 |
| 314 | 0.01246 | 0.00889 | -0.31021 | -0.0109 |
| 314.5 | 0.01373 | 0.00596 | -0.28259 | -0.01097 |
| 315 | 0.01257 | 0.00623 | -0.25939 | -0.0061 |
| 315.5 | 0.01201 | 0.0121 | -0.23735 | -0.00673 |
| 316 | 0.01091 | 0.00578 | -0.21727 | -0.0067 |
| 316.5 | 0.01402 | 0.00563 | -0.19511 | -0.00633 |
| 317 | 0.01275 | 0.0089 | -0.17809 | -0.00584 |
| 317.5 | 0.01129 | 0.00701 | -0.16063 | -0.00238 |
| 318 | 0.01095 | 0.00708 | -0.14406 | -0.0007 |
| 318.5 | 0.0119 | 0.00411 | -0.13309 | 0.00076 |
| 319 | 0.00909 | 0.00291 | -0.11764 | -0.00113 |
| 319.5 | 0.01144 | 0.00158 | -0.10619 | -0.00207 |
| 320 | 0.01274 | 0.00342 | -0.09147 | 0.00076 |
| 320.5 | 0.00966 | 0.0056 | -0.08444 | 0.00336 |
| 321 | 0.00927 | 0.00384 | -0.07677 | 0.00157 |
| 321.5 | 0.01255 | 0.00369 | -0.0622 | 0.00205 |
| 322 | 0.0094 | 0.00876 | -0.05634 | 0.00094 |
| 322.5 | 0.01125 | 0.00616 | -0.04766 | 0.00153 |
| 323 | 0.00809 | 0.0024 | -0.04477 | 0.00277 |
| 323.5 | 0.01132 | 0.00291 | -0.03166 | 0.00423 |
| 324 | 0.00706 | 0.00899 | -0.03212 | 0.00502 |
| 324.5 | 0.00944 | 0.00249 | -0.02691 | 0.00498 |
| 325 | 0.009 | 0.00305 | -0.02101 | 0.00354 |
| 325.5 | 0.01254 | 0.00199 | -0.01745 | 0.00547 |
| 326 | 0.00435 | 0.00354 | -0.01464 | 0.00415 |
| 326.5 | 0.01075 | 0.0017 | -0.0145 | 0.00383 |
| 327 | 0.00696 | 0.00543 | -0.01269 | 0.00696 |
| 327.5 | 0.00794 | 0.00435 | -0.00945 | 0.00345 |
| 328 | 0.00668 | 0.00292 | -0.00552 | 0.00436 |
| 328.5 | 0.00847 | 0.00533 | -0.00461 | 0.00586 |
| 329 | 0.00662 | 0.00155 | -0.00142 | 0.00249 |
| 329.5 | 0.00992 | 0.00462 | -0.0001 | 0.0049 |
| 330 | 0.00754 | 0.00351 | 0.00355 | 0.00521 |
| 330.5 | 0.00593 | 0.00485 | -0.00253 | 0.00291 |
| 331 | 0.00746 | 0.00302 | 0.00085 | 0.00238 |
| 331.5 | 0.00765 | 0.00275 | 0.00135 | 0.00407 |
| 332 | 0.00695 | 0.00445 | 0.00408 | 0.00331 |
| 332.5 | 0.00841 | 0.00471 | 0.00365 | 0.00392 |
| 333 | 0.00625 | 0.00275 | 0.00286 | 0.0018 |
| 333.5 | 0.00832 | 0.00172 | 0.00433 | 0.00443 |
| 334 | 0.00721 | 0.0041 | 0.00219 | 0.00128 |
| 334.5 | 0.007 | 0.00161 | 0.0047 | 0.00207 |
| 335 | 0.00522 | 0.00311 | 0.00591 | 0.00442 |
| 335.5 | 0.00462 | 0.00493 | 0.00457 | 0.0027 |
| 336 | 0.00654 | 0.00256 | 0.0054 | 0.00252 |
| 336.5 | 0.0047 | 0.00261 | 0.00766 | 0.00451 |
| 337 | 0.00735 | 0.00137 | 0.00568 | 0.00131 |
| 337.5 | 0.00613 | 0.00065 | 0.0061 | 0.00496 |
| 338 | 0.00582 | 0.00177 | 0.00619 | 0.00172 |
| 338.5 | 0.00548 | 0.00298 | 0.00668 | 0.00254 |
| 339 | 0.00554 | 0.00289 | 0.00628 | 0.00464 |
| 339.5 | 0.00407 | 0.00155 | 0.00689 | 0.00384 |
| 340 | 0.00425 | 0.00102 | 0.00444 | 0.00267 |
| 340.5 | 0.00466 | 0.00162 | 0.00403 | 0.00124 |
| 341 | 0.00586 | -0.00081 | 0.00556 | 0.0005 |
| 341.5 | 0.00581 | -0.00154 | 0.00403 | 0.00271 |
| 342 | 0.00555 | -0.00147 | 0.00749 | 0.00523 |
| 342.5 | 0.00598 | 0.00119 | 0.00504 | 0.00202 |
| 343 | 0.00477 | 0.00146 | 0.0065 | 0.00388 |
| 343.5 | 0.00593 | -0.00054 | 0.00546 | 0.00035 |
| 344 | 0.00532 | -0.00061 | 0.00528 | 0.00193 |
| 344.5 | 0.00238 | 0.00305 | 0.00603 | 0.00439 |
| 345 | 0.00392 | -0.00053 | 0.00541 | 0.003 |
| 345.5 | 0.00477 | 0.00029 | 0.00382 | -7e-05 |
| 346 | 0.00274 | -0.00012 | 0.00531 | 0.00262 |
| 346.5 | 0.00454 | -0.00166 | 0.00762 | 0.0009 |
| 347 | 0.0043 | 0.0023 | 0.00581 | 0.00235 |
| 347.5 | 0.00615 | -0.00046 | 0.00615 | 0.00021 |
| 348 | 0.00514 | 0.00168 | 0.00499 | 0.0001 |
| 348.5 | 0.0065 | -0.00036 | 0.00241 | 0.00221 |
| 349 | 0.00419 | -0.00037 | 0.00346 | 0.00274 |
| 349.5 | 0.00379 | -0.00111 | 0.00222 | -0.0003 |
| 350 | 0.00227 | -0.00134 | 0.00429 | 0.00265 |
| 350.5 | 0.00468 | -0.00116 | 0.00404 | 0.00215 |
| 351 | 0.00397 | -0.00306 | 0.00386 | 0.00145 |
| 351.5 | 0.00241 | -6e-05 | 0.00192 | 0.0019 |
| 352 | 0.0054 | -0.00132 | 0.00416 | 0.00166 |
| 352.5 | 0.00342 | -0.00214 | 0.00321 | -0.00052 |
| 353 | 0.00315 | 0.00283 | 0.00393 | 0.00123 |
| 353.5 | 0.00259 | -0.00048 | 0.00273 | 0.0004 |
| 354 | 0.00318 | 0.00115 | 0.00371 | -0.00017 |
| 354.5 | 0.0016 | -0.00022 | 0.00193 | -0.00048 |
| 355 | 0.00161 | -0.00116 | 0.00559 | -0.00194 |
| 355.5 | 0.00329 | -0.0011 | 0.00451 | -0.00112 |
| 356 | 0.00478 | -0.00022 | 0.00488 | 5e-05 |
| 356.5 | 0.00206 | -0.00024 | 0.00443 | 0.00078 |
| 357 | 0.00315 | 0.00042 | 0.00193 | -0.00045 |
| 357.5 | 0.00144 | 0.00012 | 0.00319 | 0.00145 |
| 358 | 0.0036 | 5e-05 | 0.0036 | -0.0019 |
| 358.5 | 0.00126 | -0.00125 | 0.00194 | 0.0012 |
| 359 | 0.00134 | -0.00098 | 0.00196 | -0.00127 |
| 359.5 | 0.00159 | -0.00068 | 0.00215 | -0.00019 |
| 360 | 0.00271 | -0.00126 | 0.00347 | -0.00037 |
| 360.5 | 0.00269 | -0.00164 | 0.00258 | 0.00034 |
| 361 | -0.00014 | 0.00118 | 0.00479 | -0.0009 |
| 361.5 | 0.00419 | 0.00026 | 0.00264 | -0.0023 |
| 362 | 0.00324 | -0.00197 | 0.0043 | -0.00069 |
| 362.5 | 0.00119 | 0.00044 | 0.00256 | 0.00135 |
| 363 | 0.00147 | -0.00312 | 0.0021 | 0.00131 |
| 363.5 | 0.00252 | 0.00072 | 0.00249 | -0.00078 |
| 364 | 0.00226 | 0.00087 | 0.00195 | -0.0007 |
| 364.5 | 0.00074 | -0.00035 | 0.0012 | 0.00076 |
| 365 | 8e-05 | -0.00121 | 0.004 | -1e-05 |
| 365.5 | 0.00135 | 0.00015 | 0.00113 | -0.00229 |
| 366 | 0.00106 | -0.0013 | 0.00032 | -0.00086 |
| 366.5 | 0.00282 | -0.00322 | 0.00369 | -0.00094 |
| 367 | 0.00115 | -0.00058 | 0.00134 | -0.00086 |
| 367.5 | 0.00084 | -0.00112 | 0.00143 | 0.00043 |
| 368 | 0.00127 | -0.00029 | 0.00315 | -4e-05 |
| 368.5 | -0.00018 | -0.00099 | 0.00138 | -0.00011 |
| 369 | 0.00125 | 0.00074 | 0.0023 | -0.00051 |
| 369.5 | 0.00161 | -0.00034 | 0.00075 | 0.00012 |
| 370 | 0.00343 | 0.00084 | 0.00086 | 0.00053 |
| 370.5 | -0.0004 | -0.00021 | 0.00037 | -0.00205 |
| 371 | 0.00127 | -0.00084 | 0.00231 | 0.00084 |
| 371.5 | 0.00022 | -0.00211 | 5e-05 | -0.00017 |
| 372 | 0.00212 | 0.00117 | 0.00144 | -0.00238 |
| 372.5 | 0.00243 | -0.00132 | 0.00046 | -0.00286 |
| 373 | 0.0016 | 0.00033 | 0.00139 | -0.00086 |
| 373.5 | -0.00145 | 0.0005 | 0.00136 | -0.0013 |
| 374 | 0.00023 | 0.00154 | 0.00081 | -0.00149 |
| 374.5 | 0.00075 | 0.00056 | 0.0017 | -0.00033 |
| 375 | -0.00054 | 6e-05 | 0.00052 | -0.00114 |
| 375.5 | 0.0012 | 0.00018 | 0.00331 | -9e-05 |
| 376 | 0.00226 | -0.00062 | 0.00152 | 0.00046 |
| 376.5 | -5e-05 | 4e-05 | 0.00177 | 0.00029 |
| 377 | 0.00216 | 0.00131 | 0.00028 | 9e-05 |
| 377.5 | 0.0016 | 0.00189 | 0.00146 | -0.00226 |
| 378 | 0.0002 | 0.00171 | 0.00177 | -0.00097 |
| 378.5 | 0.00113 | -0.00033 | -0.00029 | -0.00075 |
| 379 | -0.00029 | 0.00093 | -0.00064 | -0.00084 |
| 379.5 | -0.00111 | 0.00203 | 0.00146 | -0.0003 |
| 380 | 0 | 0 | 0 | 0 |
| 380.5 | -0.00018 | 0.00125 | 0.00087 | 0.00023 |
| 381 | 0.00014 | -8e-05 | 0.00143 | -0.00156 |
| 381.5 | 0.00065 | 0.00105 | 0.0006 | 0.00041 |
| 382 | -0.00081 | 0.00148 | -0.00183 | -0.00109 |
| 382.5 | -0.0014 | 0.00226 | 0.00052 | -0.00021 |
| 383 | 0.00193 | -0.00231 | 0.00022 | -0.00055 |
| 383.5 | -4e-05 | -0.00058 | 0.00143 | -0.00074 |
| 384 | -0.00159 | 0.00181 | -0.00115 | -0.0003 |
| 384.5 | 0.0012 | 0.00028 | 0.00226 | -4e-05 |
| 385 | -0.00137 | 0.00064 | -0.00094 | -0.00024 |
| 385.5 | -0.00047 | -7e-05 | 0.0008 | 0.00111 |
| 386 | 0.00031 | 0.00219 | -0.00082 | 0.00012 |
| 386.5 | -0.00023 | 0.00236 | 0.00032 | 0.00125 |
| 387 | -0.00139 | 0.00036 | -0.0005 | -0.00011 |
| 387.5 | 0.00085 | 0.00226 | -0.00018 | 0.00076 |
| 388 | 0.00072 | 0.00304 | 9e-05 | -0.00072 |
| 388.5 | -0.00066 | 0.00215 | -0.00019 | 0.00019 |
| 389 | -0.00128 | 0.00173 | -0.00167 | -0.00129 |
| 389.5 | -0.00014 | 0.00089 | -0.00169 | -0.00049 |

| Wavelength (nm) | Acetone |
| --- | --- |
| 323 | -0.02822 |
| 323.5 | 0.01146 |
| 324 | 0.01933 |
| 324.5 | -0.00126 |
| 325 | 0.02498 |
| 325.5 | 0.02933 |
| 326 | 0.00181 |
| 326.5 | 0.00145 |
| 327 | 0.00064 |
| 327.5 | 0.00309 |
| 328 | 0.01023 |
| 328.5 | 0.00205 |
| 329 | -0.0046 |
| 329.5 | 0.00052 |
| 330 | 0.0033 |
| 330.5 | 0.00592 |
| 331 | 0.00094 |
| 331.5 | 0.00544 |
| 332 | 0.00352 |
| 332.5 | 0.00372 |
| 333 | 0.00416 |
| 333.5 | 0.00233 |
| 334 | 0.00299 |
| 334.5 | 0.00067 |
| 335 | 0.00375 |
| 335.5 | 0.00162 |
| 336 | 0.00662 |
| 336.5 | 0.00447 |
| 337 | 0.001 |
| 337.5 | 0.00568 |
| 338 | 0.00037 |
| 338.5 | -7e-05 |
| 339 | -0.00053 |
| 339.5 | 0.0036 |
| 340 | 0.00353 |
| 340.5 | 0.00145 |
| 341 | 0.00197 |
| 341.5 | -3e-05 |
| 342 | -0.0004 |
| 342.5 | 0.00318 |
| 343 | 0.00072 |
| 343.5 | 0.00039 |
| 344 | -1e-05 |
| 344.5 | 0.00091 |
| 345 | -0.00079 |
| 345.5 | 0.00177 |
| 346 | -0.00045 |
| 346.5 | 0.00105 |
| 347 | -0.00141 |
| 347.5 | -0.00107 |
| 348 | 0.00045 |
| 348.5 | 0 |
| 349 | -0.00088 |
| 349.5 | 0.0004 |
| 350 | -0.00282 |
| 350.5 | 0.00084 |
| 351 | 0.00032 |
| 351.5 | -0.00104 |
| 352 | -0.0008 |
| 352.5 | -0.00072 |
| 353 | -0.00083 |
| 353.5 | -0.00055 |
| 354 | 0.00059 |
| 354.5 | -0.00176 |
| 355 | -0.00349 |
| 355.5 | -0.00025 |
| 356 | -0.00195 |
| 356.5 | -7e-05 |
| 357 | -7e-05 |
| 357.5 | -0.00187 |
| 358 | -0.00177 |
| 358.5 | -0.0021 |
| 359 | -0.00195 |
| 359.5 | -0.00365 |
| 360 | -0.00033 |
| 360.5 | 9e-05 |
| 361 | -0.00066 |
| 361.5 | -0.00294 |
| 362 | -0.00209 |
| 362.5 | 0.00028 |
| 363 | -0.00315 |
| 363.5 | -0.00472 |
| 364 | -0.00291 |
| 364.5 | -0.00158 |
| 365 | -6e-05 |
| 365.5 | -1e-05 |
| 366 | -0.0019 |
| 366.5 | -0.0015 |
| 367 | 0.00065 |
| 367.5 | -0.00147 |
| 368 | 0.00051 |
| 368.5 | -0.0018 |
| 369 | -0.001 |
| 369.5 | 0.00056 |
| 370 | -0.00166 |
| 370.5 | -0.00074 |
| 371 | -0.00219 |
| 371.5 | -0.00122 |
| 372 | -0.00067 |
| 372.5 | -0.00147 |
| 373 | -0.00053 |
| 373.5 | -0.00108 |
| 374 | -0.00049 |
| 374.5 | -0.00177 |
| 375 | 0.00137 |
| 375.5 | 0.00043 |
| 376 | 5e-05 |
| 376.5 | -0.00061 |
| 377 | -0.00142 |
| 377.5 | 0.00018 |
| 378 | -0.0017 |
| 378.5 | -0.00057 |
| 379 | -0.0006 |
| 379.5 | -0.00076 |
| 380 | 0 |
| 380.5 | -0.00038 |
| 381 | 0.00122 |
| 381.5 | 7e-05 |
| 382 | -0.00119 |
| 382.5 | -5e-05 |
| 383 | 0.00054 |
| 383.5 | 0.00031 |
| 384 | -0.00092 |
| 384.5 | 0.00011 |
| 385 | -6e-05 |
| 385.5 | 0.00073 |
| 386 | 0.00153 |
| 386.5 | 0.00095 |
| 387 | 0.00042 |
| 387.5 | -0.00052 |
| 388 | 0.00101 |
| 388.5 | 0.00083 |
| 389 | 0.00076 |
| 389.5 | 0.0019 |
| 390 | 0.00204 |
| 390.5 | -0.00018 |
| 391 | 0.00162 |
| 391.5 | 0.00232 |
| 392 | 0.00082 |
| 392.5 | 0.00144 |
| 393 | 0.00063 |
| 393.5 | 0.00139 |
| 394 | 0.00114 |
| 394.5 | 0.00119 |
| 395 | 0.00162 |
| 395.5 | 8e-05 |
| 396 | 0.00242 |
| 396.5 | 0.00297 |
| 397 | 0.00237 |
| 397.5 | 0.00172 |
| 398 | 0.00313 |
| 398.5 | 0.00259 |
| 399 | 0.00266 |
| 399.5 | 0.00243 |
| 400 | 0.00212 |
| 400.5 | 0.00325 |
| 401 | 0.00121 |
| 401.5 | 0.00302 |
| 402 | 0.00288 |
| 402.5 | 0.00247 |
| 403 | 0.00229 |
| 403.5 | 0.00214 |
| 404 | 0.00311 |
| 404.5 | 0.00293 |
| 405 | 0.00108 |
| 405.5 | 0.00337 |
| 406 | 0.00265 |
| 406.5 | 0.00277 |
| 407 | 0.0021 |
| 407.5 | 0.00182 |
| 408 | 0.00316 |
| 408.5 | 0.00144 |
| 409 | 0.00281 |
| 409.5 | 0.00249 |
| 410 | 0.00195 |
| 410.5 | 0.00188 |
| 411 | 0.00185 |
| 411.5 | 0.00315 |
| 412 | 0.00146 |
| 412.5 | 0.00417 |
| 413 | 0.0019 |
| 413.5 | 0.00238 |
| 414 | 0.00213 |
| 414.5 | 0.00254 |
| 415 | 0.00317 |
| 415.5 | 0.00326 |
| 416 | 0.00283 |
| 416.5 | 0.0035 |
| 417 | 0.00192 |
| 417.5 | 0.00389 |
| 418 | 0.00271 |
| 418.5 | 0.00322 |
| 419 | 0.00359 |
| 419.5 | 0.003 |
| 420 | 0.00269 |
| 420.5 | 0.0019 |
| 421 | 0.00451 |
| 421.5 | 0.00267 |
| 422 | 0.00269 |
| 422.5 | 0.00343 |

| Wavelength (nm) | Toluene |
| --- | --- |
| 500 | -0.00323 |
| 499.5 | -0.002938 |
| 499 | -0.002517 |
| 498.5 | -0.002923 |
| 498 | -0.00251 |
| 497.5 | -0.002809 |
| 497 | -0.002573 |
| 496.5 | -0.002797 |
| 496 | -0.002576 |
| 495.5 | -0.00309 |
| 495 | -0.002477 |
| 494.5 | -0.003046 |
| 494 | -0.003049 |
| 493.5 | -0.002571 |
| 493 | -0.001986 |
| 492.5 | -0.002588 |
| 492 | -0.002556 |
| 491.5 | -0.002989 |
| 491 | -0.002675 |
| 490.5 | -0.003374 |
| 490 | -0.00243 |
| 489.5 | -0.002201 |
| 489 | -0.00225 |
| 488.5 | -0.002455 |
| 488 | -0.002295 |
| 487.5 | -0.002389 |
| 487 | -0.002824 |
| 486.5 | -0.002639 |
| 486 | -0.002718 |
| 485.5 | -0.002761 |
| 485 | -0.002534 |
| 484.5 | -0.002522 |
| 484 | -0.002239 |
| 483.5 | -0.002476 |
| 483 | -0.002564 |
| 482.5 | -0.002051 |
| 482 | -0.002836 |
| 481.5 | -0.002838 |
| 481 | -0.002254 |
| 480.5 | -0.002145 |
| 480 | -0.002502 |
| 479.5 | -0.002915 |
| 479 | -0.002633 |
| 478.5 | -0.002724 |
| 478 | -0.002852 |
| 477.5 | -0.002662 |
| 477 | -0.002426 |
| 476.5 | -0.002505 |
| 476 | -0.002469 |
| 475.5 | -0.002367 |
| 475 | -0.002045 |
| 474.5 | -0.002471 |
| 474 | -0.002252 |
| 473.5 | -0.002624 |
| 473 | -0.002228 |
| 472.5 | -0.002228 |
| 472 | -0.002406 |
| 471.5 | -0.002079 |
| 471 | -0.002157 |
| 470.5 | -0.00232 |
| 470 | -0.002375 |
| 469.5 | -0.002033 |
| 469 | -0.002522 |
| 468.5 | -0.002621 |
| 468 | -0.002248 |
| 467.5 | -0.002096 |
| 467 | -0.002051 |
| 466.5 | -0.002183 |
| 466 | -0.002312 |
| 465.5 | -0.002582 |
| 465 | -0.002331 |
| 464.5 | -0.002136 |
| 464 | -0.002282 |
| 463.5 | -0.002164 |
| 463 | -0.001989 |
| 462.5 | -0.002004 |
| 462 | -0.002007 |
| 461.5 | -0.001993 |
| 461 | -0.002033 |
| 460.5 | -0.001999 |
| 460 | -0.001896 |
| 459.5 | -0.001905 |
| 459 | -0.002041 |
| 458.5 | -0.002017 |
| 458 | -0.002094 |
| 457.5 | -0.002267 |
| 457 | -0.002204 |
| 456.5 | -0.001833 |
| 456 | -0.001601 |
| 455.5 | -0.001969 |
| 455 | -0.002094 |
| 454.5 | -0.00173 |
| 454 | -0.001935 |
| 453.5 | -0.001794 |
| 453 | -0.001958 |
| 452.5 | -0.002267 |
| 452 | -0.002444 |
| 451.5 | -0.002151 |
| 451 | -0.001648 |
| 450.5 | -0.001887 |
| 450 | -0.001914 |
| 449.5 | -0.001685 |
| 449 | -0.002023 |
| 448.5 | -0.002345 |
| 448 | -0.001806 |
| 447.5 | -0.001437 |
| 447 | -0.001794 |
| 446.5 | -0.001949 |
| 446 | -0.001832 |
| 445.5 | -0.001806 |
| 445 | -0.001885 |
| 444.5 | -0.001682 |
| 444 | -0.001874 |
| 443.5 | -0.002232 |
| 443 | -0.002141 |
| 442.5 | -0.001952 |
| 442 | -0.00211 |
| 441.5 | -0.002346 |
| 441 | -0.002038 |
| 440.5 | -0.001358 |
| 440 | -0.001533 |
| 439.5 | -0.001891 |
| 439 | -0.001905 |
| 438.5 | -0.001498 |
| 438 | -0.001571 |
| 437.5 | -0.002024 |
| 437 | -0.002241 |
| 436.5 | -0.002154 |
| 436 | -0.001906 |
| 435.5 | -0.001902 |
| 435 | -0.001825 |
| 434.5 | -0.001671 |
| 434 | -0.001669 |
| 433.5 | -0.001622 |
| 433 | -0.001403 |
| 432.5 | -0.00128 |
| 432 | -0.001476 |
| 431.5 | -0.001643 |
| 431 | -0.001414 |
| 430.5 | -0.001302 |
| 430 | -0.00145 |
| 429.5 | -0.001551 |
| 429 | -0.001826 |
| 428.5 | -0.001695 |
| 428 | -0.001774 |
| 427.5 | -0.001645 |
| 427 | -0.00167 |
| 426.5 | -0.001767 |
| 426 | -0.001455 |
| 425.5 | -0.001367 |
| 425 | -0.001727 |
| 424.5 | -0.002349 |
| 424 | -0.002205 |
| 423.5 | -0.001825 |
| 423 | -0.001456 |
| 422.5 | -0.001161 |
| 422 | -0.001294 |
| 421.5 | -0.001116 |
| 421 | -0.001406 |
| 420.5 | -0.00179 |
| 420 | -0.001332 |
| 419.5 | -0.001221 |
| 419 | -0.001679 |
| 418.5 | -0.001662 |
| 418 | -0.001193 |
| 417.5 | -0.000896 |
| 417 | -0.001146 |
| 416.5 | -0.001722 |
| 416 | -0.001515 |
| 415.5 | -0.001243 |
| 415 | -0.001082 |
| 414.5 | -0.001416 |
| 414 | -0.00171 |
| 413.5 | -0.001688 |
| 413 | -0.00167 |
| 412.5 | -0.001754 |
| 412 | -0.001588 |
| 411.5 | -0.001288 |
| 411 | -0.001591 |
| 410.5 | -0.001413 |
| 410 | -0.00087 |
| 409.5 | -0.001141 |
| 409 | -0.00138 |
| 408.5 | -0.001345 |
| 408 | -0.001403 |
| 407.5 | -0.001412 |
| 407 | -0.000777 |
| 406.5 | -0.001146 |
| 406 | -0.001306 |
| 405.5 | -0.00141 |
| 405 | -0.001012 |
| 404.5 | -0.001509 |
| 404 | -0.001867 |
| 403.5 | -0.001656 |
| 403 | -0.000679 |
| 402.5 | -0.000907 |
| 402 | -0.001071 |
| 401.5 | -0.001028 |
| 401 | -0.001404 |
| 400.5 | -0.001627 |

*UVA (315-400 nm)*

**Figure S1** NBD solutions (4.48 x 10^-5^ M) recorded in different solvents prior to and after 1 hour of UVA irradiation.

**Figure S2** **NBD1** to **QC1** photoisomerisation study in dichloromethane.

Absorbance (a.u)

| Wavelength (nm) | NBD | 1 hour UVA | 3 hour UVA | 2 hour UVA |
| --- | --- | --- | --- | --- |
| 290 | 0.23215 | 0.17803 | 0.15202 | 0.08714 |
| 290.5 | 0.23349 | 0.17776 | 0.15751 | 0.08765 |
| 291 | 0.23352 | 0.18358 | 0.15477 | 0.09026 |
| 291.5 | 0.2385 | 0.18095 | 0.14967 | 0.0904 |
| 292 | 0.2444 | 0.17927 | 0.15278 | 0.08694 |
| 292.5 | 0.24725 | 0.18429 | 0.15121 | 0.08906 |
| 293 | 0.24994 | 0.18384 | 0.1477 | 0.08715 |
| 293.5 | 0.25407 | 0.18524 | 0.15221 | 0.09227 |
| 294 | 0.25502 | 0.18365 | 0.14713 | 0.0911 |
| 294.5 | 0.25121 | 0.19047 | 0.14523 | 0.09111 |
| 295 | 0.2592 | 0.18827 | 0.14562 | 0.08906 |
| 295.5 | 0.26384 | 0.19377 | 0.14745 | 0.09552 |
| 296 | 0.2675 | 0.19394 | 0.14719 | 0.0922 |
| 296.5 | 0.26826 | 0.1976 | 0.14486 | 0.09105 |
| 297 | 0.27293 | 0.19657 | 0.14708 | 0.09404 |
| 297.5 | 0.28089 | 0.20225 | 0.14764 | 0.09555 |
| 298 | 0.27759 | 0.19756 | 0.14751 | 0.09162 |
| 298.5 | 0.27966 | 0.20314 | 0.14405 | 0.09086 |
| 299 | 0.28533 | 0.20486 | 0.15154 | 0.09847 |
| 299.5 | 0.28803 | 0.21003 | 0.14336 | 0.09632 |
| 300 | 0.29059 | 0.20932 | 0.14523 | 0.09945 |
| 300.5 | 0.29782 | 0.21182 | 0.14535 | 0.09852 |
| 301 | 0.30143 | 0.21356 | 0.14329 | 0.09703 |
| 301.5 | 0.31309 | 0.21225 | 0.14679 | 0.0997 |
| 302 | 0.31493 | 0.2156 | 0.14541 | 0.10164 |
| 302.5 | 0.31918 | 0.22364 | 0.1468 | 0.10194 |
| 303 | 0.3216 | 0.2242 | 0.14955 | 0.10414 |
| 303.5 | 0.32763 | 0.22911 | 0.15205 | 0.10327 |
| 304 | 0.33254 | 0.22745 | 0.14767 | 0.10633 |
| 304.5 | 0.33686 | 0.22993 | 0.15045 | 0.10903 |
| 305 | 0.34399 | 0.23283 | 0.14966 | 0.1079 |
| 305.5 | 0.34951 | 0.23583 | 0.14769 | 0.10843 |
| 306 | 0.35747 | 0.24096 | 0.15421 | 0.10804 |
| 306.5 | 0.35299 | 0.24216 | 0.15105 | 0.11092 |
| 307 | 0.36244 | 0.24284 | 0.15437 | 0.11407 |
| 307.5 | 0.36744 | 0.24902 | 0.15131 | 0.11257 |
| 308 | 0.37032 | 0.25082 | 0.15666 | 0.11527 |
| 308.5 | 0.37453 | 0.2546 | 0.154 | 0.11774 |
| 309 | 0.38123 | 0.25749 | 0.15662 | 0.11682 |
| 309.5 | 0.38501 | 0.26126 | 0.15519 | 0.12038 |
| 310 | 0.38757 | 0.26119 | 0.1564 | 0.11977 |
| 310.5 | 0.38994 | 0.26484 | 0.15257 | 0.11833 |
| 311 | 0.39412 | 0.26956 | 0.15603 | 0.11894 |
| 311.5 | 0.39748 | 0.27435 | 0.15924 | 0.12275 |
| 312 | 0.39955 | 0.2703 | 0.157 | 0.12455 |
| 312.5 | 0.40557 | 0.27009 | 0.15914 | 0.12752 |
| 313 | 0.40754 | 0.27839 | 0.16108 | 0.12359 |
| 313.5 | 0.40938 | 0.27879 | 0.15999 | 0.12644 |
| 314 | 0.41424 | 0.27962 | 0.16278 | 0.12756 |
| 314.5 | 0.41712 | 0.27999 | 0.16528 | 0.12391 |
| 315 | 0.42817 | 0.28461 | 0.16446 | 0.13234 |
| 315.5 | 0.42782 | 0.28676 | 0.16547 | 0.13232 |
| 316 | 0.43469 | 0.29086 | 0.16645 | 0.13096 |
| 316.5 | 0.43801 | 0.29689 | 0.17021 | 0.13121 |
| 317 | 0.43888 | 0.29661 | 0.17037 | 0.13364 |
| 317.5 | 0.44123 | 0.30073 | 0.17065 | 0.13658 |
| 318 | 0.44771 | 0.29897 | 0.1714 | 0.13516 |
| 318.5 | 0.44954 | 0.30226 | 0.16745 | 0.13929 |
| 319 | 0.45523 | 0.30444 | 0.17211 | 0.13911 |
| 319.5 | 0.45933 | 0.30742 | 0.17119 | 0.1375 |
| 320 | 0.45939 | 0.30666 | 0.17309 | 0.13911 |
| 320.5 | 0.46441 | 0.31421 | 0.17813 | 0.1427 |
| 321 | 0.46424 | 0.31503 | 0.176 | 0.14028 |
| 321.5 | 0.46799 | 0.31478 | 0.17404 | 0.14059 |
| 322 | 0.47237 | 0.31656 | 0.17608 | 0.14367 |
| 322.5 | 0.47359 | 0.31501 | 0.17949 | 0.14457 |
| 323 | 0.47451 | 0.31677 | 0.17656 | 0.1419 |
| 323.5 | 0.47376 | 0.31636 | 0.17797 | 0.14586 |
| 324 | 0.47436 | 0.31854 | 0.17661 | 0.14666 |
| 324.5 | 0.47666 | 0.3198 | 0.17978 | 0.14384 |
| 325 | 0.47903 | 0.32144 | 0.18029 | 0.14899 |
| 325.5 | 0.47529 | 0.32167 | 0.17842 | 0.14547 |
| 326 | 0.47688 | 0.31861 | 0.17473 | 0.14548 |
| 326.5 | 0.47703 | 0.31559 | 0.1791 | 0.14523 |
| 327 | 0.47305 | 0.31978 | 0.1776 | 0.14584 |
| 327.5 | 0.47758 | 0.31777 | 0.17513 | 0.14289 |
| 328 | 0.47104 | 0.31552 | 0.17913 | 0.141 |
| 328.5 | 0.47446 | 0.31531 | 0.17372 | 0.14555 |
| 329 | 0.47394 | 0.31703 | 0.17242 | 0.14497 |
| 329.5 | 0.47049 | 0.31108 | 0.17265 | 0.14247 |
| 330 | 0.4664 | 0.30897 | 0.1726 | 0.14408 |
| 330.5 | 0.4627 | 0.30712 | 0.1718 | 0.14277 |
| 331 | 0.4619 | 0.30887 | 0.16869 | 0.14059 |
| 331.5 | 0.45779 | 0.30164 | 0.16805 | 0.14044 |
| 332 | 0.45463 | 0.30454 | 0.17062 | 0.142 |
| 332.5 | 0.44728 | 0.30239 | 0.16498 | 0.13555 |
| 333 | 0.44556 | 0.29585 | 0.16589 | 0.13444 |
| 333.5 | 0.44495 | 0.29724 | 0.16759 | 0.13515 |
| 334 | 0.43594 | 0.29432 | 0.16129 | 0.1349 |
| 334.5 | 0.43232 | 0.29042 | 0.16259 | 0.13365 |
| 335 | 0.43331 | 0.28518 | 0.15988 | 0.13294 |
| 335.5 | 0.42367 | 0.28252 | 0.15701 | 0.13034 |
| 336 | 0.42027 | 0.27797 | 0.15618 | 0.12782 |
| 336.5 | 0.41331 | 0.27468 | 0.15354 | 0.12742 |
| 337 | 0.40769 | 0.27224 | 0.14788 | 0.12529 |
| 337.5 | 0.40077 | 0.26742 | 0.14828 | 0.11975 |
| 338 | 0.39564 | 0.26293 | 0.14713 | 0.11928 |
| 338.5 | 0.38859 | 0.25753 | 0.14218 | 0.118 |
| 339 | 0.38345 | 0.25433 | 0.14114 | 0.11598 |
| 339.5 | 0.37126 | 0.25014 | 0.13832 | 0.11459 |
| 340 | 0.37018 | 0.24662 | 0.13459 | 0.11119 |
| 340.5 | 0.36176 | 0.24034 | 0.12962 | 0.11037 |
| 341 | 0.35465 | 0.23574 | 0.13184 | 0.10804 |
| 341.5 | 0.34878 | 0.23182 | 0.13064 | 0.10607 |
| 342 | 0.3415 | 0.2267 | 0.12583 | 0.10421 |
| 342.5 | 0.33552 | 0.22332 | 0.12442 | 0.10359 |
| 343 | 0.32794 | 0.21836 | 0.12159 | 0.09964 |
| 343.5 | 0.31895 | 0.21516 | 0.11656 | 0.09815 |
| 344 | 0.31387 | 0.20885 | 0.11675 | 0.09592 |
| 344.5 | 0.30224 | 0.19913 | 0.112 | 0.09225 |
| 345 | 0.29394 | 0.1954 | 0.10898 | 0.09096 |
| 345.5 | 0.28351 | 0.19008 | 0.1071 | 0.08732 |
| 346 | 0.2792 | 0.1849 | 0.10239 | 0.08642 |
| 346.5 | 0.27072 | 0.18097 | 0.10173 | 0.08298 |
| 347 | 0.26456 | 0.17373 | 0.09913 | 0.08302 |
| 347.5 | 0.25341 | 0.16797 | 0.09343 | 0.07666 |
| 348 | 0.24812 | 0.16388 | 0.08927 | 0.07845 |
| 348.5 | 0.24 | 0.16043 | 0.08863 | 0.07391 |
| 349 | 0.23068 | 0.15557 | 0.08735 | 0.07104 |
| 349.5 | 0.22372 | 0.14986 | 0.08354 | 0.06816 |
| 350 | 0.21691 | 0.14512 | 0.0828 | 0.06906 |
| 350.5 | 0.20741 | 0.13908 | 0.07697 | 0.06376 |
| 351 | 0.20017 | 0.13613 | 0.07319 | 0.06008 |
| 351.5 | 0.19495 | 0.13056 | 0.07129 | 0.05833 |
| 352 | 0.18717 | 0.12429 | 0.07037 | 0.05798 |
| 352.5 | 0.17818 | 0.11791 | 0.0654 | 0.05432 |
| 353 | 0.17216 | 0.11424 | 0.06528 | 0.05302 |
| 353.5 | 0.16446 | 0.11125 | 0.06225 | 0.05152 |
| 354 | 0.15679 | 0.10614 | 0.0599 | 0.04854 |
| 354.5 | 0.15145 | 0.10316 | 0.05648 | 0.04722 |
| 355 | 0.14362 | 0.09623 | 0.05761 | 0.04582 |
| 355.5 | 0.13648 | 0.09287 | 0.05398 | 0.0432 |
| 356 | 0.13051 | 0.089 | 0.05054 | 0.04233 |
| 356.5 | 0.1258 | 0.08488 | 0.0495 | 0.03941 |
| 357 | 0.11626 | 0.08073 | 0.0457 | 0.03822 |
| 357.5 | 0.11051 | 0.07576 | 0.04152 | 0.0361 |
| 358 | 0.10625 | 0.07303 | 0.04255 | 0.03222 |
| 358.5 | 0.10172 | 0.06728 | 0.03894 | 0.03226 |
| 359 | 0.09468 | 0.06303 | 0.03636 | 0.02886 |
| 359.5 | 0.09169 | 0.06084 | 0.03535 | 0.0284 |
| 360 | 0.08401 | 0.05674 | 0.03356 | 0.02722 |
| 360.5 | 0.0788 | 0.05382 | 0.03276 | 0.02593 |
| 361 | 0.07383 | 0.05114 | 0.02877 | 0.02717 |
| 361.5 | 0.07141 | 0.04689 | 0.02676 | 0.02471 |
| 362 | 0.06722 | 0.04436 | 0.02612 | 0.02203 |
| 362.5 | 0.06281 | 0.04212 | 0.02556 | 0.02224 |
| 363 | 0.06088 | 0.03848 | 0.02486 | 0.02006 |
| 363.5 | 0.05445 | 0.03819 | 0.02156 | 0.01898 |
| 364 | 0.05047 | 0.03482 | 0.02148 | 0.01554 |
| 364.5 | 0.05055 | 0.0308 | 0.02012 | 0.01548 |
| 365 | 0.04506 | 0.02872 | 0.01801 | 0.01459 |
| 365.5 | 0.0411 | 0.0262 | 0.01588 | 0.01378 |
| 366 | 0.04004 | 0.02547 | 0.01524 | 0.01502 |
| 366.5 | 0.0372 | 0.02295 | 0.01525 | 0.01223 |
| 367 | 0.03426 | 0.02278 | 0.01423 | 0.01064 |
| 367.5 | 0.03218 | 0.01963 | 0.01172 | 0.00944 |
| 368 | 0.02858 | 0.01908 | 0.0089 | 0.00844 |
| 368.5 | 0.02619 | 0.01808 | 0.00935 | 0.00791 |
| 369 | 0.0239 | 0.01627 | 0.01096 | 0.00939 |
| 369.5 | 0.02167 | 0.01351 | 0.0083 | 0.00863 |
| 370 | 0.01892 | 0.01337 | 0.00811 | 0.00754 |
| 370.5 | 0.01895 | 0.01075 | 0.00749 | 0.00655 |
| 371 | 0.01687 | 0.0088 | 0.00702 | 0.00753 |
| 371.5 | 0.01467 | 0.00789 | 0.00651 | 0.00606 |
| 372 | 0.01375 | 0.007 | 0.00742 | 0.00615 |
| 372.5 | 0.0143 | 0.00853 | 0.00631 | 0.00378 |
| 373 | 0.01054 | 0.00827 | 0.00429 | 0.00308 |
| 373.5 | 0.00795 | 0.00659 | 0.00509 | 0.00314 |
| 374 | 0.00776 | 0.00404 | 0.00412 | 0.00319 |
| 374.5 | 0.00662 | 0.0031 | 0.0042 | 0.00461 |
| 375 | 0.007 | 0.00331 | 0.00444 | 0.00448 |
| 375.5 | 0.00605 | 0.00277 | 0.00188 | 0.00294 |
| 376 | 0.00688 | 0.0029 | 0.00221 | 0.00265 |
| 376.5 | 0.00415 | 0.00261 | 0.00283 | 0.00153 |
| 377 | 0.00532 | 0.00301 | 0.00052 | 0.00234 |
| 377.5 | 0.00265 | 0.00032 | 0.0028 | 0.00189 |
| 378 | 0.00301 | -0.00071 | 0.00164 | 0.00108 |
| 378.5 | 0.00256 | 0.00079 | 0.00277 | 0.00172 |
| 379 | 0.00021 | 2e-05 | 0.00172 | 0.00167 |
| 379.5 | 0.00115 | -0.00035 | 0.00184 | 0.00175 |
| 380 | 0 | 0 | 0 | 0 |
| 380.5 | -0.00077 | -0.00095 | -0.00029 | 0.00061 |
| 381 | -0.00127 | -0.00022 | 0.0008 | 0.00028 |
| 381.5 | -0.00162 | -0.00191 | -0.00103 | 0.00028 |
| 382 | -0.0017 | -0.00121 | -0.00012 | 0.00069 |
| 382.5 | -0.00034 | -0.00248 | 0.00041 | -0.00112 |
| 383 | -0.00193 | -0.00209 | 0.00107 | 0.0003 |
| 383.5 | -0.00183 | -0.00189 | 0.00015 | -0.00024 |
| 384 | -0.00186 | -0.00251 | -0.00055 | 0.00076 |
| 384.5 | -0.00283 | -0.00167 | -0.00278 | 0.00051 |
| 385 | -0.00298 | -0.00094 | 0.00133 | 0.00041 |
| 385.5 | -0.0045 | -0.00326 | 0.00072 | -0.00075 |
| 386 | -0.00401 | -0.00277 | -0.00085 | -0.00033 |
| 386.5 | -0.00465 | -0.00277 | 0.0011 | 0.00218 |
| 387 | -0.00435 | -0.00316 | -0.00084 | 0.00058 |
| 387.5 | -0.00211 | -0.00276 | -0.00147 | 0.00174 |
| 388 | -0.00287 | -0.0034 | -0.00143 | 0.00027 |
| 388.5 | -0.00118 | -0.00365 | 0.00066 | -0.0002 |
| 389 | -0.00299 | -0.00291 | -0.00204 | -0.00035 |
| 389.5 | -0.00511 | -0.00396 | -0.00104 | 0.00012 |

## Hansen Solubility Parameters (HSP)

HSP was initially devised as a chemical tool to predict the solubility of one chemical species with another and whether a solution will be formed.^[1]^ HSP relates the interaction ability of molecules with other species through three primary parameters, known as cohesion energy parameters:

1. δ_d_ – the energy from dispersion forces between molecules,
2. δ_p_ – the energy from dipolar intermolecular forces between molecules,
3. δ_h_ – the energy from hydrogen bonds between the molecules.

**Table S1** The hydrogen bonding ability of six commonly available solvents as reported in literature.^[2]^

| **Solvent** | δ**_h_ (cal mL^-1^)** |
| --- | --- |
| Acetone | 3.4 |
| Acetonitrile | 3.0 |
| *N,N*-dimethylformamide | 5.5 |
| 1,4-dioxane | 3.6 |
| Tetrahydrofuran | 3.9 |
| Toluene | 1.0 |

## Gutmann-Beckett method

To quantify solvent Lewis acidity, the Gutmann-Beckett method was used to give a quantifiable metric of a solvent’s donor number (Lewis basic character) and acceptor number (Lewis acidic character). ^31^P NMR spectroscopy was employed to monitor the chemical shift of the phosphorus nucleus in triethyl phosphine oxide (Et_3_PO) when dissolved in deuterated solvents like acetonitrile and toluene.^[3],^^[4]^ The extent of the downfield shift reflects the Lewis acidity of the solvent. Et_3_PO is an ideal spin probe due to its high solubility in a wide range of solvents and its ethyl substituents, which offer electronic shielding without steric hindrance. The acceptor number (AN) is derived from the ^31^P NMR chemical shift (δ) of Et_3_PO in a given solvent. The scale is anchored by two reference points: hexane (δ = 41 ppm, AN = 0) and SbCl_5_ (δ = 86 ppm, AN = 100). The acceptor number is calculated using the following linear equation:

Acceptor number = 2.21 (δ_ppm_ – 41)	Equation 1

Where δ_solvent_ is the ^31^P chemical shift of Et_3_PO (ppm) in the solvent of interest, and 41 ppm is the reference shift in hexane.

**Table S2** Phosphorus peak value of Et_3_PO spin probe measured in a 300 MHz Bruker NMR instrument in deuterated acetonitrile and toluene solutions, respectively.

| **Solvent** | **δ_ppm_** | **Acceptor number** |
| --- | --- | --- |
| Toluene | 46.6 | 12.4 |
| Dichloromethane | 50.8 | 21.7 |

As the acceptor number measured for the reference dichloromethane solvent is slightly higher than the 20.1 stated in literature, we acknowledge an overestimation by +2 for toluene’s acceptor number.

**Figure S3** Et_3_PO spin probe in a deuterated toluene medium.

**Figure S4** Et_3_PO spin probe in a deuterated dichloromethane medium.

The acceptor and donor numbers in the screening of commonly available laboratory solvents are shown in Table S3. The donor number value indicates the degree of nucleophilicity of the solvent, usually determined experimentally through calorimetry. It is from the heat of reaction between the solvent dissolved in dichloromethane and SbCl_5_. Indirect methods are needed for protic solvents due to the decomposition of SbCl_5_. The difference between these values suggests the solvent acts as a nucleophile (Lewis base) or an electrophile (Lewis acid).

**Table S3** Acceptor and donor numbers of six commonly available solvents as reported in literature.^2^ Measured acceptor number of toluene.*

| **Solvent** | **Acceptor number (AN)** | **Donor number (DN)** |
| --- | --- | --- |
| Acetone | 12.5 | 17.0 |
| Acetonitrile | 18.9 | 14.1 |
| *N,N*-dimethylformamide | 16.0 | 26.6 |
| 1,4-dioxane | 10.3 | 14.3 |
| Tetrahydrofuran | 8.0 | 20.0 |
| Toluene | 10.4* | 0.1 |

## Catalytic rates in different solvents

The catalytic reactions were performed using the following protocol. **NBD1** solutions of a 4.48 × 10^–3^ M concentration of 10 mL were prepared in DCM, DMF, THF, 1,4-dioxane, acetone, acetonitrile, and toluene. The **NBD1** solution was irradiated using a UVA (315–400 nm) light source until less than 2% of **NBD1** remained in solution. The catalytic reaction is started using a 10% weight ratio of Pt/C catalyst (1 mg) and stirred at a rate of 500 rpm, and monitored at specific time intervals. A 1 in 100 dilution in the respective solvent and catalyst separation from the reaction mixture using 0.22 μm filters were done prior to quantification via UV–vis measurements. The isomerization reaction is quantified by the formation of **NBD1** using a Shimadzu UV–vis spectrometer. The absorbance value at 340 nm is taken as no overlap occurs with the absorption band of **QC1**, and has a high linearity in the concentration range of the UV-Vis instrument.

ln[QC]

| Time (minutes) | DMF | THF | 1,4-dioxane | Acetone | Acetonitrile |
| --- | --- | --- | --- | --- | --- |
| 0 | -10.1996 | -10.1996 | -10.2203 | -10.2099 | -10.2067 |
| 5 | -10.1997 | -10.2001 | -10.2435 | -10.2663 | -10.3532 |
| 10 | -10.1996 | -10.2002 | -10.2646 | -10.291 | -10.4601 |
| 30 | -10.1999 | -10.2005 | -10.3411 | -10.3813 | -10.793 |

| Time (minutes) | Toluene |
| --- | --- |
| 0 | -10.2175 |
| 2 | -10.6555 |
| 5 | -10.704 |
| 10 | -11.2214 |
| 30 | -11.9266 |

**Figure S5** Catalytic rates of reaction of **QC1** to **NBD1** using a 10 wt% of Pt/C catalyst in different solvent media.

**Table S4** Rate constants of the QC1 to NBD1 reaction using a 10 wt% of Pt/C catalyst in different solvent media.

| **Solvent** | **Rate constant (*k*, s^-1^)** |
| --- | --- |
| DMF | 9.21 x 10^-6^ |
| THF | 2.32 x 10^-5^ |
| 1,4-dioxane | 3.98 x 10^-3^ |
| Acetone | 5.32 x 10^-3^ |
| Acetonitrile | 1.88 x 10^-2^ |
| Toluene | 5.18 x 10^-2^ |

## X-ray photoelectron spectroscopy (XPS)

X-ray photoelectron spectroscopy (XPS) spectra were acquired using a PHI Quantera II system equipped with a monochromated, micro-focused, scanning Al Kα X-ray source (hν = 1486.6 eV, 15 kV, 25 W), a hemispherical electron energy analyzer, and a robotic sample handling system. Powder samples were mounted on the sample holder using conductive carbon or copper tapes. For each sample, survey spectra and detailed core-level spectra of selected elements were recorded from two different spots. All spectra were collected at constant pass energy and at a photoelectron emission angle of 0° relative to the sample surface normal. The instrumental energy resolution, determined from the full width at half maximum (FWHM) of the Ag 3d peak, was 0.48 eV. The lateral spatial resolution of the analysis was approximately 7.5 µm.

Data analysis and peak fitting were performed using KolXPD software.^[5]^ The Pt 4f core level spectra were fitted using asymmetric Doniach–Šunjić line shapes convoluted with a Gaussian function. The asymmetry parameter was applied for metallic Pt species and fixed to zero for oxidized species. The core level spectra of the remaining chemical elements were fitted using Voigt profile. A combined linear and Shirley background was subtracted from all spectra prior to fitting.

**Figure S6** Pt 4f XPS spectrum showing the overall fit (red), deconvoluted Pt²⁺ (blue) and Pt⁴⁺ (green) components. Spin–orbit splitting and area ratios were constrained according to the Pt 4f doublet structure.

**Figure S7** High‑resolution C 1s XPS spectrum showing the overall fit (red) and deconvoluted components corresponding to C–C (C1), C–H (C2), and C=O (C3).

**Figure S8** High‑resolution O 1s XPS spectrum showing the overall fit (red) and deconvoluted components assigned to C=O (O1) and C-O (O2).

# 2 Green solvent screening

## Physical and chemical properties of green solvents

**Table S5** Physical properties of the three environmentally friendly aprotic solvents.^[6]^

| **Solvent** | **Flash point (°C)** | **Heat capacity (J g^-1^ K^-1^)** | **Melting point (°C)** | **Thermal conductivity (W m^-1^ K^-1^)** | **Dynamic viscosity @ 20 °C (cP)** | **Boiling point °C** |
| --- | --- | --- | --- | --- | --- | --- |
| Cyrene | 108 | 1.51 | -18 | 0.18 | 14.5 | 227 |
| Propylene carbonate | 134 | 1.64 | -49 | 0.16 | 2.76 | 242 |
| Sulfolane | 165 | 1.92 | 27 | 0.17 | 10.3 | 285 |

**Table S6** Gutmann acceptor-donor numbers and the hydrogen bonding ability of the three green solvents.^[7],^^[8],^^[9]^

| **Solvent** | **Acceptor number (AN)** | **Donor number (DN)** | **δ_h_ (cal mL^-1^)^1/2^** |
| --- | --- | --- | --- |
| Cyrene | - | - | 3.37 |
| Propylene carbonate | 18.3 | 15.1 | 2.0 |
| Sulfolane | 19.2 | 14.8 | 4.8 |

Upon analysis, whilst Propylene carbonate, acetonitrile, and toluene exhibit varying degrees of Lewis acidity, Propylene carbonate exhibits a hydrogen bonding parameter of 2.0, which lies between those of acetonitrile and toluene. Its catalytic conversion also falls between these two reference solvents, suggesting a weaker coordination of Propylene carbonate solvent molecules to the metal centre compared to acetonitrile. Cyrene has a hydrogen bonding capacity comparable to that of acetone, which shows a similar performance; however, its acceptor number (AN) and donor number (DN) have not been reported, limiting further interpretation. In the case of Sul, its hydrogen bonding ability is intermediate between that of THF and DMF. Despite exhibiting greater electrophilicity than those solvents, its performance aligns more closely with that of THF. To finalise, the higher acceptor number with respect to the equivalent donor number, so a greater Lewis acidic character improves the overall reaction rate. The lower hydrogen bonding ability, preferably being a hydrogen bond acceptor rather than hydrogen bond donor is also preferred.

**Table S7** Comparison of green solvent MOST systems.

| **Photoswitch** | **Solvent** | **Energy storage enthalpy (kJ/mol)** | **Thermal half-life** | **Solubility (M)** | **Temperature increase (°C)** |
| --- | --- | --- | --- | --- | --- |
| ^10^ | Water | 65.2 | 587 hours at 371 K | N/A | N/A |
| ^11^ | Water | 70.5 | 61 days at 298 K | 0.0054 | N/A |
| ^12^ | Water | 69.5 | 1.9 years at 298 K | 1.64 | 7.5 °C |
| ^13^ | Water/Triton X-100 surfactant | N/A | 112 days | 1.6 | 4.7 °C |
| ^14^  | Water | 227.6 | 481 days at 298 K | Solid | 100 ˚C |

## Isomerisation reactions monitored by UPLC

10 mg of **NBD1** was dissolved in 10 mL of each green solvent (Cyrene, Sul, and Propylene carbonate) and irradiated for 1 hour under UVA light (315 – 400 nm). Full conversion to the **QC1** state was achieved in all three solvents. Subsequently, 1 mg (10 wt%) of Pt/C catalyst was added to each solution to initiate the back-reaction and reacted for 1 hour. All UPLC measurements were conducted using a ratio of 20 µL reaction sample and a dilution up to 1 mL of acetonitrile. Each measurement sample was filtered using a 0.45 µm PTFE filter prior to injection. The solution is injected into a C18 hydrophobic column, which separates the structural isomers NBD and QC using an initial ratio of 100:0 acetonitrile: water, whilst after 30 minutes, the final ratio is 70:30 acetonitrile: water. A flow rate of 1 mL/minute is applied, whereby a wavelength of 340 nm (detecting NBD species only – no overlap) was used to quantify the concentration of **NBD1** in the samples. Another wavelength of 290 nm (detecting QC species predominantly) was used to monitor the species present in the samples at different stages of the reaction.

**Figure S9** UPLC measurements of the back conversion of **QC1** to **NBD1** in Cyrene**. NBD1** with a retention time of 6.97 minutes (top), **NBD1** irradiated using a UVA light source (middle), and **NBD1** produced after 1 hour of catalytic reaction. All reactions were performed in a Cyrene solution and recorded using a wavelength of 340 nm.

**Figure S10** UPLC measurements of the back conversion of **QC1** to **NBD1** in sulfolane. **NBD1** with a retention time of 6.96 minutes (top), **NBD1** irradiated using a UVA light source (middle), and **NBD1** produced after 1 hour of catalytic reaction. All reactions were performed in a sulfolane solution and recorded using a wavelength of 340 nm.

**Figure S11** UPLC measurements of the backconversion of **QC1** to **NBD1** in propylene carbonate. **NBD1** with a retention time of 6.98 minutes (top), **NBD1** irradiated using a UVA light source (middle), and **NBD1** produced after 1 hour of catalytic reaction. All reactions were performed in a propylene carbonate solution and recorded using a wavelength of 340 nm.

**Figure S12** **NBD1** peak area in Cyrene.

**Figure S13 NBD1** peak area in Cyrene after one hour of UVA irradiation.

**Figure S14 NBD1** peak area in Cyrene after 1 hour of catalytic reaction.

**Figure S15 NBD1** peak area in sulfolane.

**Figure S16 NBD1** peak area in sulfolane after one hour of UVA irradiation.

**Figure S17 NBD1** peak area in sulfolane after 1 hour of catalytic reaction.

**Figure S18 NBD1** peak area in propylene carbonate.

**Figure S19 NBD1** peak area in propylene carbonate after one hour of UVA irradiation.

**Figure S20 NBD1** peak area in propylene carbonate after 1 hour of catalytic reaction.

**Table S8** The NBD peak areas and conversions (retention time – 6.96 minutes) before and after the photoisomerisation and catalytic reactions of NBD-QC photoswitch in three different green solvents.

| **Solvent** | **Start** | **After photoisomerisation** | **After catalytic reaction** | **Photoisomerisation %** | **Catalytic conversion %** |
| --- | --- | --- | --- | --- | --- |
| Cyrene | 546.54 | 20.87 | 105.33 | 3.81 | 19.27 |
| Sulfolane | 475.40 | 15.28 | 30.22 | 3.21 | 6.36 |
| Propylene carbonate | 483.07 | 12.64 | 311.90 | 2.62 | 64.57 |

The chromatograms at wavelength 290 nm were recorded to witness the photoisomerisation to the QC state in each respective solvent. Moreover, to verify that NBD is being quantified solely and that the NBD and QC species are separated effectively using the applied method. In UPLC, separation of solutes is in the reverse-phase, whereby more polar solutes are eluted first. The structural isomeric peak belonging to the QC species appears at 6.5 minutes after photoisomerisation for each of the reactions. An additional major peak with a retention time of 4.7 minutes absorbs strongly at 290 nm, appearing in all the catalytic reactions. Thus, a run of only the dihydrogen hexachloroplatinate (IV) hexahydrate precursor was conducted to confirm that the peak belongs to the active species on the Pt/C catalyst.

**Figure S21** UPLC measurements of the backconversion of **QC1** to **NBD1** in Cyrene**. NBD1** peak with a retention time of 7.0 minutes at 290 nm wavelength (top), **NBD1** irradiated using a UVA light source, peak at 6.5 minutes attributed to **QC1** (middle), **NBD1** produced after 1 hour of catalytic reaction (bottom). All reactions were performed in a Cyrene solution.

**Figure S22** UPLC measurements of the backconversion of **QC1** to **NBD1** in sulfolane. **NBD1** peak with a retention time of 7.0 minutes at 290 nm wavelength (top), **NBD1** irradiated using a UVA light source, peak at 6.5 minutes attributed to **QC1** (middle), **NBD1** produced after 1 hour of catalytic reaction (bottom). All reactions were performed in a sulfolane solution.

**Figure S23** UPLC measurements of the backconversion of **QC1** to **NBD1** in propylene carbonate. **NBD1** peak with a retention time of 7.0 minutes at 290 nm wavelength (top), **NBD1** irradiated using a UVA light source, peak at 6.5 minutes attributed to **QC1** (middle), **NBD1** produced after 1 hour of catalytic reaction (bottom). All reactions were performed in a propylene carbonate solution.

**Figure S24** UPLC run of H_2_PtCl_6_.6H_2_O species diluted in acetonitrile.

## UV-Vis spectra

Absorbance (a.u)

| Wavelength (nm) | cyrene | sulfolane |
| --- | --- | --- |
| 250 | 4.8616 | 3.98935 |
| 251 | 4.7169 | 4.06129 |
| 252 | 4.68174 | 4.18817 |
| 253 | 4.72487 | 4.25471 |
| 254 | 4.60477 | 4.2783 |
| 255 | 4.75893 | 4.29255 |
| 256 | 4.88334 | 4.27387 |
| 257 | 4.76335 | 4.26008 |
| 258 | 4.74523 | 4.33039 |
| 259 | 4.79074 | 4.31523 |
| 260 | 4.87398 | 4.29775 |
| 261 | 4.79379 | 4.28585 |
| 262 | 4.94471 | 4.24427 |
| 263 | 4.8203 | 4.30129 |
| 264 | 4.81455 | 4.31703 |
| 265 | 5.04234 | 4.35788 |
| 266 | 4.90194 | 4.39683 |
| 267 | 5.07004 | 4.34131 |
| 268 | 4.97535 | 4.35487 |
| 269 | 4.91037 | 4.27951 |
| 270 | 4.9996 | 4.32716 |
| 271 | 5.07841 | 4.27852 |
| 272 | 5.17684 | 4.25953 |
| 273 | 4.96248 | 4.23024 |
| 274 | 4.99848 | 4.19826 |
| 275 | 4.99446 | 4.16378 |
| 276 | 5.02083 | 4.14133 |
| 277 | 5.34969 | 4.18843 |
| 278 | 5.28323 | 4.21526 |
| 279 | 5.13547 | 4.2941 |
| 280 | 5.02035 | 4.31396 |
| 281 | 5.00768 | 4.32283 |
| 282 | 5.15783 | 4.30115 |
| 283 | 4.94845 | 4.30724 |
| 284 | 4.92032 | 4.30231 |
| 285 | 4.8204 | 4.26166 |
| 286 | 4.77445 | 4.26487 |
| 287 | 4.77759 | 4.20457 |
| 288 | 4.67543 | 4.23606 |
| 289 | 4.75853 | 4.23042 |
| 290 | 4.91083 | 4.29908 |
| 291 | 4.95534 | 4.39406 |
| 292 | 4.8297 | 4.38967 |
| 293 | 5.03774 | 4.49834 |
| 294 | 5.26906 | 4.48191 |
| 295 | 5.14857 | 4.42607 |
| 296 | 5.16395 | 4.30548 |
| 297 | 5.11289 | 4.32117 |
| 298 | 4.67741 | 4.35601 |
| 299 | 4.45448 | 4.4462 |
| 300 | 4.33514 | 4.47218 |
| 301 | 4.26218 | 4.44889 |
| 302 | 4.22264 | 4.36843 |
| 303 | 4.24114 | 4.2009 |
| 304 | 4.19018 | 4.28296 |
| 305 | 4.26604 | 4.31641 |
| 306 | 4.3744 | 4.39561 |
| 307 | 4.30216 | 4.54963 |
| 308 | 4.31978 | 4.46832 |
| 309 | 4.26452 | 4.34648 |
| 310 | 4.22555 | 4.29622 |
| 311 | 4.28152 | 4.32398 |
| 312 | 4.26751 | 4.31517 |
| 313 | 4.28684 | 4.23498 |
| 314 | 4.30754 | 4.20077 |
| 315 | 4.2359 | 4.17159 |
| 316 | 4.22128 | 4.16065 |
| 317 | 4.21531 | 4.14154 |
| 318 | 4.19287 | 4.13094 |
| 319 | 4.13131 | 4.13763 |
| 320 | 4.15374 | 4.08447 |
| 321 | 4.12324 | 4.0912 |
| 322 | 4.16701 | 4.0671 |
| 323 | 4.18837 | 4.07749 |
| 324 | 4.14608 | 4.10637 |
| 325 | 4.1737 | 4.08231 |
| 326 | 4.0719 | 4.0974 |
| 327 | 4.09545 | 4.09335 |
| 328 | 4.08776 | 4.04537 |
| 329 | 4.06118 | 4.02191 |
| 330 | 4.08417 | 4.01045 |
| 331 | 4.07753 | 3.97681 |
| 332 | 4.04216 | 3.95664 |
| 333 | 4.02637 | 3.94634 |
| 334 | 4.02074 | 3.95642 |
| 335 | 3.9784 | 3.9873 |
| 336 | 3.97257 | 3.95529 |
| 337 | 3.93694 | 3.98239 |
| 338 | 3.91183 | 3.9585 |
| 339 | 3.91772 | 3.94516 |
| 340 | 3.89373 | 3.9846 |
| 341 | 3.8727 | 3.95193 |
| 342 | 3.86395 | 3.95312 |
| 343 | 3.83709 | 3.94089 |
| 344 | 3.84009 | 3.92377 |
| 345 | 3.82713 | 3.91314 |
| 346 | 3.84419 | 3.94123 |
| 347 | 3.87583 | 3.93348 |
| 348 | 3.8486 | 3.92294 |
| 349 | 3.81443 | 3.92634 |
| 350 | 3.76458 | 3.95611 |
| 351 | 3.6949 | 3.90814 |
| 352 | 3.64976 | 3.91016 |
| 353 | 3.57569 | 3.88691 |
| 354 | 3.47033 | 3.8804 |
| 355 | 3.36726 | 3.88158 |
| 356 | 3.25063 | 3.91402 |
| 357 | 3.14101 | 3.93421 |
| 358 | 3.03717 | 3.91415 |
| 359 | 2.90054 | 3.88112 |
| 360 | 2.78207 | 3.83256 |
| 361 | 2.67501 | 3.78544 |
| 362 | 2.5736 | 3.73179 |
| 363 | 2.49189 | 3.67996 |
| 364 | 2.41457 | 3.58935 |
| 365 | 2.33764 | 3.49003 |
| 366 | 2.26202 | 3.36662 |
| 367 | 2.18752 | 3.22764 |
| 368 | 2.11265 | 3.07405 |
| 369 | 2.03879 | 2.91345 |
| 370 | 1.9671 | 2.75355 |
| 371 | 1.89447 | 2.58828 |
| 372 | 1.82313 | 2.42804 |
| 373 | 1.75241 | 2.26975 |
| 374 | 1.68249 | 2.11707 |
| 375 | 1.61513 | 1.97169 |
| 376 | 1.54984 | 1.83185 |
| 377 | 1.48581 | 1.69811 |
| 378 | 1.42314 | 1.5712 |
| 379 | 1.3635 | 1.45339 |
| 380 | 1.30589 | 1.34606 |
| 381 | 1.2512 | 1.24805 |
| 382 | 1.19919 | 1.15844 |
| 383 | 1.1481 | 1.0767 |
| 384 | 1.0979 | 1.00096 |
| 385 | 1.04831 | 0.93164 |
| 386 | 0.9995 | 0.86924 |
| 387 | 0.95193 | 0.81393 |
| 388 | 0.90601 | 0.76487 |
| 389 | 0.86114 | 0.72212 |
| 390 | 0.81775 | 0.68491 |
| 391 | 0.77612 | 0.65195 |
| 392 | 0.73515 | 0.62284 |
| 393 | 0.69485 | 0.59692 |
| 394 | 0.65523 | 0.57372 |
| 395 | 0.61636 | 0.55283 |
| 396 | 0.57923 | 0.53398 |
| 397 | 0.54424 | 0.51709 |
| 398 | 0.51121 | 0.50169 |
| 399 | 0.48041 | 0.48769 |
| 400 | 0.45202 | 0.47486 |
| 401 | 0.42547 | 0.46303 |
| 402 | 0.401 | 0.45175 |
| 403 | 0.37861 | 0.44162 |
| 404 | 0.35781 | 0.43175 |
| 405 | 0.33902 | 0.42239 |
| 406 | 0.32186 | 0.41365 |
| 407 | 0.30632 | 0.40518 |
| 408 | 0.29245 | 0.3974 |
| 409 | 0.27998 | 0.38999 |
| 410 | 0.26873 | 0.3829 |
| 411 | 0.25866 | 0.37614 |
| 412 | 0.24955 | 0.36954 |
| 413 | 0.2411 | 0.36332 |
| 414 | 0.23351 | 0.35735 |
| 415 | 0.22648 | 0.35167 |
| 416 | 0.2201 | 0.34627 |
| 417 | 0.2142 | 0.34099 |
| 418 | 0.2089 | 0.33603 |
| 419 | 0.20373 | 0.33138 |
| 420 | 0.19892 | 0.32677 |
| 421 | 0.19436 | 0.32258 |
| 422 | 0.19002 | 0.31856 |
| 423 | 0.18608 | 0.31459 |
| 424 | 0.18239 | 0.31102 |
| 425 | 0.17891 | 0.30748 |
| 426 | 0.17553 | 0.30404 |
| 427 | 0.17224 | 0.30086 |
| 428 | 0.16908 | 0.29788 |
| 429 | 0.166 | 0.29507 |
| 430 | 0.16312 | 0.29236 |
| 431 | 0.16036 | 0.2898 |
| 432 | 0.15769 | 0.28723 |
| 433 | 0.15507 | 0.28474 |
| 434 | 0.15246 | 0.28232 |
| 435 | 0.14985 | 0.27994 |
| 436 | 0.14729 | 0.27765 |
| 437 | 0.14472 | 0.27533 |
| 438 | 0.14225 | 0.27323 |
| 439 | 0.13981 | 0.27121 |
| 440 | 0.13741 | 0.26924 |
| 441 | 0.13497 | 0.2672 |
| 442 | 0.13247 | 0.26516 |
| 443 | 0.12995 | 0.26313 |
| 444 | 0.12739 | 0.26111 |
| 445 | 0.12481 | 0.25918 |
| 446 | 0.12227 | 0.2574 |
| 447 | 0.11973 | 0.25569 |
| 448 | 0.11716 | 0.25398 |
| 449 | 0.11455 | 0.25245 |

| Wavelength (nm) | propylene carbonate |
| --- | --- |
| 200 | 5.10315 |
| 201 | 5.12878 |
| 202 | 4.84286 |
| 203 | 4.74303 |
| 204 | 4.66589 |
| 205 | 4.69822 |
| 206 | 4.52143 |
| 207 | 4.38448 |
| 208 | 4.10845 |
| 209 | 3.88229 |
| 210 | 3.67799 |
| 211 | 3.5015 |
| 212 | 3.3489 |
| 213 | 3.22501 |
| 214 | 3.11751 |
| 215 | 3.0387 |
| 216 | 2.95469 |
| 217 | 2.88723 |
| 218 | 2.82359 |
| 219 | 2.75348 |
| 220 | 2.67936 |
| 221 | 2.5964 |
| 222 | 2.50408 |
| 223 | 2.40939 |
| 224 | 2.31473 |
| 225 | 2.22027 |
| 226 | 2.13089 |
| 227 | 2.04168 |
| 228 | 1.95619 |
| 229 | 1.87226 |
| 230 | 1.78974 |
| 231 | 1.70577 |
| 232 | 1.62037 |
| 233 | 1.53309 |
| 234 | 1.44424 |
| 235 | 1.35335 |
| 236 | 1.26195 |
| 237 | 1.17195 |
| 238 | 1.08423 |
| 239 | 1.00107 |
| 240 | 0.92241 |
| 241 | 0.84875 |
| 242 | 0.77964 |
| 243 | 0.71604 |
| 244 | 0.65758 |
| 245 | 0.60508 |
| 246 | 0.55946 |
| 247 | 0.5204 |
| 248 | 0.48735 |
| 249 | 0.45996 |
| 250 | 0.43749 |
| 251 | 0.41883 |
| 252 | 0.40422 |
| 253 | 0.39256 |
| 254 | 0.38328 |
| 255 | 0.37614 |
| 256 | 0.3708 |
| 257 | 0.36646 |
| 258 | 0.36327 |
| 259 | 0.36072 |
| 260 | 0.35882 |
| 261 | 0.35714 |
| 262 | 0.35565 |
| 263 | 0.35462 |
| 264 | 0.35338 |
| 265 | 0.35214 |
| 266 | 0.35074 |
| 267 | 0.34942 |
| 268 | 0.34785 |
| 269 | 0.34623 |
| 270 | 0.34459 |
| 271 | 0.34282 |
| 272 | 0.34097 |
| 273 | 0.33902 |
| 274 | 0.33666 |
| 275 | 0.33398 |
| 276 | 0.33065 |
| 277 | 0.32683 |
| 278 | 0.32282 |
| 279 | 0.3185 |
| 280 | 0.3141 |
| 281 | 0.30984 |
| 282 | 0.30548 |
| 283 | 0.30089 |
| 284 | 0.29627 |
| 285 | 0.29118 |
| 286 | 0.28577 |
| 287 | 0.27982 |
| 288 | 0.27351 |
| 289 | 0.26687 |
| 290 | 0.26011 |
| 291 | 0.25311 |
| 292 | 0.24623 |
| 293 | 0.23952 |
| 294 | 0.23289 |
| 295 | 0.22627 |
| 296 | 0.21997 |
| 297 | 0.21366 |
| 298 | 0.20771 |
| 299 | 0.2017 |
| 300 | 0.19583 |
| 301 | 0.19015 |
| 302 | 0.18494 |
| 303 | 0.1798 |
| 304 | 0.17489 |
| 305 | 0.16994 |
| 306 | 0.16482 |
| 307 | 0.15956 |
| 308 | 0.15425 |
| 309 | 0.14916 |
| 310 | 0.14425 |
| 311 | 0.13933 |
| 312 | 0.13446 |
| 313 | 0.1297 |
| 314 | 0.12506 |
| 315 | 0.12065 |
| 316 | 0.11656 |
| 317 | 0.11248 |
| 318 | 0.10891 |
| 319 | 0.10523 |
| 320 | 0.10192 |
| 321 | 0.09898 |
| 322 | 0.09618 |
| 323 | 0.09367 |
| 324 | 0.09146 |
| 325 | 0.08947 |
| 326 | 0.08767 |
| 327 | 0.08597 |
| 328 | 0.08424 |
| 329 | 0.08304 |
| 330 | 0.08157 |
| 331 | 0.08049 |
| 332 | 0.07945 |
| 333 | 0.07851 |
| 334 | 0.07761 |
| 335 | 0.07665 |
| 336 | 0.07566 |
| 337 | 0.07457 |
| 338 | 0.07351 |
| 339 | 0.07248 |
| 340 | 0.07159 |
| 341 | 0.07098 |
| 342 | 0.07025 |
| 343 | 0.06955 |
| 344 | 0.06879 |
| 345 | 0.0679 |
| 346 | 0.06705 |
| 347 | 0.06638 |
| 348 | 0.06576 |
| 349 | 0.06536 |
| 350 | 0.0651 |
| 351 | 0.06455 |
| 352 | 0.06414 |
| 353 | 0.06348 |
| 354 | 0.06281 |
| 355 | 0.06226 |
| 356 | 0.06182 |
| 357 | 0.06142 |
| 358 | 0.0611 |
| 359 | 0.06083 |
| 360 | 0.06042 |
| 361 | 0.06 |
| 362 | 0.05952 |
| 363 | 0.05917 |
| 364 | 0.05878 |
| 365 | 0.05852 |
| 366 | 0.05823 |
| 367 | 0.05787 |
| 368 | 0.05747 |
| 369 | 0.05722 |
| 370 | 0.05682 |
| 371 | 0.05656 |
| 372 | 0.05625 |
| 373 | 0.05598 |
| 374 | 0.05578 |
| 375 | 0.05566 |
| 376 | 0.05537 |
| 377 | 0.05516 |
| 378 | 0.05498 |
| 379 | 0.05464 |
| 380 | 0.05442 |
| 381 | 0.05419 |
| 382 | 0.05404 |
| 383 | 0.05387 |
| 384 | 0.05369 |
| 385 | 0.05357 |
| 386 | 0.05338 |
| 387 | 0.05318 |
| 388 | 0.05309 |
| 389 | 0.05292 |
| 390 | 0.05275 |
| 391 | 0.0526 |
| 392 | 0.05241 |
| 393 | 0.05225 |
| 394 | 0.05212 |
| 395 | 0.05202 |
| 396 | 0.05189 |
| 397 | 0.05181 |
| 398 | 0.05158 |
| 399 | 0.05154 |

| Wavelength (nm) | NBD1 |
| --- | --- |
| 250 | 0.4495 |
| 251 | 0.43947 |
| 252 | 0.42348 |
| 253 | 0.43133 |
| 254 | 0.41694 |
| 255 | 0.42648 |
| 256 | 0.42114 |
| 257 | 0.41977 |
| 258 | 0.41646 |
| 259 | 0.40569 |
| 260 | 0.39353 |
| 261 | 0.40787 |
| 262 | 0.41178 |
| 263 | 0.3992 |
| 264 | 0.39457 |
| 265 | 0.38367 |
| 266 | 0.37245 |
| 267 | 0.36312 |
| 268 | 0.35392 |
| 269 | 0.35136 |
| 270 | 0.34982 |
| 271 | 0.34803 |
| 272 | 0.3491 |
| 273 | 0.3491 |
| 274 | 0.34544 |
| 275 | 0.33641 |
| 276 | 0.33195 |
| 277 | 0.32661 |
| 278 | 0.3244 |
| 279 | 0.32354 |
| 280 | 0.32619 |
| 281 | 0.32927 |
| 282 | 0.33204 |
| 283 | 0.3351 |
| 284 | 0.3381 |
| 285 | 0.34039 |
| 286 | 0.3436 |
| 287 | 0.34681 |
| 288 | 0.35093 |
| 289 | 0.35556 |
| 290 | 0.36087 |
| 291 | 0.36723 |
| 292 | 0.37429 |
| 293 | 0.38194 |
| 294 | 0.39038 |
| 295 | 0.39966 |
| 296 | 0.40937 |
| 297 | 0.41947 |
| 298 | 0.43018 |
| 299 | 0.44143 |
| 300 | 0.45277 |
| 301 | 0.46428 |
| 302 | 0.47568 |
| 303 | 0.48726 |
| 304 | 0.49855 |
| 305 | 0.50983 |
| 306 | 0.52087 |
| 307 | 0.53184 |
| 308 | 0.5424 |
| 309 | 0.55261 |
| 310 | 0.56262 |
| 311 | 0.57229 |
| 312 | 0.58176 |
| 313 | 0.59074 |
| 314 | 0.59964 |
| 315 | 0.60836 |
| 316 | 0.61692 |
| 317 | 0.62487 |
| 318 | 0.63237 |
| 319 | 0.63904 |
| 320 | 0.64486 |
| 321 | 0.6497 |
| 322 | 0.65334 |
| 323 | 0.65561 |
| 324 | 0.65647 |
| 325 | 0.65614 |
| 326 | 0.65441 |
| 327 | 0.65124 |
| 328 | 0.6466 |
| 329 | 0.64033 |
| 330 | 0.63288 |
| 331 | 0.62406 |
| 332 | 0.6141 |
| 333 | 0.60272 |
| 334 | 0.59015 |
| 335 | 0.57627 |
| 336 | 0.56127 |
| 337 | 0.54538 |
| 338 | 0.5286 |
| 339 | 0.51124 |
| 340 | 0.49329 |
| 341 | 0.47516 |
| 342 | 0.45627 |
| 343 | 0.43716 |
| 344 | 0.41773 |
| 345 | 0.39755 |
| 346 | 0.37722 |
| 347 | 0.35704 |
| 348 | 0.33707 |
| 349 | 0.31722 |
| 350 | 0.29836 |
| 351 | 0.28015 |
| 352 | 0.26223 |
| 353 | 0.24491 |
| 354 | 0.22829 |
| 355 | 0.21213 |
| 356 | 0.19689 |
| 357 | 0.18233 |
| 358 | 0.16857 |
| 359 | 0.1549 |
| 360 | 0.14213 |
| 361 | 0.1304 |
| 362 | 0.11965 |
| 363 | 0.11018 |
| 364 | 0.10204 |
| 365 | 0.0946 |
| 366 | 0.08795 |
| 367 | 0.08211 |
| 368 | 0.07692 |
| 369 | 0.07234 |
| 370 | 0.06859 |
| 371 | 0.06518 |
| 372 | 0.06222 |
| 373 | 0.05977 |
| 374 | 0.05755 |
| 375 | 0.05567 |
| 376 | 0.05419 |
| 377 | 0.05288 |
| 378 | 0.05173 |
| 379 | 0.05076 |
| 380 | 0.05 |
| 381 | 0.04933 |
| 382 | 0.04887 |
| 383 | 0.04841 |
| 384 | 0.04801 |
| 385 | 0.04771 |
| 386 | 0.04735 |
| 387 | 0.04717 |
| 388 | 0.04696 |
| 389 | 0.04674 |
| 390 | 0.04651 |
| 391 | 0.04646 |
| 392 | 0.04636 |
| 393 | 0.04626 |
| 394 | 0.04622 |
| 395 | 0.04621 |
| 396 | 0.04612 |
| 397 | 0.04609 |
| 398 | 0.04608 |
| 399 | 0.0461 |
| 400 | 0.0461 |
| 401 | 0.04602 |
| 402 | 0.04595 |
| 403 | 0.04582 |
| 404 | 0.04579 |
| 405 | 0.04565 |
| 406 | 0.04578 |
| 407 | 0.0457 |
| 408 | 0.04576 |
| 409 | 0.0457 |
| 410 | 0.04575 |
| 411 | 0.0457 |
| 412 | 0.04572 |
| 413 | 0.04572 |
| 414 | 0.04566 |
| 415 | 0.04574 |
| 416 | 0.04575 |
| 417 | 0.04583 |
| 418 | 0.04573 |
| 419 | 0.04574 |
| 420 | 0.04564 |
| 421 | 0.04561 |
| 422 | 0.04565 |
| 423 | 0.04564 |
| 424 | 0.04569 |
| 425 | 0.04571 |
| 426 | 0.04571 |
| 427 | 0.04565 |
| 428 | 0.04564 |
| 429 | 0.04555 |
| 430 | 0.04541 |
| 431 | 0.04543 |
| 432 | 0.04547 |
| 433 | 0.04544 |
| 434 | 0.04556 |
| 435 | 0.04557 |
| 436 | 0.04554 |
| 437 | 0.04548 |
| 438 | 0.04551 |
| 439 | 0.0455 |
| 440 | 0.04547 |
| 441 | 0.04561 |
| 442 | 0.04554 |
| 443 | 0.04555 |
| 444 | 0.04556 |
| 445 | 0.04539 |
| 446 | 0.04531 |
| 447 | 0.04521 |
| 448 | 0.04519 |
| 449 | 0.04525 |

**Figure S25** UV-Vis spectra of **NBD1** in propylene carbonate solution at 4.92 x 10^-5^ M (purple), neat solutions of propylene carbonate (blue), sulfolane (green), and Cyrene (red).

The UV–vis cut‑off of a solvent is defined as the wavelength at which its absorbance reaches approximately 1. Under our measurement conditions, sulfolane exhibits a cut‑off at 384 nm, Cyrene at 386 nm, and propylene carbonate below 320 nm.

# **3 Propylene Carbonate**

## Photostability

Two NMR samples were prepared, one containing propylene carbonate and the other containing Cyrene, both dissolved in deuterated toluene, which was selected for its excellent photostability. 1,4-Dioxane was added to each sample as an internal standard to verify that the total amount of material remained consistent between samples.

**Figure S26** Propylene carbonate with a 1,4-dioxane internal standard in deuterated toluene.

**Figure S27** Long term 24-hour UVA excitation of propylene carbonate with a 1,4-dioxane internal standard in deuterated toluene.

**Figure S28** Cyrene with a 1,4-dioxane internal standard in deuterated toluene.

**Figure S29** Long term 24-hour UVA excitation of Cyrene with a 1,4-dioxane internal standard in deuterated toluene.

Photos were taken before and after photoisomerization; for propylene carbonate, a colour change is observed to a lighter yellow, whilst for Cyrene, the colour remains similar. Initial Propylene carbonate is colourless and Cyrene is yellow.

**A**

**B**

**Figure S30** **A**) NBD in propylene carbonate (left) and NBD in Cyrene (right). **B**) UVA excitation of NBD in propylene carbonate (left) and UVA excitation of NBD in Cyrene (right).

## Thermal stability

Two NMR samples were prepared, one containing propylene carbonate and the other containing Cyrene, both dissolved in deuterated toluene, which was selected for its excellent thermal stability. 1,4-Dioxane was added to each sample as an internal standard to verify that the total amount of material remained consistent between samples.

**Figure S31** Propylene carbonate with a 1,4-dioxane internal standard in deuterated toluene.

**Figure S32** Heating for 24 hours at 80 ˚C of propylene carbonate with a 1,4-dioxane internal standard in deuterated toluene.

**Figure S33** Cyrene with a 1,4-dioxane internal standard in deuterated toluene.

**Figure S34** Long term 24-hour UVA excitation of Cyrene with a 1,4-dioxane internal standard in deuterated toluene.

**Figure S35** Cyrene with a 1,4-dioxane internal standard in deuterated toluene.

**Figure S36** Heating for 24 hours at 80 ˚C of propylene carbonate with a 1,4-dioxane internal standard in deuterated toluene.

## Calibration plots of NBD1 in propylene carbonate and toluene

A concentration versus absorbance graph was plotted in which the molar extinction coefficients of **NBD1** in both media are represented by the gradient of the plot over the concentration ranges.

Absorbance (a.u)

| Concentration (M) | NBD1 in PC |
| --- | --- |
| 1.49333e-05 | 0.1646 |
| 2.24e-05 | 0.2189 |
| 2.98667e-05 | 0.3234 |
| 4.48e-05 | 0.5153 |
| 6.72e-05 | 0.687 |

**Figure S37** Calibration curve of **NBD1** in propylene carbonate, absorbance value taken at 340 nm.

The molar extinction coefficient of **NBD1** in propylene carbonate at 340 nm is 10600 M^-1^ cm^‑1^.

**Figure S38** Calibration curve of **NBD1** in toluene, absorbance value taken at 340 nm.

| Concentration (M) | Absorbance (a.u.) |
| --- | --- |
| 4.47888e-05 | 0.37638 |
| 6.27043e-05 | 0.54257 |
| 8.95776e-05 | 0.82513 |
| 0.000125409 | 1.14867 |

A calibration curve of the **NBD1** compound in the concentration range used to measure the progress of both photoisomerisation and catalytic reactions was done using UV-Vis spectrometry. The molar extinction coefficient of **NBD1** in toluene at 340 nm is 9060 M^-1^ cm^-1^.

## Potassium ferrioxalate actinometry

Before the quantum yield of photoisomerisation measurements were performed, the photon flux of the irradiation source (Hg lamp 313 nm) was determined by potassium ferrioxalate actinometry.^[10]^

Absorbance (a.u)

| Concentration (M) | Fe2+ calibration curve |
| --- | --- |
| 0 | -0.0006 |
| 1 | 0.1878 |
| 3 | 0.4177 |
| 5 | 0.8898 |
| 7 | 1.2363 |
| 7 | 1.229 |

**Figure S39** Calibration curve for Fe^2+^ species.

### Preparation of the actinometry solution

Two solutions were prepared, first a standard 25 mL solution of K_2_C_2_O_4_ of 1.2 M concentration, and second a 25 mL solution of Fe_2_(SO_4_)_3_ of 0.2 M concentration. The actinometer solution is produced by pipetting 5 mL of Fe_2_(SO_4_)_3_ solution and 5 mL of K_2_C_2_O_4_ solution into a 100 mL volumetric flask and diluting to the mark with demineralised water. The actinometer solution is kept in the dark until required and has a shelf life of approximately one month, although for greater precision in the measurements, solutions were prepared fresh.

### Photon flux measurement

A 0.2% by weight 25 mL solution of 1,10-phenanthroline in water was prepared. A 100 mL buffer solution was prepared in a volumetric flask by adding 8.2 g of anhydrous sodium acetate, 1 mL of sulfuric acid, and diluted to the mark with demineralised water. A blank solution (for the calibration curve) was prepared with 1 mL of buffer solution and 1 mL of 1,10-phenanthroline in a 3 mL cuvette, and the absorption spectrum was measured. A 3 mL sample of the actinometer solution was irradiated for 15 seconds, and 1 mL of the irradiated actinometer solution (V_1_) diluted in a 10 mL volumetric flask with 2 mL of phenanthroline solution (V_3_) and 0.5 mL of buffer solution (V_2_), diluted to the mark with demineralised water under dark conditions. 3 mL of this solution was placed in a 1 cm pathlength (l) quartz cuvette and the absorption spectrum measured. This procedure was repeated for four more samples, with varying irradiation times of the actinometer solution, from 15 seconds to 75 seconds, with intervals of 15 seconds.

The photon flux *I* (E.s^-1^) can then be calculated from the equation below:

$I=gradient*\frac{V2.V3}{\varepsilon(510 nm).d.\phi.V1}$	Equation 2

Where *ε_510nm_* corresponds to the absorbance of tri-phenanthroline complex at 510 nm, which is equal to 1.11 x 10^4^ M^-1^ cm^-1^. And 𝜙 is the photochemical quantum yields (1.24) of ferrioxalate decomposition formed from 313 nm irradiation.^15^ Where *V_1_* is the volume (in millilitres) of the irradiated actinometer solution taken from the cuvette. Where *V_2_* is the volume of actinometer solution irradiated (in litres), and *V_3_* is the volume of total solution to dilute the irradiated actinometer solution (in millilitres).

Absorbance (a.u)

| Time (seconds) | ferrous phenanthroline complex production |
| --- | --- |
| 0 | 0.0018 |
| 15 | 0.1264 |
| 30 | 0.2276 |
| 45 | 0.3118 |
| 60 | 0.4149 |
| 75 | 0.5092 |

**Figure S40** Calibration curve for irradiated actinometry solution at specific time intervals.

The photon flux of the 313 nm Hg light source was calculated as 9.62 x 10^15^ s^-1^.

## Photoisomerisation Quantum Yield

**Experimental setup**

The photoisomerisation quantum yield measurement is based on the reported method using a high concentration regime.^15^ An expression for the quantum yield from a photoisomerisation event is given below; this factor determines the efficiency of the photoisomerisation reaction.

$\phi iso= \frac{\mathrm{number} \mathrm{of} \mathrm{isomerisation} \mathrm{events}}{\mathrm{number} \mathrm{of} \mathrm{photons} \mathrm{absorbed}}$	Equation 3

The photoisomerisation quantum yield was recorded following the full absorption regime. To ensure that all photons were absorbed, the concentration of the solutions was prepared to be optically thick at the irradiation wavelength; two solutions of NBD were prepared in which the absorbance at 340 nm is greater than 2. The sample of NBD was irradiated until the photoisomerisation was complete (the absorbance no longer changed), and the absorbance changes were recorded at specific time intervals. When all photons were absorbed, a linear dependence between the decrease in absorption of NBD and the irradiation time can be obtained from the equation below.

[𝑁𝐵𝐷] = [𝑁𝐵𝐷_𝑖𝑛𝑖𝑡𝑖𝑎𝑙_] ‒ $\frac{\phi\mathrm{iso} \cdot I}{N_{A} \cdot V}$∙ 𝑡_𝑖𝑟𝑟_	Equation 4

Where *[NBD]* and *[NBD_initial_]* correspond to the actual and initial concentration of NBD in solution, respectively. *N_A_* is the Avogadro constant; *V* is the volume of the irradiated sample; I is the photon flux, and *t_irr_* is the irradiation time. Another comparative measurement was performed; the reported quantum yield is an average of two measurements.

To perform the photoisomerisation of NBD to QC reproducibly, an in-house setup comprised of an Oriel Cornerstone 130 1/8 m monochromator coupled with a Quantum Northwest qpod-2e thermostatic sample holder was used. A 500 W Hg arc lamp from Oriel (USA) with an Oriel water filter was used as a light source. The sample holder is a Peltier temperature-controlled system. The sample holder is attached to an Ocean Optics USB4000 UV-Vis spectrometer to allow UV-Vis measurements to be recorded simultaneously. The sample holder is made for standard 1 cm quartz cuvettes.

**Figure S41** Oriel Cornerstone 130 monochromator attached to a qpod-2e holder, which is subsequently attached to a UV-Vis spectrometer from Ocean Optics.

A 313 nm light source was used to irradiate the samples. Each sample was placed in a thermally regulated box in which the temperature was set to 25 °C. A 7 x 2 mm stirrer bar was placed in the solution, and a stirring rate of 1200 rpm was applied to ensure a homogeneously distributed solution. At higher concentrations the solution becomes optically dense, leading to a pronounced inner filter effect limiting light penetration and reducing photoisomerisation efficiency.^15^

**NBD1** was weighed using an analytical balance, where the uncertainty in the mass is ± 0.1 mg. Two 10 mL vials of **NBD1** were prepared, one in toluene and one in acetonitrile media. 1.54 mg was dissolved in 10 mL of propylene carbonate, resulting in a concentration of 6.9 x 10^-4^ M. The concentration was calculated using the Beer–Lambert law. Whilst 1.18 mg was dissolved in 10 mL of toluene, resulting in a concentration of 5.3 x 10^-4^ M. The molar absorption coefficient of QC was obtained by fully photo-converting a known concentration of **NBD1** and recording the spectrum until no further changes occurred in the absorption profile. Molar absorption coefficients of QC and NBD at 340 nm are 170 M^-1^ cm^-1^ and 10600 M^-1^ cm^-1^ in Propylene carbonate, respectively. Molar absorption coefficients of QC and NBD at 340 nm are 130 M^-1^ cm^-1^ and 9060 M^-1^ cm^-1^ in toluene, respectively. The two reported quantum yield values correspond to two independent measurements, for which separate samples were prepared and analysed. The mean and standard deviation of the quantum yield can be calculated as 88.5 ± 0.7% in propylene carbonate.

| Time (seconds) | [NBD] |
| --- | --- |
| 0 | 0.00069 |
| 7 | 0.000640159 |
| 14 | 0.00059668 |
| 21 | 0.000556383 |
| 28 | 0.000518207 |
| 35 | 0.000482859 |
| 42 | 0.000453519 |
| 49 | 0.000421176 |

**Figure S42** NBD to QC conversion in propylene carbonate using a Hg light source at a wavelength of 313 nm. The QY of photoisomerisation is 89%.

| Time (seconds) | [NBD] |
| --- | --- |
| 0 | 0.0006906 |
| 7 | 0.000641217 |
| 14 | 0.000596427 |
| 21 | 0.000556806 |
| 28 | 0.000517759 |
| 35 | 0.000487325 |
| 42 | 0.000456125 |
| 49 | 0.00042684 |

**Figure S43** NBD to QC conversion in propylene carbonate using a Hg light source at a wavelength of 313 nm. The QY of photoisomerisation is 88%.

| Time (seconds) | [NBD] |
| --- | --- |
| 0 | 0.00053 |
| 7 | 0.000494229 |
| 14 | 0.000464506 |
| 21 | 0.000435647 |
| 28 | 0.000406961 |
| 35 | 0.000383978 |
| 42 | 0.000356847 |
| 49 | 0.000333345 |

**Figure S44** NBD to QC conversion in toluene using a Hg light source at a wavelength of 313 nm. The QY of photoisomerisation is 64%.

| Time (seconds) | [NBD] |
| --- | --- |
| 0 | 0.000587288 |
| 7 | 0.000558433 |
| 14 | 0.000531147 |
| 21 | 0.000503703 |
| 28 | 0.000479867 |
| 35 | 0.000454462 |
| 42 | 0.000431723 |
| 49 | 0.000409298 |

**Figure S45** NBD to QC conversion in acetonitrile using a Hg light source at a wavelength of 313 nm. The QY of photoisomerisation is 59%.

As the photoisomerization quantum yield could not be measured using the UV-Vis spectrometry (through actinometry) for Cyrene solution as the solvent absorbs in the same region as norbornadiene, saturating the detector, comparison reactions were performed in both Cyrene and propylene carbonate. Sufficiently high concentrations of sample were prepared to determine which reaction undergoes a more efficient photoisomerization. Two solutions of 12.8 mg in 750 microlitre and 10.1 mg in 750 microlitre total (Cyrene and propylene carbonate) were prepared in d^8^ toluene (500 microlitre) with an internal standard of 1,4-dioxane (50 microlitre). The NMR tubes were photoisomerised using a 16 W 18 lamp UVA light source for 2 hours and measured. The conversions of the reaction were calculated directly from the integrals at 7.7 ppm belonging to the NBD species and 7.15 ppm belonging to the QC species. As both these environments correspond to 2 equivalent protons the following simplified equation was used:

%NBD = $\frac{I\mathrm{NBD}}{I\mathrm{QC} + I\mathrm{NBD}}$ x 100

Where I_NBD_ is the integral for NBD species and I_QC_ is the integral for the QC species.

In Propylene carbonate: 2 / 2 + 4.93 = 28.9 %

In Cyrene: 2 / 2 + 18.02 = 10.0 %

The photoisomerization reactions approach near full conversion in Cyrene at 90 % converted and approximately 71% converted in propylene carbonate suggesting that Cyrene solvent has an improved photoisomerization quantum yield to propylene carbonate. The NMR spectra of the photoisomerization reactions are shown below in Figure S38-S41.

**Figure S46** NBD in Propylene carbonate.

**Figure S47** Two hours of UVA excitation of NBD in Propylene carbonate.

**Figure S48** NBD in Cyrene.

**Figure S49** Two hours of UVA excitation of NBD in Cyrene.

From these results, an explanation can be proposed. The norbornadiene derivative possesses a push–pull electronic structure, giving rise to a polar ground state and promoting intramolecular charge transfer upon photoexcitation. The observed solvent dependence suggests that multiple solvent properties influence the photoisomerization behaviour of the NBD derivative. Although both acetonitrile and propylene carbonate possess high dielectric constants, they produce markedly different photoisomerization quantum yields, indicating that dielectric constant alone cannot account for the observed trends. Under identical reaction conditions, photoisomerization was more efficient in Cyrene than in propylene carbonate. Since Cyrene and acetonitrile have similar dielectric constants but substantially different viscosities, these results suggest that viscosity plays a more significant role in determining photoisomerization efficiency than dielectric constant. Furthermore, despite the significant spectral overlap between the absorption band of the photoswitch and Cyrene, which would be expected to reduce the effective photon flux through inner-filter effects, Cyrene still outperformed propylene carbonate. This indicates that the beneficial effect of viscosity outweighs any reduction in light absorption caused by spectral overlap.

A possible explanation is that low-viscosity solvents reorganize more rapidly around the photoexcited charge-transfer state, facilitating non-productive relaxation pathways that compete with photoisomerization. In contrast, the higher viscosity of Cyrene and propylene carbonate solvents may restrict both solvent and solute motions, slowing solvent relaxation around the excited state, allowing it to persist longer, favouring productive isomerisation.

## Kinetics of the thermal QC1 to NBD1 back-reaction

A toluene solution of 4.48 x 10^-5^ **NBD1** was irradiated to obtain **QC1** until no further change was observed in the absorbance profile. Thereafter, the increase in **NBD1** concentration over time was measured by monitoring the increase in absorption at 340 nm. The measurements were performed at five different temperatures ranging from 40 to 80 °C, and an exponential fit of the Eyring equation was applied to the data to determine the rate constants at these temperatures.

ln ([QC])

| Time (seconds) | 80 C |
| --- | --- |
| 0 | -10.306 |
| 150 | -10.343 |
| 300 | -10.3862 |
| 450 | -10.4324 |
| 600 | -10.4784 |

**Figure S50** QC to NBD thermal back-reaction at 80° C in propylene carbonate monitored every five minutes.

ln ([QC])

| Time (seconds) | 70 C |
| --- | --- |
| 0 | -10.215 |
| 600 | -10.2475 |
| 1200 | -10.2841 |
| 1800 | -10.317 |
| 2400 | -10.3528 |
| 3000 | -10.3923 |

**Figure S51** QC to NBD thermal back-reaction at 70° C in propylene carbonate monitored every five minutes.

ln ([QC])

| Time (seconds) | 60 C |
| --- | --- |
| 0 | -10.2339 |
| 600 | -10.2472 |
| 1200 | -10.2571 |
| 1800 | -10.2679 |
| 2400 | -10.282 |
| 3000 | -10.2906 |
| 3600 | -10.3018 |
| 4200 | -10.3131 |

**Figure S52** QC to NBD thermal back-reaction at 60° C in propylene carbonate monitored every five minutes.

ln([QC])

| Time (seconds) | 50 C |
| --- | --- |
| 0 | -10.1419 |
| 2400 | -10.1513 |
| 4800 | -10.1641 |
| 7200 | -10.1785 |
| 9600 | -10.1937 |
| 12000 | -10.2076 |

**Figure S53** QC to NBD thermal back-reaction at 50° C in propylene carbonate monitored every five minutes.

ln ([QC])

| Time (seconds) | 40 C |
| --- | --- |
| 0 | -10.2276 |
| 3000 | -10.2292 |
| 6000 | -10.2375 |
| 9000 | -10.242 |
| 12000 | -10.2507 |
| 15000 | -10.256 |
| 18000 | -10.2581 |
| 21000 | -10.2629 |
| 24000 | -10.2754 |
| 27000 | -10.2797 |
| 30000 | -10.2885 |
| 33000 | -10.291 |
| 36000 | -10.2982 |
| 39000 | -10.3035 |
| 42000 | -10.3077 |
| 45000 | -10.3153 |
| 48000 | -10.3162 |
| 51000 | -10.3236 |
| 54000 | -10.3278 |
| 57000 | -10.3347 |
| 60000 | -10.3397 |
| 63000 | -10.3466 |
| 66000 | -10.3507 |
| 69000 | -10.3572 |
| 72000 | -10.3613 |
| 75000 | -10.3649 |

**Figure S54** QC to NBD thermal back-reaction at 40° C in propylene carbonate monitored every five minutes.

ln(k/T)

| 1/T (1/K) | Propylene carbonate |
| --- | --- |
| 0.00319489 | -18.9304 |
| 0.00309598 | -17.8722 |
| 0.003003 | -16.6951 |
| 0.00291545 | -15.5791 |
| 0.00283286 | -14.0156 |

**Figure S55** Eyring plot from kinetic profiles of **NBD1** in propylene carbonate in the temperature range of 40 – 80°C.

**Table S9** Experimental thermodynamic data of the QC to NBD back reaction in propylene carbonate.

| **Temperature (K)** | 313 | 323 | 333 | 343 | 353 |
| --- | --- | --- | --- | --- | --- |
| **1/Temperature (K)** | 0.00320 | 0.00310 | 0.00300 | 0.00292 | 0.00283 |
| **Gradient (*k*)** | 1.88 x 10^-6^ | 5.59 x 10^-6^ | 1.87 x 10^-5^ | 5.88 x 10^-5^ | 2.89 x 10^-4^ |
| **1/T x *k*** | 6.00 x 10^-9^ | 1.73 x 10^-8^ | 5.62 x 10^-8^ | 1.71 x10^-7^ | 8.19 x 10^-7^ |
| **Ln (*k*/T)** | -18.93 | -17.87 | -16.70 | -15.58 | -14.01 |

**ΔH^‡^** = 111.03 kJ/mol

**ΔS^±^** = -1.70 J/mol.K

The enthalpy (ΔH^ǂ^_thermal_) and entropy (ΔS^ǂ^_thermal_) of activation were derived from the logarithmic form of the Eyring equation presented, and the **half-life** at 25 °C was calculated to be **44.7 days** in propylene carbonate.
Catalytic back reactions from QC1 to NBD1 in propylene carbonate and toluene

Catalytic reactions were carried out according to the following procedure. A 10 mL solution of **NBD1** in the respective solvent was prepared at a concentration of 4.48 × 10⁻³ M (1 mg mL^‑1^). The solution was irradiated under UVA light (315–400 nm, λ_max_ = 326 nm) for 90 minutes. Catalysis was initiated once the residual **NBD1** content had decreased to below 2%. Screening experiments employed a catalyst loading of 10 wt% (1 mg) or 10 mol% relative to starting mass of NBD reactant used.

**Table** **S10** Relative weight loadings and corresponding mol % of metal or metal complex used in the catalytic conversion of QC1 to NBD1. *Note the metal loadings for Pt/C and Cu/C were obtained from previous measurements.^16^ An approximate 20 wt% loading of CoPc was used in the synthesis of CoPc/C. A 35.1 wt% loading of the commercial Au/C was obtained from thermogravimetric analysis performed by Nanoscientifica^®^.

| **Catalyst** | **Metal loading (wt%)** | **Moles of metal/metal complex** | **Mol% vs NBD** |
| --- | --- | --- | --- |
| Pt/C | 8.1 | 4.15 x 10^-7^ | 0.93 |
| Cu/C | 5.9 | 9.28 x 10^-7^ | 2.07 |
| CoPc/C | ~20 | 3.48 x 10^-7^ | 0.78 |
| Au/C | 35.1 | 1.78 x 10^-6^ | 3.97 |
| CoPc | - | 4.48 x 10^-6^ | 10 |

Reaction progress was monitored at defined time intervals with stirring maintained at 500 rpm. Prior to UV–Vis analysis, samples were diluted 1:100 in the respective solvent and filtered through 0.22 μm membranes to remove catalyst particles. Quantification of the isomerisation was performed using a Shimadzu UV–Vis spectrometer, with **NBD1** conversion % recorded at 340 nm. This wavelength was selected to avoid overlap with **QC1** absorption and to ensure linear response across the relevant concentration range.

**Figure S56** QC to NBD catalytic back-reactions in 1 mg/mL solutions of toluene and Propylene carbonate using 10 weight % of Pt/C catalyst.

**Figure S57** QC to NBD catalytic back-reactions in 1 mg/mL solutions of toluene and Propylene carbonate using 10 weight % of CoPc catalyst.

**Figure S58** QC to NBD catalytic back-reactions in 1 mg/mL solutions of toluene and Propylene carbonate using 10 weight % of CoPc carbonate/C catalyst.

**Figure S59** QC to NBD catalytic back-reactions in 1 mg/mL solutions of toluene and Propylene carbonate using 10 weight % of *Au*/C catalyst.

**Figure S60** QC to NBD catalytic back-reactions in 1 mg/mL solutions of toluene and Propylene carbonate using 10 weight % of Cu/C catalyst.

ln[QC]

| Time (minutes) | Cu/C | CoPc | CoPc/C | Pt/C | Au/C |
| --- | --- | --- | --- | --- | --- |
| 0 | -9.97983 | -10.2552 | -10.4289 | -10.3644 | -10.2015 |
| 2 | -10.0185 | -10.2996 | -10.5901 | -10.5944 | -10.4831 |
| 5 | -10.0789 | -10.349 | -10.6627 | -10.6376 | -10.8009 |
| 10 | -10.1621 | -10.3959 | -10.7361 | -10.6737 | -11.5464 |
| 30 | -10.3827 | -10.6378 | -11.0007 | -10.9254 | -12.9267 |

**Figure S61** Reaction rates of five catalysts for the QC to NBD back-reaction in propylene carbonate applying the first order kinetics in the logarithmic form.

**Figure S62** Reaction rates of five catalysts for the QC to NBD back-reaction in toluene applying the first order kinetics in the logarithmic form.

ln[QC]

| Time (minutes) | Cu/C | CoPc | CoPc/C | Pt/C | Au/C |
| --- | --- | --- | --- | --- | --- |
| 0 | -10.203 | -10.014 | -10.23 | -10.2175 | -10.0038 |
| 2 | -10.234 | -10.0959 | -10.65 | -10.6555 | -10.6425 |
| 5 | -10.3495 | -10.1425 | -10.7613 | -10.704 | -11.2 |
| 10 | -10.5943 | -10.3854 | -11.0467 | -11.2214 | -11.7547 |
| 30 | -11.3271 | -10.9156 | -11.6302 | -11.9266 | -13.4742 |

**Table S11** Reaction rates of the catalytic reactions conducted in propylene carbonate as calculated from the first order rate constant.

| **Solvent** | **Rate constant (k, s^-1^) in Propylene carbonate** | **Rate constant (k, s^-1^) in toluene** | **Rate ratio (k_toluene_/k_Propylene carbonate_)** |
| --- | --- | --- | --- |
| Pt/C | 1.51 x 10^-2^ | 5.18 x 10^-2^ | 3.4 |
| Cu/C | 1.32 x 10^-2^ | 3.83 x 10^-2^ | 2.9 |
| Au/C | 8.89 x 10^-2^ | 1.07 x 10^-1^ | 1.2 |
| CoPc/C | 1.66 x 10^-2^ | 4.07 x 10^-2^ | 2.5 |
| CoPc | 1.23 x 10^-2^ | 3.57 x 10^-2^ | 2.9 |

**Figure S63** Catalytic reactions of **NBD1** in propylene carbonate using Pt/C catalyst.

**Table S12** Data points of experiments, mean and standard deviation between experiments.

|  | **Experiment 1** | **Experiment 2** | **Mean** | **Standard Deviation** |
| --- | --- | --- | --- | --- |
| 0 | 0.0363 | 0.03846 | 0.03738 | 0.00153 |
| 2 | 0.0857 | 0.10109 | 0.0934 | 0.01088 |
| 5 | 0.09707 | 0.11881 | 0.10794 | 0.01537 |
| 10 | 0.12365 | 0.13563 | 0.12964 | 0.00847 |
| 30 | 0.16163 | 0.1824 | 0.17202 | 0.01469 |
| 60 | 0.19215 | 0.21408 | 0.20312 | 0.01551 |

## Solubility of NBD in Propylene Carbonate

In a vial, **NDB1** was suspended in 2 mL of propylene carbonate, and more solid was added until no solid remained. Then the suspensions were sonicated for 20 minutes. Afterwards, the suspension was filtered twice through a syringe filter (0.22 μm) to ensure no solid remained in the solution attaining a saturated solution. 4 μL of the saturated solution was diluted with 10 mL of propylene carbonate and then diluted again by taking 30 uL and diluting it in 2 mL of propylene carbonate. The UV-Vis spectrum was acquired of the following solution. The last dilution was done in triplicate to guarantee the reproducibility of the measurements.

The concentration was determined using the molar extinction coefficient of **NBD1** in propylene carbonate at 340 nm, which is 10600 M^-1^ cm^‑1^, as determined previously. A cuvette path length of 1 cm was used.

**Table S13** Solubilities of **NBD1** in propylene carbonate.

| **Saturated Sample Number** | **Repetition** | **Abs 340 nm of**  Rediluted solution | **Saturated [M]** | **Average [M]** | **Error** |
| --- | --- | --- | --- | --- | --- |
| 1 | 1  2  3 | 0.13717  0.17954  0.16273 | 2.18999  2.86645  2.59807 | 2.995 | 0.421 |
| 2 | 1  2  3 | 0.19975  0.1823  0.17551 | 3.18911  2.91051  2.80211 |  |  |
| 3 | 1  2  3 | 0.21922  0.22328  0.20874 | 3.49996  3.56478  3.33264 |  |  |

As the molar extinction coefficient of **NBD1** in cyrene could not be determined due to the significant spectral overlap with the solvent, the measurements were performed by dissolving 50 mg of NBD in the minimum amount of cyrene possible. Five experiments were performed, and the average solubility was calculated. Errors transpire from the precision of the mass balance and micropipette used.

**Table S14.** Solubilities of NBD in Cyrene.

| **Run** | **Solubility (mg mL^-1^)** | **Solubility (mol dm^-3^)** |
| --- | --- | --- |
| 1 | 218 | 0.978 |
| 2 | 196 | 0.879 |
| 3 | 222 | 0.996 |
| 4 | 202 | 0.906 |
| 5 | 198 | 0.888 |

The average of the five tests is calculated at 0.93 M. Standard deviation across the samples is 0.0538 M.

**A**

**B**

**Figure S64** **A**) 0.93 M solution of NBD in Cyrene and **B**) 2.47 M solution of NBD in propylene carbonate measured at 22 ˚C.

## Photoconversion of NBDs under flow conditions

In order to convert the selected compound **NBD1** to its **QC1** isomer ready for the heat release experiment, photoconversions under flow conditions were carried out using Vapourtec R-series flow systems equipped with a photo-irradiation unit, a Vapourtec UV-150 photochemical reactor. For the photoconversion, a 365 nm LED was chosen (Vapourtec 365 nm Gen-2, 16 W radiant power) for the experiment, and its power was set to 100%. The volume of the sample loop for the photoirradiation was 10 mL.

**Figure S65.** General scheme for photoconversion of NBD to QC under flow conditions in this study.

A NBD solution in propylene carbonate (1.8 M, 10 mL) was photoirradiated using the Vapourtec flow system under 365 nm LED irradiation at a flow rate of 0.333 mL min^-1^.

**Figure S66.** ^1^H NMR spectra of **NBD1**/**QC1** sample in CDCl_3_ after the photoirradiation in flow in Pc. Note that there is water and Pc present.

The conversion yield was determined by ^1^H NMR spectroscopy in CDCl_3_, and the composition of the sample was the following: NBD 30.6%, QC 69.4%. As the sample was not successfully fully converted, to fully test the macroscopic potential, the sample was irradiated in toluene and then redissolved in propylene carbonate.

The sample for the macroscopic heat release was therefore prepared using the following procedure:

The solution of **NBD1** in toluene (1.0 g, 4.48 mmol 1.5 M, 3.0 mL) was pumped through the photoreactor with a flow rate of 0.333 mL min^-1^ (Residence time of t_res_ = 30 min). The solvent was evaporated after the reaction with a Biotage V-10 Touch evaporation system applying the following conditions three times: Target Temperature for irradiation 30 °C, Target Pressure 8 mbar, Vial size: 20 mL. ^1^H NMR spectrum was recorded after the experiment to determine the conversion percentage. After the photoconversion experiment in flow, the sample was 100% converted to the **QC1**. The **QC1** was stored at -28 °C until used for the heat release experiment.

**Figure S67.** ^1^H NMR of **NBD1** in CDCl_3_ after flow irradiation at 365 nm in toluene (toluene was removed as described). Note that there is water from the solvent.

The solution was prepared by dissolving **QC1** (0.57 g, 2.55 mmol) in 1.41 mL of Propylene carbonate using an ultrasonication bath. The solution was used immediately without any further treatment. ^1^H NMR spectra were recorded before and after the experiment to determine the conversion percentage.

## Heat release experiment using Pt/C catalytic bed in Propylene carbonate

The setup for the macroscopic heat release is depicted schematically in **Figure S52**, along with a photo. A similar setup has been published previously. In the central part of the setup, inside the vacuum chamber, is a 3.0 cm Teflon tubing (1.27 mm inner diameter) with a loading length of 1.5 cm, with cotton to block each side, from which 1 cm corresponds to the loaded catalyst Pt/C (5.4 mg). The measured temperature increase corresponds to the outlet temperature of the reaction stream after the catalyst bed and is used as a measure of the thermal response. The experiments were performed under high vacuum (~10⁻⁵ mbar), thereby effectively eliminating convective heat transfer. Furthermore, the short fixed-bed reactor (1 cm) and PTFE tubing are expected to minimize conductive heat losses, while the short residence time is expected to limit radiative losses. Consequently, the measured temperature increase is expected to closely reflect the thermal response of the reaction stream, although the setup was not designed as a calibrated calorimeter and no quantitative reaction enthalpies are derived from these measurements.

**Figure S68.** Schematic of the vacuum setup (left) and photo of the actual setup (right).

As reference experiment, a solution of only propylene carbonate was passed through the fixed bed catalytic reactor. The temperature change was measured using the home-built vacuum heat-release system. The solution was passed through the catalyst at a flow rate of 5 mL h^-1^. The system operated at a vacuum pressure of 7.1 × 10^-3^ mbar.

**Figure S69.** Temperature measurement passing Pc through the catalyst.

The 1.8 M solution in Propylene carbonate (1.41 mL) was pumped through the setup with a flow rate of 5 mL h^-1^ (83 μL min^-1^). The vacuum varied in the range of 10^-4^-10^-5^ mbar during the experiment. We observed a heat release with a temperature increase of 50.75 ⁰C. Due to pressure within the system, we observed a drop after the first rise; however, continuing the experiment, we observed a second temperature increase up to 62.93 °C. We did not use the total volume of 1.9 mL for the experiment. The initial working temperature was 23.62 ⁰C before the solution hit the catalyst. ^1^H NMR spectra were recorded before and after the experiment to determine the conversion percentage (**Figure S54**). After the heat release experiment, the composition of the sample was the following: NBD 71.4%, QC 28.6%.

**Figure S70.** ^1^H NMR spectrum of a highly concentrated solution of Propylene carbonate and CDCl_3_.

**Figure S71.** ^1^H NMR spectra of **QC1** sample in Propylene carbonate and CDCl_3_ before (top) and after (bottom) the heat release experiment. Note that there is water (1.66 ppm) from the deuterated solvent.

## Macroscopic Heat Release

An estimation of the macroscopic temperature increase was calculated based on the experimental *ΔH*_storage_ value from previous experiments with a CoPc catalyst,^17^ and the following equation for *Δ*T:

$\Delta T = \frac{c\cdot M_{w}\cdot\Delta H_{storage}}{c\cdot M_{w}\cdot C_{p}(PS)+ \rho_{solvent}\cdot C_{p}(solvent)}$	Equation 5

where c and M_w_ represent the concentration of the reactive photoswitch species and molecular weight, respectively; *ΔH*_storage_ is the DSC-measured energy storage capacity of the photoswitch (PS) couple at 400 J g^-1^; *C_p_* (PS) is the specific heat capacity of the photoisomer at 1.66 J g^-1^ K^-1^; and *ρ*_solvent_ and *C_p_* (solvent) correspond to the volumetric mass density in g L^-1^ and the specific heat capacity in J g^-1^ K^-1^ of the solvent (here Propylene carbonate, 1200 g L^-1^ and 1.64 J g^-1^ K^-1^).^6^ The theoretical temperature release from a 3.0 M NBD-QC system was calculated at approximately 87.0 ⁰C. For the flow reaction, as the sample was not fully back-converted after the heat release experiment, based on the NMR after back conversion, the *ΔT* we observed is based only on the back conversion of 71.4% to the parent species that was present in the sample after the experiment. Thus, from the initial 1.8 M solution, only 1.29 M resulted in the heat release. We estimated a theoretical heat release to result in a ΔT of 47 ⁰C and 61 ⁰C for a 1.29 M and 1.8 M reactive photoisomer species, respectively, using the specific heat capacity *C_p_* (PS) based on previous experiments.^16^

## Cycling experiments

A 25 mL, 0.18 M solution of NBD (approximately 1.0 g) was prepared in propylene carbonate. The solution was irradiated using a 16 W UVA light source until less than 10% of the NBD remained in the mixture. The extent of NBD-to-QC conversion was determined by ^1^H NMR spectroscopy. The resulting QC solution was subsequently subjected to catalytic back-conversion using 1 weight % Pt/C catalyst (10 mg), relative to the initial mass of NBD (1g). Due to the gram-scale amount of photoswitch employed, a lower catalyst loading of 1 wt% Pt/C was used instead of the 10 wt% loading employed for the lower milligram-scale experiments. This choice was primarily dictated by the limited availability of the catalyst material and additionally facilitated its removal from the reaction mixture after completion of the catalytic reaction. Upon completion of the catalytic reaction, the Pt/C catalyst was separated from the reaction mixture by centrifugation. The centrifugation procedure was repeated three times. The supernatant was then filtered through a 0.22 µm PTFE filter to ensure efficient removal of the catalyst. The recovered NBD solution was then reused for the subsequent photoisomerization cycle. This photoisomerization/catalytic back-conversion sequence was repeated for six consecutive cycles. Following each catalytic cycle, the recovered solution after centrifugation was analysed by X-ray fluorescence to quantify the concentration of platinum species present in solution and thereby assess the extent of Pt leaching. Samples of the fresh catalyst before the first cycle and the recovered catalyst after the sixth cycle were analysed by ICP-OES to determine changes in the platinum content of the catalyst following repeated cycling.

### NMR analysis

The conversions of the reactions were calculated directly from the integrals at 7.75 ppm belonging to the NBD species and 7.15 ppm belonging to the QC species. As both these environments correspond to 2 equivalent protons the following simplified equation was used:

%NBD = $\frac{I\mathrm{NBD}}{I\mathrm{QC} + I\mathrm{NBD}}$ x 100

Where I_NBD_ is the integral for NBD species and I_QC_ is the integral for the QC species.

After UVA: 2 / (2 + 110) x100 = 1.79 % and after catalysis: 2 / (2 + 0.14) x100 = 93.5 %

**Figure S72.** After sixth photoisomerisation reaction the peaks at 7.75 ppm and 7.15 ppm were analysed as they both represent 2H environments from NBD and QC species respectively.

**Figure S73.** After sixth catalytic reaction the peaks at 7.75 ppm and 7.15 ppm were analysed as they both represent 2H environments from NBD and QC species respectively.

### X-ray fluorescence (XRF)

X-ray fluorescence (XRF) analyses were performed using an XEPOS energy-dispersive XRF spectrometer. The instrument was operated at an accelerating voltage of 40 kV and a tube current of 0.63 mA. Each sample was measured for 600 s. A molybdenum (Mo) X-ray tube was used as the excitation source, and the spectra were evaluated using the Lα peak with a Serien Fit deconvolution method.

**Figure S74.** X-ray fluorescence (XRF) spectra of five platinum standards of 2, 5, 10, 15, and 20 ppm in propylene carbonate matrix with L-Alpha and L-Beta.

Following each catalytic cycle, the recovered solution after centrifugation was analyzed by XRF (as could not measure using ICP-OES platinum content within an organic phase) to quantify the concentration of platinum species present in solution and thereby assess the extent of Pt leaching.

| Concentration (ppm) | Normalised intensities (a.u) |
| --- | --- |
| 2 | 16.876 |
| 5 | 41.5171 |
| 10 | 59.2566 |
| 15 | 69.8062 |
| 20 | 101.459 |

**Figure S75.** Calibration plot of platinum in propylene carbonate matrix recorded by XRF.

A total content of 10.5 ppm of platinum is leached from the catalyst into the solution.

**Table S15.** Platinum content leached into propylene carbonate solution after each catalytic reaction.

| **Cycle** | **Absolute intensities (a.u)** | **Concentration (ppm)** | **Error** |
| --- | --- | --- | --- |
| 1 | 25.9 | 2.9 | 0.1 |
| 2 | 14.5 | 0.2 | 0.1 |
| 3 | 14.8 | 0.2 | 0.1 |
| 4 | 25.9 | 2.9 | 0.1 |
| 5 | 29.3 | 3.7 | 0.1 |
| 6 | 16.2 | 0.6 | 0.1 |

### ICP-OES

The platinum (Pt) concentration in the catalyst samples was determined using an Agilent 5100 ICP-OES spectrometer. Prior to analysis, the catalyst samples were digested using an appropriate acid digestion procedure, and the Pt concentration was quantified against external calibration standards. Samples of the fresh catalyst before the first cycle and the recovered catalyst after the final cycle were digested and analyzed by ICP-OES to determine changes in the platinum content of the catalyst following repeated cycling.

| Concentration (ppm) | Intensity (a.u) |
| --- | --- |
| 0 | -18 |
| 2 | 2252 |
| 5 | 6181 |
| 10 | 9329 |
| 15 | 18220 |
| 20 | 24206 |

**Figure S76.** Calibration plot of platinum was conducted between 0-20 ppm.

The weighed amount of catalyst beforehand was 10.1 mg. The sample was digested in 10 mL of aqua regia, 250 µl of this solution was taken and diluted into 5 mL of 0.2 M HNO_3_ solution, prior to measurement. The weighed amount of catalyst after drying the propylene carbonate overnight in an oven at 250 ⁰ C was 8.9 mg. The sample was digested in 10 mL of aqua regia, 250 µl of this solution was taken and diluted into 5 mL of 0.2 M HNO_3_ solution, prior to measurement. The Pt light source used was at the wavelength 265.945 the ppm measured before was 1.22168242 and after 0.886843701.

**Table S16.** ICP-OES measurements and results.

| **Sample** | **Measured (ppm)** | **Before dilution (ppm)** | **Mg of Pt** | **Weight % of Pt** |
| --- | --- | --- | --- | --- |
| Before | 1.22168242 | 24.433648 | 0.24433648 | 2.4192 |
| After | 0.886843701 | 17.736874 | 0.17736874 | 1.9929 |

A total of approximately **6.7 ppm** of platinum was determined to have leached from the catalyst. This value differs only slightly from the XRF result, which indicated a total platinum leaching of **10.5 ppm** into the solution. The discrepancy is likely due to differences in the sensitivity of the analytical instruments, as well as potential variations in the dispersion and homogeneity of the catalyst samples analyzed by each technique.

The weight % of platinum lost from the catalyst after six cycling experiments: 2.4192 − 1.9929 = 0.4263 wt% Pt.

Approximately 17.62 % of initial platinum loading was leached from the catalyst after six cycles, including centrifugation steps to separate reaction mixture with suspended catalyst particles.

Percent loss = $\frac{0.4263}{2.4192}$. × 100 = 17.62%

### X-ray photoelectron spectroscopy (XPS)

XPS experiments were performed in a SPECS system with a PHOIBOS 150 EP Hemispherical Energy Analyzer with MCD-9 detector using an X-ray source Al Kα line of 1486.6 eV energy and 150 W power and using a pass energy of 20eV, placed at 54 degrees respect to the analyzer axis and calibrated by the 3d5/2 line of Ag with a full width at half maximum (FWHM) of 1.211eV. XPS data of the catalyst batch before and after the heat release experiment.

**Table S17**. Overview of XPS survey analysis of the catalyst before and after Pt deposition. The table summarizes the binding energies, full width at half maximum (FWHM), integrated peak areas, and surface atomic concentrations (%At. Conc.) of the detected O 1s, C 1s, and Pt 4f signals.

| **Sample Id** | **Name** | **Position** | **FWHM** | **Raw Area** | **%At Conc** |
| --- | --- | --- | --- | --- | --- |
| **Catalyst after** | O 1s | 531.4 | 4.15 | 5220.12 | **22.8** |
|  | C 1s | 284.6 | 2.01 | 5990.32 | **76.6** |
|  | Pt 4f | 72.8 | 5.82 | 791.936 | **0.7** |
| **Catalyst before** | O 1s | 531.4 | 3.76 | 8031.44 | **28.7** |
|  | C 1s | 284.8 | 2.6 | 6753.68 | **70.8** |
|  | Pt 4f | 75.8 | 5.12 | 699.925 | **0.5** |

**XPS spectra and data before catalytic reaction.**

**Figure S77.** Pt 4f XPS spectrum (red) showing the overall fit (dark red), deconvoluted components assigned in Table S18.

**Table S18**. Peak fitting results of the Pt 4f XPS spectrum for the Pt-loaded catalyst, including binding energy (Position), full width at half maximum (FWHM), raw peak area, RSF-corrected peak area, atomic concentration, and the calculated relative abundance of Pt(II) and Pt(IV) species.

| **Name** | **Position** | | **FWHM** | **Raw Area** | | **Area/(RSF*T*MFP)** | **%At Conc** |
| --- | --- | --- | --- | --- | --- | --- | --- |
| PtO | 72 | | 3 | 302 | | 34.9105 | 36.4 |
| PtO | 76 | | 3 | 226 | | 33.3062 | 34.7 |
| PtO2 | 75 | | 3 | 123 | | 14.2013 | 14.8 |
| PtO2 | 79 | | 3 | 92 | | 13.5486 | 14.1 |
|  |  | |  |  | |  |  |
| Pt(II) | | **71.1 %** | | |  |  |  |
| Pt(IV) | | **28.9 %** | | |  |  |  |

**Figure S78.** C 1s XPS spectrum (red) showing the overall fit (dark red) and deconvoluted components assigned in Table S19.

**Table S19**. Peak fitting results of the C 1s XPS spectrum for the catalyst, including the assigned carbon species, binding energies (Position), full width at half maximum (FWHM), raw and corrected peak areas, and the corresponding atomic concentrations.

| **Name** | **Position** | **FWHM** | **Raw Area** | **Area/(RSF*T*MFP)** | **%At Conc** |
| --- | --- | --- | --- | --- | --- |
| sp2 | 284.55 | 1.46 | 2652.2 | 2652.2 | 41.0 |
| sp3 | 285.5 | 1.46 | 1627.14 | 1627.14 | 25.2 |
| C-O | 286.39 | 1.46 | 772.437 | 772.437 | 12.0 |
| C=O | 287.55 | 2.5 | 918.355 | 918.355 | 14.2 |
| O-C=O | 289.92 | 2.5 | 495.078 | 495.078 | 7.7 |

**Figure S79.** O 1s XPS spectrum (red) showing the overall fit (dark red) and deconvoluted components assigned in Table S20.

**Table S20**. XPS fitting parameters for the O 1s region of the catalyst. The O 1s spectrum was deconvoluted into three oxygen-containing components, and the corresponding binding energies, FWHM values, integrated peak areas, corrected peak areas, and relative atomic concentrations are summarized.

| **Name** | **Position** | **FWHM** | **Raw Area** | **Area/(RSF*T*MFP)** | **%At Conc** |
| --- | --- | --- | --- | --- | --- |
| O 1s | 530.69 | 2.2 | 3820.55 | 1303.94 | 47.8 |
| O 1s | 532 | 2.2 | 3061.06 | 1044.73 | 38.3 |
| O 1s | 533.88 | 2.2 | 1119.95 | 382.236 | 14.0 |

**XPS spectra and data after catalytic reaction.**

**Figure S80.** Pt 4f XPS spectrum (red) showing the overall fit (dark red), deconvoluted components assigned in Table S21.

**Table S21**. Peak fitting results of the Pt 4f XPS spectrum for the Pt-loaded catalyst, including binding energy (Position), full width at half maximum (FWHM), raw peak area, RSF-corrected peak area, atomic concentration, and the calculated relative abundance of Pt(II) and Pt(IV) species.

| **Name** | **Position** | | **FWHM** | **Raw Area** | | **Area/(RSF*T*MFP)** | **%At Conc** |
| --- | --- | --- | --- | --- | --- | --- | --- |
| PtO | 72.37 | | 2.58 | 314.038 | | 36.3049 | 35.5 |
| PtO | 75.72 | | 2.58 | 235.528 | | 34.6365 | 33.8 |
| PtO2 | 74.36 | | 3 | 139 | | 16.0722 | 15.7 |
| PtO2 | 77.71 | | 3 | 104 | | 15.3336 | 15.0 |
|  |  | |  |  | |  |  |
| Pt(II) | | **69.3 %** | | |  |  |  |
| Pt(IV) | | **30.7 %** | | |  |  |  |

**Figure S81.** C 1s XPS spectrum (red) showing the overall fit (dark red) and deconvoluted components assigned in Table S22.

**Table S22**. Peak fitting results of the C 1s XPS spectrum for the catalyst, including the assigned carbon species, binding energies (Position), full width at half maximum (FWHM), raw and corrected peak areas, and the corresponding atomic concentrations.

| **Name** | **Position** | **FWHM** | **Raw Area** | **Area/(RSF*T*MFP)** | **%At Conc** |
| --- | --- | --- | --- | --- | --- |
| sp2 | 284.5 | 1.41 | 3064.35 | 3064.35 | 54.1 |
| sp3 | 285.6 | 1.41 | 943.118 | 943.118 | 16.7 |
| C-O | 286.47 | 1.41 | 427.193 | 427.193 | 7.6 |
| C=O | 287.6 | 2.5 | 802.069 | 802.069 | 14.2 |
| O-C=O | 290.23 | 2.5 | 422.894 | 422.894 | 7.5 |

**Figure S82.** O 1s XPS spectrum (red) showing the overall fit (dark red) and deconvoluted components assigned in Table S23.

**Table S23**. XPS fitting parameters for the O 1s region of the catalyst. The O 1s spectrum was deconvoluted into three oxygen-containing components, and the corresponding binding energies, FWHM values, integrated peak areas, corrected peak areas, and relative atomic concentrations are summarized.

| **Name** | **Position** | **FWHM** | **Raw Area** | **Area/(RSF*T*MFP)** | **%At Conc** |
| --- | --- | --- | --- | --- | --- |
| O 1s | 530.74 | 2.26 | 2395.88 | 817.707 | 46.1 |
| O 1s | 532.55 | 2.26 | 2038.16 | 695.618 | 39.2 |
| O 1s | 534.04 | 2.26 | 767.502 | 261.946 | 14.8 |

4	References

1. C. M. Hansen in Hansen Solubility Parameters: A User’s Handbook, second edition, CRC Press, Boca Raton, **2007**.
2. https://www.stenutz.eu/chem/solv2.html
3. U. Mayer, V. Gutmann, W. Gerger, *Monat. Chem.* **1975**, 106, 1235.
4. M. Beckett, G. Strickland, *Polym. Commun.* **1996**, 37, 4629.
5. Libra, J. KolXPD: Spectroscopy data measurement and processing.

   http://www.kolibrik.net/science/kolxpd/ (accessed in October 2025)
6. https://www.chemeo.com/cid/15-970-8/Propylene-Carbonate
7. S. Sugiura, Y. Shintani, S. L. Higashi, A. Shibata, M. Ikeda, ChemRxiv, **2023**.
8. H. Piekarski, K. Kubalczyk, M. Wasiak, *J. Chem. Eng. Data*., **2010**, 55, 5435-5440.
9. E. Pensini, A. G. Marangoni, A. Correa, G. Milano, S. Prevost, *J. Phys. Chem. B.,* **2025**, 129, 5578-5590.

   ^10^ F. Castro, J. S. Gancheff, J. C. Ramos, G. Seoane, C. Bazzicalupi, A. Bianchi, F. Ridi, M. Savastano, *Molecules*., **2023**, 28, 7270.

   ^11^ F. Castro, J. C. Ramos, G. Seoane, J. S. Gancheff, C. Bazzicalupi, A. Bianchi, F. Ridi, M. Savastano, *Sol. Energy.,* **2026**, 304, 114167.

   ^12^ S. Hernaez-Troya, N. Sanosa, A. Gimenez-Gomez, V. Pozo-Gavara, D. Sampedro, I. Funes-Ardoiz, *Angew. Chem. Int. Ed*., **2025**, 64, e202514349.

   ^13^ L. Fernandez, H. Hölzel, P. Ferreira, N. Baggi, K. Moreno, Z. Wang, K. Moth-Poulsen, *Green. Chem.,* **2025**, 27, 14119-14130.

   ^14^ H. P. Q. Nguyen, A. J. Maertens, B. A. Baker, N. M.-W. Wu, G. G. D. Han, *Science*., **2026**, 392, 6796.
10. ^15^ C. G. Hatchard, C. A. Parker, *R. Soc. Open. Sci.,* **1956**, 235, 518-536.

    ^16^ L. Magson, H. Hölzel, A. S. Aslam, S. Henninger, G. Munz, K. Moth-Poulsen, M. Knaebbeler-Buss, I. Funes-Ardoiz and D. Sampedro, ACS Appl. Mater. Interfaces, **2024**, 16, 7211–7218.

    ^17^ Z. Wang, A. Roffey, R. Losantos, A. Lennartson, M. Jevric, A. U. Petersen, M. Quant, A. Dreos, X. Wen, D. Sampedro, K. Börjesson, K. Moth-Poulsen, Energy. Environ. Sci. **2019**, 12, 187-193.
